# Identification and functional analysis of mitogen-activated protein kinase kinase kinase (MAPKKK) genes in canola (*Brassica napus* L.)

**DOI:** 10.1093/jxb/eru092

**Published:** 2014-03-06

**Authors:** Yun Sun, Chen Wang, Bo Yang, Feifei Wu, Xueyu Hao, Wanwan Liang, Fangfang Niu, Jingli Yan, Hanfeng Zhang, Boya Wang, Michael K. Deyholos, Yuan-Qing Jiang

**Affiliations:** ^1^State Key Laboratory of Crop Stress Biology for Arid Areas and College of Life Sciences, Northwest A & F University, Yangling, Shaanxi 712100, China; ^2^Department of Biological Sciences, University of Alberta, Edmonton T6G 2E9, Canada

**Keywords:** Abiotic stress, *Brassica napus*, cell death, MAPKKK, MKK, *Sclerotinia sclerotiorum*.

## Abstract

Mitogen-activated protein kinase (MAPK) signalling cascades, consisting of three types of reversibly phosphorylated kinases (MAPKKK, MAPKK, and MAPK), are involved in important processes including plant immunity and hormone responses. The MAPKKKs comprise the largest family in the MAPK cascades, yet only a few of these genes have been associated with physiological functions, even in the model plant *Arabidopsis thaliana*. Canola (*Brassica napus* L.) is one of the most important oilseed crops in China and worldwide. To explore MAPKKK functions in biotic and abiotic stress responses in canola, 66 MAPKKK genes were identified and 28 of them were cloned. Phylogenetic analysis of these canola MAPKKKs with homologous genes from representative species classified them into three groups (A–C), comprising four MAPKKKs, seven ZIKs, and 17 Raf genes. A further 15 interaction pairs between these MAPKKKs and the downstream BnaMKKs were identified through a yeast two-hybrid assay. The interactions were further validated through bimolecular fluorescence complementation (BiFC) analysis. In addition, by quantitative real-time reverse transcription–PCR, it was further observed that some of these *BnaMAPKKK* genes were regulated by different hormone stimuli, abiotic stresses, or fungal pathogen treatments. Interestingly, two novel *BnaMAPKKK* genes, *BnaMAPKKK18* and *BnaMAPKKK19*, which could elicit hypersensitive response (HR)-like cell death when transiently expressed in *Nicotiana benthamiana* leaves, were successfully identified. Moreover, it was found that BnaMAPKKK19 probably mediated cell death through BnaMKK9. Overall, the present work has laid the foundation for further characterization of this important *MAPKKK* gene family in canola.

## Summary

Twenty-eight *BnaMAPKKK* genes were cloned. Phylogenetic and expression profiling analyses indicated their relationship and roles in stress and hormone signalling. Two novel *BnaMAPKKK* genes were identified to mediate cell death independent of pathogens.

## Introduction

To survive harsh conditions, plants have developed sophisticated mechanisms to sense environmental cues and transmit these signals to regulate plant development and defence. Plant mitogen-activated protein kinase (MAPK) cascades are vital to these functions (Asai *et al.*, 2002; Ichimura *et al.*, 2002; Rodriguez *et al.*, 2010; Meng and Zhang, 2013). MAPK cascades are conserved in eukaryotes through evolution and are composed of MAPKK kinases (MAPKKKs, MAP3K, or MEKK), MAPK kinases (MAPKKs, MAP2Ks, MKKs, or MEKs), and MAPKs (MPKs). Basically, extracellular stimuli sensed by receptors are sequentially transmitted through phosphorylation by MAPKKKs to MKKs, and then to MPKs to regulate intracellular responses including the transcriptional and metabolic responses (Hamel *et al.*, 2006). The activated MAPKKKs phosphorylate either the serine (S) or threonine (T) residues of MKKs and the activated MKKs phosphorylate both T and tyrosine (Y) residues of the MPKs (Asai *et al.*, 2002).

Several *MKK* and *MPK* genes have been well characterized, including a description of their downstream components and the physiological processes they mediate (Frye *et al.*, 2001; Asai *et al.*, 2002; Jin *et al.*, 2002; del Pozo *et al.*, 2004; Liu *et al.*, 2004; Nakagami *et al.*, 2004; Colcombet and Hirt, 2008; Melech-Bonfil and Sessa, 2010; Ning *et al.*, 2010; Oh *et al.*, 2010). However, only a limited number of *MAPKKK* genes have been functionally characterized (Rodriguez *et al.*, 2010; Meng and Zhang, 2013). A possible reason underlying this is that MAPKKKs form a large gene family, making functional redundancy inevitable. In *Arabidopsis*, MAPKKK is the largest group of the MAPK cascade components, with 80 members classified into three subfamilies, MEKK, Raf, and ZIK, harbouring 21, 11, and 48 genes, respectively (Jonak *et al.*, 2002). The MEKK subfamily is the best characterized and includes tobacco NPK1 (Jin *et al.*, 2002; Liu *et al.*, 2004), *Arabidopsis* MEKK1 (Asai *et al.*, 2002), alfalfa OMTK1 (oxidative stress activated MAPK triple-kinase 1, MEKK1) (Nakagami *et al.*, 2004), tobacco NbMAPKKKα, NbMAPKKKγ, and NbMAPKKKε, and tomato SlMAPKKKα and SlMAPKKKε (del Pozo *et al.*, 2004; Melech-Bonfil and Sessa, 2010; Oh *et al.*, 2010). Characterized genes of the second subfamily of MAPKKKs include *Arabidopsis* CTR1/Raf1 (Kieber *et al.*, 1993; Clark *et al.*, 1998), EDR1/Raf2 (Frye *et al.*, 2001), and rice (*Oryza sativa*) DSM1 (Ning *et al.*, 2010). The ZIK subfamily, also called WNK (With No lysine Kinase), is reported to regulate flowering time and circadian rhythms in rice and *Arabidopsis* (Wang *et al.*, 2008; Kumar *et al.*, 2011).

Once signals are transmitted from MAPKKKs to terminal MPKs through sequential phosphorylation, activated MPKs then phosphorylate a wide range of substrates, such as WRKY transcription factors or enzymes (Rodriguez *et al.*, 2010; Liang *et al.*, 2013). The first identified complete signalling module is the FLS2–MEKK1–MKK4/5–MPK3/6–WRKY22/29 pathway in *Arabidopsis*, which is effective against different pathogens including bacteria and the fungal pathogen *Botrytis cinerea* (Asai *et al.*, 2002; Galletti *et al.*, 2011). Another module, MEKK1–MKK1/2–MPK4, of *Arabidopsis* was shown to regulate defence responses against biotrophic pathogens negatively while positively regulating defences against necrotrophic fungi (Petersen *et al.*, 2000; Ichimura *et al.*, 2006; Qiu *et al.*, 2008). The induction of salicylic acid (SA) as well as systemic acquired resistance (SAR) in *mpk4*, *mekk1*, or *mkk1mkk2* double mutants is a result of release and activation of WRKY25 and -33 in nuclei by MAPK substrate 1 (MKS1) in *Arabidopsis* (Andreasson *et al.*, 2005; Petersen *et al.*, 2010). Further study found that AtMPK4 could induce camelexin biosynthesis upon challenge by a bacterial pathogen (Andreasson *et al.*, 2005; Ichimura *et al.*, 2006; Qiu *et al.*, 2008) but not by a fungal pathogen (Mao *et al.*, 2011). Recently, it was found that AtMEKK1 and AtMKK1/MKK2 negatively regulate MEKK2-mediated immune responses as well as programmed cell death (PCD) (Kong *et al.*, 2012). In tobacco, NPK1–MEK1–Ntf6 mediate resistance to *Tobacco mosaic virus* (TMV) triggered by the R protein N (Jin *et al.*, 2002; Liu *et al.*, 2004). Moreover, AtEDR1, a Raf-like MAPKKK, could function at the top of a MAPK cascade to regulate SA-inducible defence responses negatively (Frye *et al.*, 2001). However, the functions of most other *MAPKKK* genes in *Arabidopsis* are as yet unknown.

Though investigations of the *MAPKKK* gene family in *Arabidopsis*, rice, and maize have been reported in recent years (Jouannic *et al.*, 1999; Singh *et al.*, 2012; Kong *et al.*, 2013), no similar study has yet been conducted in canola. Canola is a very important oil crop worldwide and its yield is frequently limited by environmental factors including drought, salinity, cold, and biotic factors, such as stem rot caused by *Sclerotinia sclerotiorum*. *Sclerotinia sclerotiorum* is a necrotrophic pathogen and no efficient way has been identified to control this disease. An oxidative burst, or accumulation of reactive oxygen species (ROS), has been associated with many abiotic stresses and pathogen infections (Jaspers and Kangasjarvi, 2010; Heller and Tudzynski, 2011), especially *S. sclerotiorum* (Rietz *et al.*, 2012). Research with MAPK cascades in *Arabidopsis* has shown that they are involved in signalling multiple defence responses, including the biosynthesis and signalling of plant stress/defence hormones, ROS production, stomatal closure, defence gene activation, phytoalexin biosynthesis, cell wall strengthening, and hypersensitive response (HR) cell death (Meng and Zhang, 2013). However, the identity and role of *MAPKKK* genes in canola responses to abiotic and biotic stresses are unknown. It is therefore necessary to characterize the *MAPKKK* gene family in canola before stress/disease-tolerant canola species can be developed. In previous transcriptomic profiling studies in canola, a few components of the MAPK module were identified, including several *MAPKKK* genes elicited by *S. sclerotiorum* (Yang *et al.*, 2007). A few novel *BnaMKK* (Bna for *Brassica napus*) and *BnaMPK* genes and modules were further characterized (Liang *et al.*, 2013). The functions of some of the *MKK*, *MPK*, and *WRKY* genes are under investigation through loss-of-function and gain-of-function strategies. However, the upstream components of MKK–MAPK modules, which are BnaMAPKKKs, have not yet been characterized. Hence, to explore the role of *MAPKKK* genes in immune and abiotic stress responses in canola, the publicly available expressed sequence tags (ESTs) were mined to identify *MAPKKK* genes in canola. The cDNA sequences of 28 *BnaMAPKKK* genes were then cloned, followed by a yeast two-hybrid (Y2H)-based analysis of interactions between canola MAPKKKs and MKKs, and part of the interactions were confirmed *in planta*. The responses of selected genes under a range of abiotic and biotic stress conditions were also examined. Interestingly, two novel *BnaMAPKKK* genes that could elicit cell death when transiently expressed in tobacco leaves were successfully identified. These may mediate cell death by regulating specific downstream MKKs. To the authors’ knowledge, this is the first report of canola *MAPKKK* genes, and the data presented here will lay the foundation for further characterization of this important *MAPKKK* gene family in canola responses to abiotic and biotic stresses.

## Materials and methods

### Database search and identification of *MAPKKK* ESTs in canola

The identification of canola ESTs representing *MAPKKK* genes was performed as described previously (Liang *et al.*, 2013). In brief, ESTs representing MAPKKKs were retrieved through BLAST search in the NCBI dbEST (http://www.ncbi.nlm.nih.gov/dbEST/index.html, release 01012012) and DFCI (http://compbio.dfci.harvard.edu/tgi/cgi-bin/tgi/gimain.pl?gudb=oilseed_rape,release5.0) using the 80 public AtMAPKKK cDNA sequences downloaded from TAIR9.0 (www.arabidopsis.org) as the queries with an e-value cut-off <10^–4^. After manual curation, these ESTs were clustered and assembled by DNASTAR (DNASTAR Inc., USA). Subsequently, contigs and singletons were run in a reciprocal BLAST search against the *Arabidopsis* database to assign a putative orthologue based on the best hit (Supplementary Table S1 available at *JXB* online).

### Plant growth and gene cloning

Canola (double haploid DH12075) plants were grown as described previously (Liang *et al.*, 2013). RNA was isolated using the Plant RNA kit (Omega bio-tek, USA). First-strand cDNA synthesis and high-fidelity PCR amplification using PrimeSTAR HS DNA polymerase (TaKaRa, Japan) were performed as previously described (Liang *et al.*, 2013) with the primers listed in Supplementary Table S2 available at *JXB* online. PCR products were purified and cloned into the pJET1.2 vector supplied in the CloneJET PCR cloning kit (Fermentas, USA), sequenced, and analysed by DNASTAR. The cDNA sequences of genes cloned in this study were deposited in the GenBank database under the accession numbers KC190095–KC190110 and KF129395–KF129406.

### Phylogenetic tree reconstruction, multiple alignment analysis, and conserved signature detection

The *MAPKKK* genes of rice were downloaded from the rice genome annotation project (http://rice.plantbiology.msu.edu), while those of other species were either identified from Phytozome v9.1 (http://www.phytozome.net/) or retrieved from the NCBI by a keyword search (Supplementary Table S3 available at *JXB* online). To investigate the evolutionary relationship among MAPKKKs, the predicted amino acid sequences of the products of the *MAPKKK* genes of canola and other species were aligned and a phylogenetic tree was reconstructed as described previously (Liang *et al.*, 2013). Motif analysis of BnaMAPKKKs was determined by using the Prosite program (http://prosite.expasy.org/prosite.html), and a schematic diagram of amino acid motifs of each BnaMAPKKK was drawn accordingly. The respective domains of MAPKKK proteins were aligned using ClutsalX1.83 and analysed in MEME 4.9.0 (release date 3 October 3, 11:07:26 EST 2012) or illustrated by Boxshade (http://www.ch.embnet.org/software/BOX_form.html). The percentage identity and similarity of sequences was calculated using the program MatGAT v2.02 (Campanella *et al.*, 2003).

### Subcellular localization and confocal microscopy

To examine the localization of selected BnaMAPKKKs *in planta*, the coding regions were amplified using *Pfu* polymerase (Bioer, China) with the primers listed in Supplementary Table S2 available at *JXB* online. These were digested by the corresponding restriction enzymes and fused upstream of the green fluorescent protein gene (*GFP*) in the pYJGFP vector. These constructs and p19 protein of *Tomato bushy stunt virus* were transformed into *Agrobacterium tumefaciens* GV3101 individually, and overnight cell cultures were resuspended in infiltration media before being infiltrated into 5-week-old leaves of *Nicotiana benthamiana* (Liang *et al.*, 2013). Two days later, leaf discs were plasmolysed with 500mM mannitol or not treated, and observation of GFP was conducted under an A1R confocal microscope (Nikon, Japan).

### Quantitative reverse transcription–PCR (qRT–PCR) assay

Eighteen-day-old canola grown in a greenhouse with a photoperiod of 16h light/8h dark were treated with *S. sclerotiorum* inoculation, 5mM oxalic acid (OA; Sigma-Aldrich) inoculation, and agar inoculation as a control for *S. sclerotiorum* and OA treatment. Different chemical treatments include 200mM NaCl, 100 μM jasmonic acid (JA; Sigma-Aldrich, USA), 2mM SA (Sigma-Aldrich), 50 μM abscisic acid (±-ABA; Invitrogen, USA), 25 μM 1-aminocyclopropane-1-carboxylic acid (ACC; Sigma-Aldrich), 10 μM methyl viologen (MV; Sigma-Aldrich); alternatively, plants were treated at 4 ºC (cold) and 37 ºC (heat), and mock-treated plants were used as the control (Liang *et al.*, 2013). Leaves were collected at 1h (except *S. sclerotiorum* and OA, which were collected after 3h) and 24h post-treatments, flash frozen in liquid nitrogen, and stored at –80 °C. Total RNA samples were isolated and the first-strand cDNAs were synthesized from 2.5 μg of total RNA as described previously (Liang *et al.*, 2013). Three independent biological replicates of each sample were prepared at different times.

qRT–PCR was performed using 10-fold diluted cDNA and a SYBR Green I kit (CWBIO, China) on a CFX96 real-time PCR machine (Bio-Rad, USA). Primers used for qRT–PCR were designed using the PrimerSelect program (DNASTAR Inc.), which was targeted mainly at the 3’-untranslated region (UTR) with an amplicon size of 75–250bp (Supplementary Table S2 available at *JXB* online). The specificity and amplification efficiency of each pair of primers were examined through both a BLASTn search in the NCBI database and by running standard curves with melting curves. Three independent biological replicates and two technical replicates for each biological replicate were run and the significance was determined through *t-*test of SPSS statistical software (*P*<0.05).

### Yeast two-hybrid assay

The coding regions of canola *MAPKKK* and *MKK* genes were subcloned into pGBKT7 (BD) and pGADT7 (AD) vectors, respectively, using the primers listed in Supplementary Table S2 available at *JXB* online. Then recombinant plasmids were transformed sequentially into yeast AH109 competent cells according to the Yeast Protocols Handbook (Clontech, USA). The interactions between BnaMAPKKKs and BnaMKKs were tested by streaking both on non-selective SD-LW (synthetic dropout without leucine and tryptophan), and on selective SD-LWH (SD–Leu–Trp–His+5mM 3’AT) and SD-LWHA (-SD-Leu-Trp-His-Ade) media. Plates were incubated at 30 ºC for up to 7 d before being photographed. The titration and colony-lift filter assays were conducted as described previously (Liang *et al.*, 2013).

### Bimolecular fluorescence complementation (BiFC)

To verify interaction partners *in planta*, yellow fluorescent protein (YFP)-based BiFC analysis was performed. The coding regions of *BnaMAPKKK* and *BnaMKK* genes were subcloned into pSPYNE(R)173 and pSPYCE(M) vectors, respectively (Waadt *et al.*, 2008). Primers used are listed in Supplementary Table S2 available at *JXB* online. The recombinant plasmids were transformed into *Agrobacterium* GV3101 competent cells before being used to infiltrate the leaves of 5-week-old *N. benthamiana* plants as described previously (Liang *et al.*, 2013). Three to four days later, YFP signals in at least three slides were examined under an A1R confocal microscope (Nikon, Japan).

### Site-directed mutagenesis

The coding regions of relevant *BnaMAPKKK* and *BnaMKK* genes were PCR amplified using *Pfu* polymerase and Gateway-compatible gene-specific primers (Supplementary Table S2 available at *JXB* online) before being introduced into a Gateway entry vector pDONOR/Zeo (Invitrogen, USA). For substitution of one amino acid residue with another, two overlapping primers harbouring mutated nucleotides in the middle were used to run PrimeSTAR-mediated PCR (Li and Wilkinson, 1997). The BnaM3K18-K32G-F/BnaM3K18-K32G-R and BnaM3K19-K37G-F/BnaM3K19-K37G-R primers were used to change the lysine (K) at residues 32 and 37 to glycine (G), respectively (Melech-Bonfil and Sessa, 2010; Hashimoto *et al.*, 2012). Primers BnaMKK9-K74R-F and BnaMKK9-K74R-R were designed to mutate lysine (K) at residue 74 to arginine (R), while primers of BnaMKK9-S193D/S199E-F and BnaMKK9-S193D/S199E-R were designed to mutate serine (S) on residues 193 and 199 to aspartic acid (D) and glutamic acid (E) individually. BnaMKK9^K74R^ and BnaMKK9^S193D/S199E^ are constitutively inactive and active forms of BnaMKK9, respectively (Popescu *et al.*, 2009). PCR products were purified, followed by *Dpn*I (Fermentas, USA) digestion overnight. After purification, the restricted PCR product was transformed into *Escherichia coli* DH5α competent cells and was selected on low-salt LB medium supplemented with 50mg l^–1^ zeocin, with the plasmid isolated and sequenced to confirm that the mutated regions were correct.

### Transient expression and physiological assay

The coding regions of the respective genes and their mutated derivates were isolated by restriction digestion of the aforementioned pJET or pDONR/Zeo recombinant plasmids, which was sometimes preceded by PCR amplification using high-fidelity *Pfu* polymerase and primers containing corresponding restriction sites, as listed in Supplementary Table S2 available at *JXB* online. After digestion, the products were inserted downstream of a double *Cauliflower mosaic virus* (CaMV) 35S promoter in the binary vector pYBHA or pYBMyc, which was modified from the pYJGFP vector (Liang *et al.*, 2013). Recombinant plasmids were transformed into *A. tumefaciens* GV3101 and overnight cell cultures were resuspended in infiltration media containing 10mM MES-KOH (pH 5.6), 10mM MgCl_2_, and 0.15mM acetosyringone, adjusted to an OD_600_ of 0.5 before equal volumes of each cell culture were infiltrated into the lower epidermal side of 4-week-old leaves of *N. benthamiana* plants. For each construct, 21 independent leaves of seven independent plants (three leaves per plant) were used for each time point tested. After that, infiltrated plants were kept under normal growth conditions with the phenotype observed and recorded daily beginning 2 d after infiltration and continuing until day 7. To quantify the degree of PCD, electrolyte leakage was measured according to Oh and Martin (2011) with modifications. In brief, five leaf discs (10mm in diameter) were taken from each agro-infiltrated area and kept in deionized water under vacuum for 10min, followed by incubation for 30 min at 25 °C. Ion conductivity was measured using a DDS-307 ion conductivity meter (Leici, China). After boiling for 5min and cooling to room temperature, the ion conductivity was measured again. Distribution of hydrogen peroxide (H_2_O_2_) was detected by 3,3’-diaminobenzidine (DAB) staining according to Daudi *et al.* (2012).

## Results and Discussion

### Identification and cloning of *MAPKKK* genes from canola

Although the functions of a few *MAPKKK* genes in *Arabidopsis* and a few other plant species have been reported, little is known about this gene family in the important oilseed crop, canola (*B. napus*). A previous transcriptomic study identified several pathogen- or defence hormone-responsive *MAPKKK* genes, including *BnaMAPKKK17* and *BnaMAPKKK18* (Yang *et al.*, 2007), suggesting that MAPKKKs may play a role in canola defence against fungal pathogens. More recently, as a follow-up work, *MKK* and *MAPK* genes, as well as the downstream WRKY transcription factor genes in canola were systemically studied and part of them were characterized (Yang *et al.*, 2009; Liang *et al.*, 2013). Ongoing work with selected components of MKK–MPK–WRKY cascades in canola demonstrated interesting phenotypes of gain- and loss-of-function plants (unpublished data). However, the upper components of MAPK cascades, namely MAPKKKs, have not yet been described in canola. All of these prompted the authors to clone and study them in the context of abiotic and biotic stress conditions. To this end, public EST databases were mined, since whole-genome sequencing of *B. napus* is not yet complete. Eighty cDNA sequences of *AtMAPKKK* genes were used to search for ESTs of canola that showed high similarity to *AtMAPKKK* genes. Altogether 839 unique ESTs were obtained including 80 singletons and 145 contigs representing putative *MAPKKK* genes in canola (Supplementary Table S1 available at *JXB* online). To facilitate comparisons between species, the established nomenclature of *AtMAPKKK* genes was followed when naming the *BnaMAPKKK* (*Brassica napus MAPKKK*) genes. As a result, 66 *MAPKKK* genes from canola were identified, which are composed of 18 *MAPKKK* genes, nine *ZIK* genes, and 39 *Raf* genes. It was noted that among all the *BnaMAPKKK* genes identified, *BnaRaf28* has the largest number (73) of ESTs, followed by *BnRaf22* and *BnRaf21* with a total of 63 and 51 ESTs, respectively; while *BnaMAPKKK2*, *BnaMAPKKK19*, *ZIK3*, *ZIK7*, *Raf2*, and *Raf7* have only one EST each (Table 1; Supplementary Table S1 available at *JXB* online). To facilitate the following work, primers were designed based on the identified ESTs to obtain full-length cDNA sequences, at least for the coding regions, employing RT–PCR together with rapid amplification of cDNA ends (RACE). As a result, the cloning of cDNA sequences of 28 *BnaMAPKKK* genes was achieved (Table 1). Conceptual translation of these cDNA sequences and reciprocal BLAST searches against the *Arabidopsis* genome indicated that they bore domains and motifs that were typical of MAPKKK proteins. The number of amino acids in each BnaMAPKKK protein ranged from 337 to 1062, with a pI of 4.62–9.42. A previous report identified three *MAPKKK* genes (*MAP3Kα1*, *MAP3Kβ1*, and *MAP3Kε1*) from *B. napus* (Jouannic *et al.*, 1999), which corresponds to *BnaMAPKKK3*, *-6*, and *-8*, respectively (Supplementary Table S1 available at *JXB* online), and they were added to the analysis. Further comparison of these 31 *BnaMAPKKK* genes with their 31 respective orthologous *AtMAPKKK* genes demonstrated that they show an identity of 14.2–91%, with a similarity of 14.2–91.5% at the nucleotide level. At the protein level, the maximum identity is 95.2% with a minimum of 6.6%, whereas the similarity ranges from 10.5% to 97.7% (Supplementary Table S4 available at *JXB* online). However, a comparison of BnaMAPKKKs with all the 75 OsMAPKKKs showed identity of 6.6–71.7%, with a similarity of 6.6–72.9% at the nucleotide level. At the protein level, the maximum identity is 74.6% with the minimum 3.8%, whereas the similarity ranges from 5.6% to 87.8% (Supplementary Table S4 available at *JXB* online). At this step, it as possible to identify putative orthologues of these *BnaMAPKKK* genes in both *Arabidopsis* and rice using the program InParanoid (Table 1).

**Table 1.** MAPKKK genes identified and cloned from canola

Gene	Subfamily	GenBank accession no.^*a*^	EST count	No. of amino acids	pI value	*Arabidopsis* orthologue^*b*^/AGI no.	Rice orthologue^*b*^ locus
*BnaMAPKKK17*	MEKK	KC190095	2	368	4.92	*AtMAPKKK17/ At2g32510*	OsMAPKKK71/Os02g21700
*BnaMAPKKK18*		KC190096	2	337	5.03	*AtMAPKKK18/ At1g05100*	OsMAPKKK57Os05g46750
*BnaMAPKKK19*		KC190097	1	346	5.02	*AtMAPKKK19/ At5g67080*	OsMAPKKK71/Os02g21700
*BnaMAPKKK20*		KC190098	3	342	4.74	*AtMAPKKK20/ At3g50310*	OsMAPKKK71/Os02g21700
*BnaZIK2*	ZIK	KC190099	11	562	5.73	*AtZIK2/At5g58350*	OsMAPKKK20/Os07g38530
*BnaZIK3*		KC190100	1	567	4.84	*AtZIK3/At3g22420*	OsMAPKKK20/Os07g38530
*BnaZIK4*		KC190101	35	679	4.95	*AtZIK4/At3g04910*	OsMAPKKK20/Os07g38530
*BnaZIK5*		KF129404	26	570	5.08	*AtZIK5/At3g18750*	OsMAPKKK29/OsWNK4/ Os02g45130
*BnaZIK6*		KF129405	17	555	5.3	*AtZIK6/At5g41990*	OsMAPKKK29/Os02g45130
*BnaZIK8*		KC190102	15	312	5.74	*AtZIK8/At5g55560*	OsMAPKKK64/Os07g39520
*BnaZIK9*		KF129406	8	475	4.87	*AtZIK9/At5g28080*	OsMAPKKK20/Os07g38530
*BnaCTR1*	Raf	KF129395	30	803	5.43	*AtCTR1/At5g03730*	OsMAPKKK12/Os09g39320
*BnaRaf17*		KC190103	17	439	7.6	*AtRaf17/At1g14000*	OsMAPKKK65/Os07g43900
*BnaRaf21*		KF129396	51	546	5.57	*AtRaf21/At2g17700*	OsMAPKKK17/Os09g37230
*BnaRaf22*		KF129397	63	409	8.14	*AtRaf22/At2g24360*	OsMAPKKK32/Os08g12750
*BnaRaf23*		KF129398	24	476	9.05	*AtRaf23/At2g31800*	OsMAPKKK74/Os01g66860
*BnaRaf27*		KF129399	16	459	5.85	*AtRaf27/At4g18950*	OsMAPKKK72/Os01g54480
*BnaRaf28*		KC190104	73	410	6.72	*AtRaf28/At4g31170*	OsMAPKKK32/Os08g12750
*BnaRaf29*		KC190105	16	571	5.98	*AtRaf29/At4g35780*	OsMAPKKK17/Os09g37230
*BnaRaf30*		KC190106	22	568	6.49	*AtRaf30 /At4g38470*	OsMAPKKK17/Os09g37230
*BnaRaf33*		KF129400	9	385	7.24	*AtRaf33/At5g50000*	OsMAPKKK34/Os05g50190
*BnaRaf34*		KC190107	2	352	7.92	*AtRaf34/At5g50180*	OsMAPKKK25/Os02g38080
*BnaRaf35*		KC190108	10	1062	5.28	*AtRaf35/At5g57610*	OsMAPKKK35/Os02g54510
*BnaRaf36*		KC190109	2	510	9.42	*AtRaf36/At5g58950*	OsMAPKKK61/Os01g10450
*BnaRaf37*		KF129401	5	398	8.16	*AtRaf37/At5g66710*	OsMAPKKK25/Os02g38080
*BnaRaf39*		KC190110	29	379	8.23	*AtaRaf39/At3g22750*	OsMAPKKK27/Os03g43760
*BnaRaf41*		KF129402	6	356	8.2	*AtRaf41/At3g27560*	OsMAPKKK25/Os02g38080
*BnaRaf46*		KF129403	6	458	9.15	*AtRaf46/At3g59830*	OsMAPKKK74/Os01g66860

^*a*^ The full-length cDNA was cloned and deposited in GenBank.

^*b*^ Putative orthologues were identified by InParanoid (http://inparanoid.sbc.su.se/cgi-bin/index.cgi) with a score of 1 (the maximum score).

*Brassica napus* is an amphidiploid species with an AACC genome (2*n*=38), which is presumably derived from interspecific hybridization of *Brassica rapa* (2*n*=20, AA) and *Brassica oleracea* (2*n*=18, CC) (Rana *et al.*, 2004). Although partial genome sequencing and comparative chromosome painting suggest that ancestral segmental chromosomal duplications led to effective triplication in *Brassica* diploids (Lysak *et al.*, 2005, 2007), various mechanisms of genome evolution have contributed to many situations where fewer than three paralogous genes, corresponding to single orthologues in *Arabidopsis*, are present in the *Brassica* A or C genome (Osborn, 2004; Parkin *et al.*, 2005). *MAPKKK* sequences from the sequenced genome of *B. rapa* were therefore analyzed. As a result, 118 *MAPKKK* genes were identified from *B. rapa* (Supplementary Table S3 available at *JXB* online), which supports that there are not necessarily three paralogous genes existing in the AA genome of *B. rapa* compared with that in *Arabidopsis*. Since the CC genome of *B. oleracea* is not yet known, there is no way to determine the exact number of *MAPKKK* genes in *B. napus*. However, since a double haploid canola variety was used, it should greatly facilitate the identification and functional characterization of *MAPKKK* genes.

### Phylogenetic tree reconstruction and domain analysis of BnaMAPKKK proteins

**Fig. 1.**
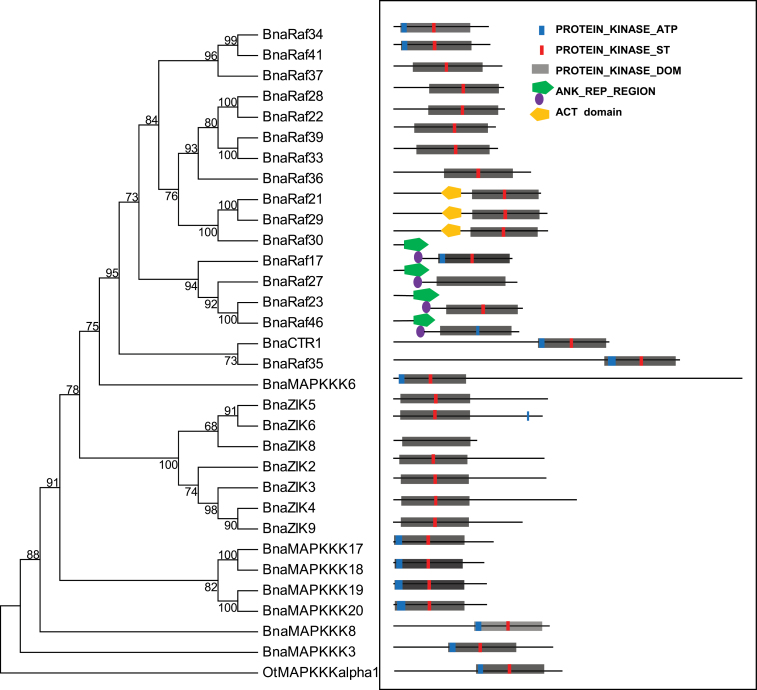
Phylogenetic and motif analysis of MAPKKK proteins. (A) Reconstruction of the phylogenetic tree through the maximum parsimony method. The analysis involved 32 amino acid sequences, with BnaMAPKKKs highlighted in green. There are a total of 205 positions in the final data set. The numbers on the nodes are percentages from a bootstrap analysis of 500 replicates. OtMAPKKK alpha1 is used as the outgroup. (B) Schematic diagram of amino acid motifs of BnaMAPKKKs. Motif analysis was determined by using the Prosite program. The subgroup of BnaMAPKKK and BnaRaf has three protein sites. The grey rectangle is the protein kinase domain. The blue rectangular shape indicates the NP_BIND motif, which is the characteristic sequence of IGKGAYGRV, and, of them, ‘K’ is for ATP binding. The ACT_SITE which is ‘D’, is a proton acceptor highlighted in a red rectangular shape. The ANK_REP_REGION is symbolized by a green pentagon and purple circle. The ACT domain is represented by a yellow pentagon.

To examine the evolutionary relationships of canola MAPKKKs to other representative crops and models, a rooted phylogenetic tree was produced by alignment of full-length amino acid sequences using a maximum parsimony (MP) algorithm. The species used represented the major land plant lineages including several mono- and eudicotyledonous angiosperms, namely: eudicots *A. thaliana* (At), *B. rapa* (Bra), *N. benthamiana* (Nb), and *Solanum lycopersicum* (Sl); monocot *O. sativa* (Os); bryophyte *Physcomitrella patens* (Pp); lycophyte *Selaginella moellendorffii* (Sm), as well as the pico-eukaryotic prasinophyte green alga *Ostreococcus lucimarinus*, which has one of the highest gene densities known in eukaryotes (Lanier *et al.*, 2008). Furthermore, an MAPKKK from a marine green alga *Ostreococcus tauri* (Ot), the world’s smallest free-living eukaryote, has also been identified and was used to root the tree (Fig. 1; Supplementary Fig. S1, Supplementary Table S3 available at *JXB* online). It can be seen that most of the characterized MAPKKKs from other species including NbMAPKKKγ, NbMAPKKKα, LeMAPKKKα, NbMAPKKKε, SlMAPKKKε, OsNK1, and OsCDR1 were clustered within the MEKK subfamily and the others were clustered into the Raf subfamily (Supplementary Fig. S1 available at *JXB* online). Moreover, the presence of a smaller set of MAPKKK members in the green alga *O. lucimarinus* and primitive land plants including *P. patens* and *S. moellendorffii* compared with eudicots and monocots indicated that recent genome duplications may have caused expansion of the *MAPKKK* gene family during the evolution of the angiosperms, which is especially evident within the Raf subfamily (Rao *et al.*, 2010). This is further supported by the fact that the lower plants *S. moellendorffii* and *P. patens* have only 38 and 61 *MAPKKK* genes, respectively (Supplementary Table S3 available at *JXB* online), which is much fewer than found in higher plants such as *Arabidopsis* or rice, which have 80 and 75 members, respectively (Rao *et al.*, 2010).

On the basis of the above phylogenetic analysis, the 31 BnaMAPKKKs could be divided into three major groups, namely groups A(1–4), B(1–4), and C(1–7), which are each supported by significant bootstrap values (Fig. 1; Supplementary Fig. S1 available at *JXB* online). This is in agreement with the conclusion reached with 60 *Arabidopsis* MAPKKKs (Ichimura *et al.*, 2002; Jonak *et al.*, 2002). In total, there were four BnaMAPKKKs in the MEKK subfamily, while there were seven in the ZIK subfamily and 17 in the Raf subfamily among the 28 genes cloned (Fig. 1; Supplementary Fig. S1 available at *JXB* online). It was found that BnaMAPKKK17, -18 -19, and -20 and BnaZIK2, -3, -4, and -8 are in group A, whereas the 17 cloned *BnaRaf* genes are distributed in the B and C groups. For example, BnaRaf35 and BnaCTR1 are in group B, while BnaRaf17, -21, -22, -23, -27, -28, -29, -30, -33, -34, -36, -37, -39, -41, and -46 belong to group C (Fig. 1). It was also observed that in group A, only A1–A4 have been assigned to AtMAPKKKs, as only 60 *AtMAPKKK* genes were used to infer the phylogenetic relationship. The remaining 20 novel *AtMAPKKK* genes, including 11 *AtZIK* genes (from *AtZIK1* to *AtZIK11*) and nine more *AtMAPKKK* genes (from *AtMAPKKK13* to *AtMAPKKK21*) were not included in the previous phylogenetic analysis, nor was any specific subgroup assigned. Based on the analysis presented here, it is proposed to assign *AtMAPKKK13*–*AtMAPKKK21* to subgroup A4, since MAPKKKs within each subgroup were clustered together and were supported by high bootstrapping values (Fig. 1; Supplemtenary Fig. S1 available at *JXB* online). It is noted that orthologous pairs of MAPKKKs between *Arabidopsis* and canola, and between canola and *B. rapa* were clustered in the same subclades of the phylogenetic tree, indicating a higher identity/similarity between them (Fig. 1; Supplementary Fig. S1 available at *JXB* online). Reciprocal Blast analysis and sequence comparison also indicates that the 28 *BnaMAPKKK* genes cloned are paralogous and are not homeologous genes.

Conserved domains and motifs within BnaMAPKKK proteins were further examined. As reported with *Arabidopsis* MAPKKK proteins, the characteristic of the MEKK subfamily in canola includes a conserved kinase domain of G(T/S)Px(W/Y/F)MAPEV. The BnaZIK subfamily has GTPEFMAPE(L/V)Y while the BnaRaf-like subfamily has GTxx(W/Y)MAPE (Supplementary Figs S2–S4 available at *JXB* online) (Jonak *et al.*, 2002). Analysis of the domain architectures of BnaMAPKKKs revealed that most of the 17 BnaRafs have a kinase domain in the C-terminus and a long regulatory domain in the N-terminus. In contrast, eight BnaZIKs have a kinase domain at the N-terminus. As for the four BnaMAPKKKs reported here, the kinase domain is located in the N-terminus (Fig. 1), which is consistent with observations in *Arabidopsis* and rice (Jouannic *et al.*, 1999; Ichimura *et al.*, 2002; Rao *et al.*, 2010). It is proposed that the long regulatory domain in the N-terminus of the Raf subfamily may function to interact with other proteins and hence to regulate or specify their kinase activity (Jouannic *et al.*, 1999).

A careful examination of orthologous *MAPKKK17*, *-18*, *-19*, and *-20* genes between *Arabidopsis* and canola showed that they had a GxGxxS/AxV motif instead of the typical sequence GxGxxGxV in subdomain I, which was also the ATP active site (Supplementary Fig. S2A, B available at *JXB* online). These could be assigned to the A4 subgroups as they existed as an independent subclade within the group A (Supplementary Fig. S1 available at *JXB* online). In addition, the ATP-binding region signature within the kinase domain of BnaMAPKKKs in subgroup A4 was totally different from that of other members in MEKK, Raf, or even the ZIK subfamilies (Supplementary Fig. S2 available at *JXB* online).

The ZIK or WNK subfamily genes, with 10 and nine members in *Arabidopsis* and rice, respectively, are reported to regulate flowering time and circadian rhythms in rice and *Arabidopsis* (Wang *et al.*, 2008; Kumar *et al.*, 2011). However, AtZIK8 was not considered to belong to any of the groups in WNK. The protein kinase ATP-binding region signature of the ZIK subgroup was therefore inspected; this is (L/I)GXG(A/S)(F/V/S)KXVXX(G/A)X(5–18)AVK of Motif 1. The second lysine (K) in domain II was replaced by an asparagine (N) residue, while it is serine (S) in BnaZIK2 (Supplementary Fig. S3A available at *JXB* online), which was consistent with previous findings in rice and *Arabidopsis* (Wang *et al.*, 2008; Kumar *et al.*, 2011). In OsWNK2, -3, -7, and -8, R is present in place of K, while in OsWNK4 and -9, S was present in place of K, and G replaces K in OsWNK6 (Kumar *et al.*, 2011). The missing K residue in domain II in the ZIK subgroup, originally responsible for the coordination of ATP in the active centre, was replaced by a K residue in subdomain I (Xu *et al.*, 2000). It was also noted that there is a 60 amino acid region at the C-terminus of BnaZIKs, which is likely to be the autoinhibitory domain (Supplementary Fig. S3A available at *JXB* online). This is similar to the *Arabidopsis* orthologues (Wang *et al.*, 2008). Furthermore, from the phylogenetic study of ZIKs, it was noted that they could be clustered into four clades or subgroups, with each containing ZIKs from both monocots and eudicots (Supplementary Fig. S3C available at *JXB* online), suggesting that about four ZIK ancestral genes existed before the split of monocots and eudicots. As multiple ZIK genes exist in higher plants such as *Arabidopsis*, rice, and canola, this suggests a relatively recent duplication of *ZIK* genes (Wang *et al.*, 2008). In addition, it was obvious that AtZIKs and BnaZIKs were always clustered together, suggesting a higher similarity of orthologous pairs between *Arabidopsis* and canola, the two representative species of the Brassicaceae family. A previous study identified that plant and animal WNKs form completely different groups, indicating divergence from a common ancestor (Wang *et al.*, 2008). *ZIK*/*WNK* genes do not exist in either yeast or bacterial genomes but exist in some other unicellular eukaryotic genomes such as oomycetes and diplomonads, indicating the origin of early eukaryotes (Wang *et al.*, 2008). Moreover, the monophyletic clade for eudicots indicates the same ancestor before splitting of the monocots and eudicots, while a polyphyletic group may suggest that more than one gene existed before the splitting of the monocots and eudicots (Wang *et al.*, 2008).

From a multiple alignment of the Raf subfamily of canola MAPKKKs, it was inferred that BnaRafs contain a conserved [RK][IV]GXG[SF][FY]G[TE]VX[KRH][GA]X[WF][HFN]G sequence, which is the signature sequence of subdomain I and also discriminates them from the other MAPKKKs (Supplementary Fig. S4 available at *JXB* online) (Jouannic *et al.*, 1999). It was noted that an ACT domain is present only in BnaRaf21, -29, and -30, and this domain is reported to be involved in amino acid metabolism or protein–protein interaction (dimerization) (Feller *et al.*, 2006); however, the significance of it in Raf protein function awaits experimental study. The presence of an ACT domain in some Raf kinases was also observed in rice (Rao *et al.*, 2010). It was also identified that BnaRaf23, -27, and -46 have three ankyrin (ANK) repeats in the N-terminus while BnaRaf17 has only one (Supplementary Fig. S4 available at *JXB* online). The ANK repeat is one of the most common protein–protein interaction motifs and has been found in proteins with diverse functions, such as transcriptional initiators, cell cycle regulators, the cytoskeleton, ion transporters, and signal transducers (Sedgwick and Smerdon, 1999). In *Arabidopsis*, there are 105 predicted ANK repeat-containing proteins, and most of them are transmembrane proteins (Becerra *et al.*, 2004). Since no interactions were detected between BnaRaf23, -27, -46, and eight BnaMKKs (see below), they may interact with some of the unidentified BnaMKKs or other unknown proteins. Another feature of the Raf proteins in plants is the existence of subdomain VIII, which is GTXX(W/Y)MAPE or [LIM]X[SD]X[ST]X[AK]GTP[EQ]W (Jouannic *et al.*, 1999) (Supplementary Fig. S4 available at *JXB* online); however, the conservation of amino acid residues within this subdomain is limited, except at a few sites, as shown by the MEME analysis (Supplementary Fig. S4B available at *JXB* online).

Through the aforementioned phylogenetic analysis, multiple alignments and domain analysis of BnaMAPKKKs in canola, it was concluded that *MAPKKK* genes are ancestral and conserved from lower to higher plants. The classification and function of members of this important gene family may be rather conserved between monocots and dicots. Compared with other species, such as *O. lucimarinus*, *P. patens*, and *S. moellendorffii*, the increasing number of *MAPKKK* genes is probably due to evolutionary events such as genome duplication or expansion. Taken together, during the long history of evolution, the MAPK signalling cascades were relatively conserved, though subfunctionalization or neofunctionalization may have resulted in differences in gene function in specific species.

### Subcellular localization of canola MAPKKK proteins

To investigate further the function of the 28 *BnaMAPKKK* genes cloned, the localization of the encoded proteins was first predicted by using PSORT (http://psort.hgc.jp/), CELLO v2.5 (http://cello.life.nctu.edu.tw), and ESLPred (http://www.imtech.res.in/raghava/eslpred/index.html). It was found that most of the BnaMPKKKs were predicted to be localized to the nucleus, cytoplasm, or plasma membrane, with the exception of BnaRaf17, -28, -29, and BnaZIK2, which were also present in the cytoskeleton and chloroplast, respectively (Supplementary Table S5 available at *JXB* online). For instance, BnaRaf17 was predicted to be localized to chloroplast stroma, microbody (peroxisome), chloroplast thylakoid membrane, and chloroplast thylakoid space, while BnaRaf28 was localized to the endoplasmic reticulum (membrane), plasma membrane, chloroplast thylakoid membrane, and mitochondrial inner membrane. BnaRaf29 was predicted to sit in the cytoplasm, microbody (peroxisome), chloroplast, stroma and chloroplast thylakoid membrane. Transmembrane helices (TMH) of these BnaMAPKKKs were also predicted using TMHMM (http://www.cbs.dtu.dk/services/TMHMM-2.0/); however, no TMH was identified for any of the 28 BnaMAPKKK proteins (data not shown), suggesting that none of these proteins is a transmembrane protein.

**Fig. 2.**
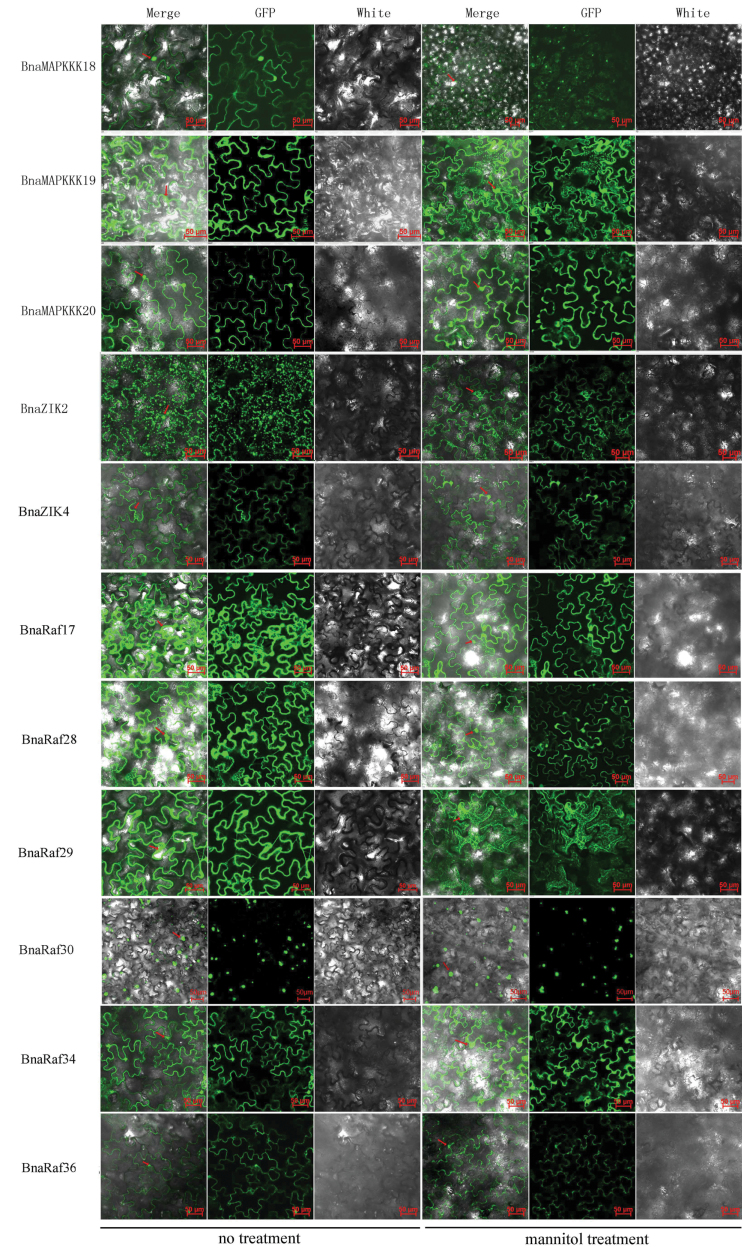
Subcellular localization of BnaMAPKKK proteins in *N. benthamiana* cells using green fluorescent protein (GFP). The six panels represented BnaMAPKKK18, -19, -20, and BnaRaf17, -28, and -29, respectively, under normal and mannitol treatment (500mM for 1h). The red arrows indicate the nuclei or dots of cytosol in the focused cells. In each panel, the extreme left is GFP fluorescence, the middle bright field, and the right an overlay of the two images. Scale bar=50 μm.

To investigate further the subcellular localization of canola MAPKKK proteins, selected genes representing the three subfamilies of BnaMAPKKKs, namely *BnaMAPKKK18*, *-19*, *-20*, *BnaZIK2*, and *-4*, as well as *BnaRaf17*, *-28*, *-29*, -*30*, *-34*, and *-36* were expressed as fusion proteins with GFP in *N. benthamiana*. The recombinant plasmids were transformed into agrobacteria and then infiltrated into the lower epidermal leaves of *N. benthamiana*, with the GFP signals observed 2 d later. It was found that in leaf cells harbouring the fusion proteins of BnaMAPKKK18, -19, and -20, BnaZIK2 and -4, and BnaRaf17, -28, -29, -30, -34, and -36, the GFP signals were present in the cytoplasm and nucleus (Fig. 2). For BnaZIK2, the signals were observed in the chloroplast as well as in the cytoplasm and nucleus, while for BnaRaf30, the GFP signals were only present in nuclei. As a control, the subcellular localization of the GFP protein was also examined in tobacco leaf cells, and green signals were obviously present in both the cytosol and nuclei (data not shown). Leaf discs from the agroinfiltrated plants were further treated with a high osmotic solution (500mM mannitol) for 1h, and the GFP signals were observed again under the same confocal settings. As shown, most of the BnaMAPKKKs tested were still localized in both the cytoplasm and nuclei, except BnaRaf30 (Fig. 2). The *in planta* demonstration of the subcellular localizations of BnaMAPKKKs is expected to reflect more accurately the natural subcellular localization of these proteins, as compared with the *in silico* predictions.

### Identification and validation of BnaMAPKKK and BnaMKK interactions

To explore the function of *MAPKKK* genes in canola, it is essential to identify their direct MKK targets. Therefore, a Y2H assay was performed to identify the BnaMKKs interacting with each of the 28 BnaMAPKKKs. To this end, the coding regions of 28 *BnaMAPKKK* and eight *BnaMAPKK* genes were first cloned into the GAL4-BD bait and GAL4-AD prey vectors, respectively. The autoactivation activity of each of the 28 BnaMAPKKK proteins in yeast was then tested, and it was found that BnaMAPKKK18, BnaZIK3, BnaZIK4, and BnaRaf35 showed evident autoactivation activity (data not shown). To solve this issue, the coding regions of *BnaMAPKKK18*, *BnaZIK3*, *BnaZIK4*, and *BnaRaf35* were fused in-frame with the GAL4-AD domain whereas those of the eight *BnaMKK* genes were fused with the GAL4-BD domain. Thirdly, individual BnaMAPKKK–BnaMKK pairs were co-transformed into yeast cells, with colonies tested on both selective media, followed by titration and β-galactosidase activity assay to examine the strength and genuineness of interactions. As a result, 22 pairs of interactions were identified, and six of the BnaMKK proteins were found to interact with at least one BnaMAPKKK protein in the Y2H assay, while BnaMKK1 and BnaMKK4 did not interact with any of the 28 BnaMAPKKK proteins assayed (Table 2; Supplementary Fig. S5 available at *JXB* online), suggesting that these two MKK proteins may be transducer signals from other upstream MAPKKK proteins. There were six BnaMAPKKKs, BnaMAPKKK17, -18, and -20, BnaZIK3 and -4, and BnaRaf35, that interacted with BnaMKK3; six BnaMAPKKKs, BnaMAPKKK17, -19, -20, BnaZIK2, -9, and BnaRaf28, with BnaMKK9; three BnaMAPKKKs, BnaZIK2, -9, and BnaRaf28, with BnaMKK5; and five BnaMAPKKKs, BnaMAPKKK17, -19, -20, BnaZIK2, and BnaRaf28 with BnaMKK8. As regards BnaMKK2 and -6, only BnaRaf28 interacted with both of them (Table 2).

**Table 2.** Summary of yeast two-hybrid assay of interactions between BnaMAPKKK and BnaMKK proteins

pGBKT7 pGADT7	BnaMKK1	BnaMKK2	BnaMKK3	BnaMKK4	BnaMKK5	BnaMKK6	BnaMKK8	BnaMKK9
BnaMAPKKK17	–	–	++BiFC	–	–	–	++	+BiFC
BnaMAPKKK18	–	–	++	–	–	–	–	–
BnaMAPKKK19	–	–	–	–	–	–	++	+BiFC
BnaMAPKKK20	–	–	++BiFC	–	–	–	++	+BiFC
BnaZIK2	–	–	–	–	+ BiFC	–	++	+BiFC
BnaZIK3	–	–	+	–	–	–	–	–
BnaZIK4	–	–	++BiFC	–	–	–	–	–
BnaZIK5	–	–	–	–	–	–	–	–
BnaZIK6	–	–	–	–	–	–	–	–
BnaZIK8	–	–	–	–	–	–	–	–
BnaZIK9	–	–	–	–	++	–	–	++
BnaCTR1	–	–	–	–	–	–	–	–
BnaRaf17	–	–	–	–	–	–	–	–
BnaRaf21	–	–	–	–	–	–	–	–
BnaRaf22	–	–	–	–	–	–	–	–
BnaRaf23	–	–	–	–	–	–	–	–
BnaRaf27	–	–	–	–	–	–	–	–
BnaRaf28	–	++ BiFC	–	–	+ BiFC	+ BiFC	++	++ BiFC
BnaRaf29	–	–	–	–	–	–	–	–
BnaRaf30	–	–	–	–	–	–	–	–
BnaRaf33	–	–	–	–	–	–	–	–
BnaRaf34	–	–	–	–	–	–	–	–
BnaRaf35	–	–	++	–	–	–	–	–
BnaRaf36	–	–	–	–	–	–	–	–
BnaRaf37	–	–	–	–	–	–	–	–
BnaRaf39	–	–	–	–	–	–	–	–
BnaRaf41	–	–	–	–	–	–	–	–
BnaRaf46	–	–	–	–	–	–	–	–

The interaction strength was scored visually, from no interaction (–) to strong interaction (++).

BiFC indicates interactions confirmed through bimolecular fluorescence complementation *in planta.*

Parts of the protein–protein interactions identified in the Y2H assays were confirmed using the BiFC procedure in plant cells. The presence of yellow fluorescence signals showed that BnaMAPKKK17, -19, -20, BnaZIK2, -9, and BnaRaf28 interacted with BnaMKK9; BnaRaf28 with BnaMKK6; BnaZIK2 and BnaRaf28 with BnaMKK5; BnaMAPKKK17, -20, and BnaZIK4 with BnaMKK3; and BnaRaf28 with BnaMKK2 in the epidermal cells of *N. benthamiana* (Supplementary Fig. S6 available at *JXB* online). The respective negative controls (target proteins fused to half of YFP co-expressed with the other half of the YFP) did not yield detectable YFP signals (Supplementary Fig. S6 available at *JXB* online). Taken together, the identification of BnaMAPKKK–BnaMKK pairs provided useful information to dissect their function in canola response to abiotic and biotic stresses.

Since MAPKKKs usually exert their function through phosphorylation of downstream MKK proteins, co-localization of interacting MAPKK and MKK proteins is normally expected for signal transduction. Therefore, the *in vivo* localizations of pairs of interacting BnaMAPKKKs and BnaMKKs was compared, including Raf28–MKK2, MAPKKK18–MKK3, MAPKKK20–MKK3, and ZIK4–MKK3. The subcellular localization of MKK2 and MKK3 was previously shown to be the cytoplasm and nucleus (Liang *et al.*, 2013). The co-localization of MKK2 and MKK3 with BnaMAPKKKs probably facilitates their interactions in the cytoplasm as well as the nuclei (Fig. 2; Supplementary Fig. S6 available at *JXB* online). Hence, the co-localization of BnaMAPKKK–BnaMKK interaction pairs facilitates their interaction and phosphorylation to mediate timely responses to external and internal stimuli. However, it should be noted that even if two proteins are identified to interact in a Y2H assay or after co-expression in plants, they may not necessarily do so under natural conditions, as a result of differences in spatiotemporal expression patterns.

### Expression analysis of *BnaMAPKKK* genes in response to stress treatments

**Fig. 3.**
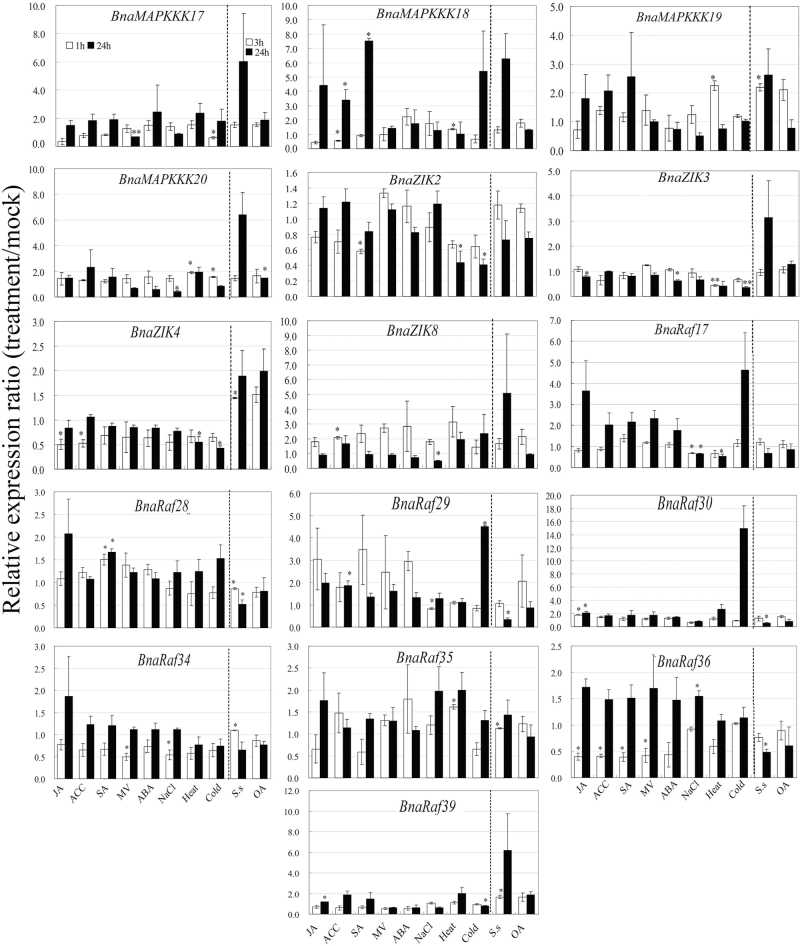
Expression analyses of *BnaMAPKKK* genes in response to various treatments, including 20 μM JA, 1mM ACC, 2mM SA, 10 μM paraquat (MV), 50 μM ABA, 200mM NaCl, heat (37 °C), cold (4 °C), *S. sclerotiorum* infection, and 5mM oxalic acid (OA). Data are the mean of three biological replicates ±SE. Asterisks denote significant differences (compared with 1) by Student *t*-test analysis (*P≤0.05; **P≤0.01).

To elucidate of the functions of *BnaMAPKKK* genes in the context of abiotic and biotic stresses, the expression patterns of 16 selected *BnaMAPKKK* genes, including four *MAPKKK* genes, four *ZIK* genes, and eight *Raf* genes, were studied using qRT–PCR. Among the stress treatments applied, ABA is a well-known abiotic stress hormone, and JA, ethylene, and SA are well-known defence hormones, while MV can trigger ROS burst in plant cells. OA, on the other hand, is a pathogenicity factor produced by *S. sclerotiorum*, and can elicit PCD and suppress the oxidative burst of host plants (Cessna *et al.*, 2000; Guimaraes and Stotz, 2004; Kim *et al.*, 2008). Canola seedlings were subjected to moderate stress treatments at two time points to better monitor the transcript changes of the investigated *BnaMAPKKK* genes. Data of three independent biological replicates were subjected to statistical analysis to identify *BnaMAPKKK* genes responsive to one or a combination of stress conditions (Fig. 3; Supplementary Fig. S7A available at *JXB* online). It was found that JA modulated expression of five genes, among which *BnaRaf30* was induced while *BnaZIK3*, *BnaZIK4*, *BnaRaf36*, and *BnaRaf39* were repressed at one or both time points tested. Five genes, *BnaMAPKKK18*, *BnaZIK3*, *BnaZIK8*, *BnaRaf29*, and *BnaRaf36*, were responsive to ACC, among which *BnaZIK8* and *BnaRaf29* were up-regulated while *BnaMAPKKK18*, *BnaZIK3*, and *BnaRaf36* were down-regulated. SA significantly induced *BnaMAPKKK18* and *BnaRaf28* while it repressed *BnaZIK2*, *BnaRaf34*, and *BnaRaf36* at specific time points. Expression of *BnaMAPKKK17*, *BnaRaf34*, and *BnaRaf36* was down-regulated by MV treatment at one time point tested. ABA seemed only to repress *BnaZIK3* expression and exerted no effect on the transcription of the other genes. On the other hand, salinity induced *BnaMAPKKK20* and *BnaRaf36* at the 1h and 24h time points, respectively, whereas it down-regulated *BnaMAPKKK20* (24h), *BnaZIK3*, *BnaZIK8*, and *BnaRaf17* (1h and 24h), as well as *BnaRaf29* and *BnaRaf34* (1h). Seven genes were affected by heat treatment, among which the transcript levels of *BnaMAPKKK18*, *-19*, and *BnaRaf35* were increased at the 1h time point, while those of *BnaZIK2*, *-3*, *-4*, and *BanRaf17* were decreased at 24h or at both time points by heat stress. In addition, moderate cold stress increased the transcript abundance of *BnaMAPKKK20* and *BnaRaf29* at the 1h time point, but decreased that of *BnaMAPKKK17*, *ZIK2*, *-3*, *-4*, and *BanRaf39* mostly at the 24h time point. Upon challenge by the fungal pathogen *S. sclerotiorum*, *BnaMAPKKK19*, *ZIK4*, *Raf34*, *-35*, and -*39* were significantly induced at the 3h time point, while *BnaRaf28*, *-29*, *-30*, and *-36* were repressed at the 24h time point. Lastly, only *BnaMAPKKK20* was induced by OA treatment (Fig. 3; Supplementary Fig. S7A available at *JXB* online), although to a rather small extent. Taken together, these data indicate that on one hand, some *BnaMAPKKK* genes participate in transduction of multiple stresses; and, on the other hand, a specific stress activates transcription of more than one *BnaMAPKKK* gene, providing evidence that BnaMAPKKK plays a role in the cross-talk of multiple stresses, including both abiotic and biotic stresses.

Since public data on *Arabidopsis* gene expression are available and *Arabidopsis* is a close relative of canola, comparative analysis of orthologous *MAPKKK* genes between these two species was performed to examine to what extent the behaviours of *AtMAPKKK* and *BnaMAPKKK* genes in response to stress are correlated. To this end, expression profiles of *AtMAPKKK* genes in response to different hormone and stress treatments were investigated using ATH1 GeneChip data. It should be noted that the stress conditions applied to canola seedlings for the qRT–PCR assay are very similar to those for the *Arabidopsis* data set. As shown in Supplementay Fig. S7B available at *JXB* online, *AtAMPKKK17* was induced in seedlings 3h after treatment with 10 μM ABA and 6h after heat (38 °C) treatment. *AtMAPKKK18* was up-regulated by 10 μM ABA at 1h and 3h after treatment. *AtMPKKK19* was induced by *B. cinerea*, 10 μM MeJA at 30min, 1h, and 3h, and 10 μM SA, while it was repressed after treatment with 150mM NaCl. *Botrytis cinerea* is a grey mould fungus and it is taxonomically closely related to the white mould fungus *S. sclerotiorum* (Amselem *et al.*, 2011). Both pathogens are necrotrophic by producing similar virulence factors such as OA, cell wall-degrading enzymes, etc. (Gentile, 1954; Choquer *et al.*, 2007; Kim *et al.*, 2008; Amselem *et al.*, 2011). *AtMAPKKK20* was up-regulated by 10 μM MeJA after 1h treatment. It was also identified that both *AtMAPKKK17* and *-18* are also induced in the root samples after treatment with 150mM NaCl for 30min to 24h. *MAPKKK19* orthologues in both canola and *Arabidopsis* are induced by necrotrophic pathogens (Fig. 3; Supplementary Fig. S7 available at *JXB* online). However, differences in the expression profiles of BnaMAPKKK and AtMAPKKK after some treatments are also evident (Supplementary Fig. S7 available at *JXB* online). For instance, *AtZIK4* was repressed by SA and heat, whereas it was induced by the biotrophic bacterial pathogen *P. syringae*. *BnaZIK4* was also repressed by JA, heat, cold, and ACC, but was induced by *S. sclerotiorum* (Fig. 3; Supplementary Fig. S7A available at *JXB* online). *AtZIK8* was induced by *B. cinerea*, SA, and drought (Supplementary Fig. S7B available at *JXB* online), whereas *BnaZIK8* was induced by ACC only (Fig. 3). *AtRaf17* was induced by SA treatment, whereas *BnaRaf17* was repressed by NaCl and heat. *AtRaf18* was induced by SA while it was repressed by salt treatment (Supplementary Fig. S7 available at *JXB* online). *AtRaf29* was induced by ABA; *AtRaf30* was induced by MeJA, SA, and cold, while it was repressed by salt. *AtRaf34* was repressed by cold and heat, and *AtRaf35* was induced by ABA, heat, and salt treatments (Supplementary Fig. S7B available at *JXB* online).

In summary, comparison of transcript expression profiles of some presumed orthologous *MAPKKK* genes in *Arabidopsis* and canola show similarities in response to abiotic stress, hormone, and pathogen treatments. For instance, *Raf28* was induced by SA, *Raf30* was induced by JA, and *MAPKKK19* was induced by necrotrophic fungi. However, significant differences were also observed between these two species and this may be attributed to evolutionary differences in gene expression that have occurred, and also experimental differences, especially sampling time.

### Characterization of *BnaMAPKKK* gene functions in cell death

**Fig. 4.**
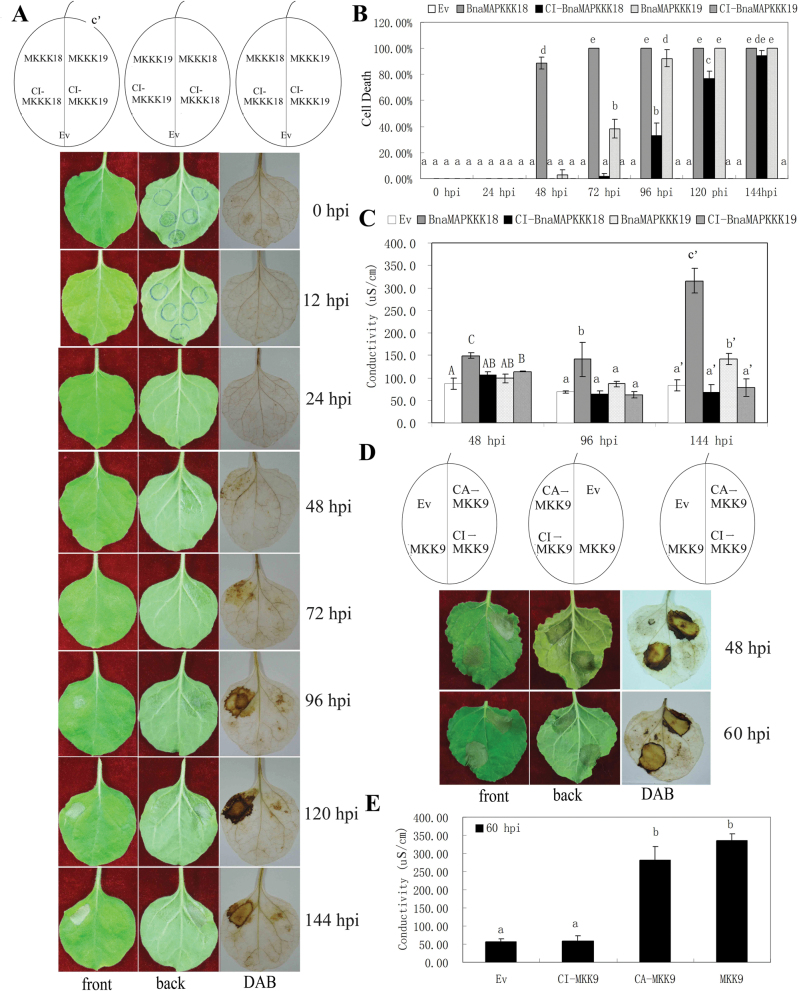
Overexpression of *BnaMAPKKK18*, *-19*, and *MKK9* induced pathogen-independent cell death in *N. benthamiana* leaves. Leaves were infiltrated with agrobacteria carrying wild-type genes or mutated versions. All experiments were performed three times with similar results obtained. (A) Symptoms of *N. benthamiana* leaf areas expressing *BnaMAPKKK18* and *BnaMAPKKK19* genes and their mutated forms 24, 48, 72, 96, 120 and 144h post-infiltration (hpi). The left, middle, and right panels represent the front and back, and DAB staining, respectively. (B) Quantification of cell death in *N. benthamiana* leaves expressing *BnaMAPKKK18* or *BnaMAPKKK19* and their mutated derivates by examining the percentages of leaf sites with water-soaked symptoms at various time points. (C) Measurement of electrolyte leakage in leaf discs expressing *BnaMAPKKK18* or *BnaMAPKKK19* and their mutated derivates at 48, 96, and 144 hpi. (D) Symptoms of *N. benthamiana* leaf areas expressing *BnaMKK9* or its mutated versions at 48 and 60 hpi. (E) Measurement of electrolyte leakage in leaf discs transiently expressing *BnaMKK9* and its mutated derivates at 60 hpi. Values represent the means of three independent assays for each time point ±SE. Identical and different letters represent non-significant and significant differences (*P*≤0.05), respectively.

In the above Y2H and qRT–PCR studies, certain *BnaMAPKKK* genes were identified whose encoded proteins interacted with specific BnaMKKs, and that also increased in transcript abundance in response to multiple stress conditions. To explore their functions further, it was selected to express multiple genes transiently, including *BnaMAPKKK18*, *-19*, and *BnaMKK9* as well as their constitutively inactive (CI) mutant forms individually under the CaMV 35S promoter through agroinfiltration into *N. benthamiana* leaves. The inactive form of the MAPKKKs or MKKs was achieved by replacing the ATP-binding site (lysine, K) with an arginine (R) or with a methionine (M) (Melech-Bonfil and Sessa, 2010; Hashimoto *et al.*, 2012). Interestingly, ectopic expression of either *BnaMAPKKK18* or -*19* caused pathogen-independent cell death compared with the CI version of their respective genes or empty vector control, beginning 48h or 72h post-infiltration (hpi), and this lasted till 144 hpi (Fig. 4A). It was also observed that the symptom of a water-soaked area became apparent as early as 24 hpi (Fig. 4A). To explore the role of hydrogen peroxide (H_2_O_2_) during cell death, DAB staining was performed and the results showed that there was strong staining in sites expressing only *BnaMAPKKK18* or *-19* beginning at 48 hpi and continued till 144 hpi, but not in any control sites (Fig. 4A, B). Nitroblue tetrazolium (NBT) staining of superoxide showed similar changes (data not shown). Moreover, the electrolyte leakage of leaf discs taken from leaves expressing *BnaMAPKKK18*, *BnaMAPKKK18* ^*K32G*^, *BnaMAPKKK19*, *BnaMAPKKK19* ^*K37G*^, and the empty vector was examined. The results showed that a significant increase in ion leakage was visible 3 d after agroinfiltration of *BnaMAPKKK18* or *-19*, in contrast to that of leaves expressing *BnaMAPKKK18* ^*K32R*^ or the control plasmid (Fig. 4C), which further demonstrates that the hypersensitive response (HR)-like cell death associated with hydrogen peroxide production is triggered by high expression of *BnaMAPKKK18* and *-19*. From the phylogenetic analysis, *BnaMAPKKK18* and *-19* were classified into subgroup A4 (Supplementary Fig. S1 available at *JXB* online). Interestingly, a literature search and phylogenetic analysis identified that SlMAPKKKε and NbMAPKKKε in the A4 group positively regulate cell death signalling in plant immunity (Supplementary Fig. S1 available at *JXB* online) (Melech-Bonfil and Sessa, 2010; Hashimoto *et al.*, 2012). Epistasis experiments showed that SlMAPKKKε-mediated cell death is negatively regulated by SIPKK (Melech-Bonfil and Sessa, 2010). The results therefore implied that BnaMAPKKK18 and -19 are two novel kinases mediating cell death in plants.

From our Y2H results, BnaMAPKKK18 interacted with BnaMKK3. However, expression of *BnaMKK3* in *N. benthamiana* did not induce significant cell death (data not shown). Though the function of *BnaMKK3* has not been identified yet, its orthologue *AtMKK3* is known to regulate the JA signal transduction pathway together with *AtMPK6* (Takahashi *et al.*, 2007) and also plays an important role in plant immune and stress responses possibly through interacting with group C MPKs, including MPK1, -2, -7, and -14, and the downstream pathogenesis-related 1 (PR1) (Doczi *et al.*, 2007).

In the above Y2H assay, it was found that BnaMAPKKK19 interacted with BnaMKK9. Interestingly, it was also found that BnaMKK9 also induced cell death when transiently expressed in *N. benthamiana* leaves (Fig. 4D). Interestingly, expression of the constitutively active BnaMKK9 (CA-MKK9) also induced strong cell death, whereas the CI BnaMKK9 (CI-MKK9) or empty plasmid did not (Fig. 4D). DAB and NBT staining demonstrated that there was evident accumulation of ROS at sites expressing *BnaMKK9* or its constitutive form, but not in the sites expressing the inactive form or empty vector (Fig. 4D). To quantity further the extent of cell death, electrolyte leakages of leaf discs taken from leaves expressing the wild-type or mutant form of the *BnaMKK9* gene and the empty vector were monitored. The results showed that a significant increase in ion leakage was visible 3 d after agroinfiltration of canola *MKK9* or *CA-MKK9* in contrast to that of leaves expressing *CI-MKK9* or the control plasmid (Fig. 4E). A literature search indicated that *Arabidopsis* MKK9 can similarly accelerate cell death in *N. benthamiana* through Sgt1, a known regulator of cell death (Popescu *et al.*, 2009), which suggests that orthologous *MKK9* between canola and *Arabidopsis* may have a conserved function.

To understand further the role of the MAPKKK–MKK signaling cascade in cell death and ROS accumulation, co-overexpression analysis of *BnaMAPKKK19* and *BnaMKK9* in *N. benthamiana* was performed, considering the fact that BnaMAPKKK19 and BnaMKK9 interacted in both Y2H and BiFC. As shown in Supplementary Fig. S8 available at *JXB* online, BnaMKK9 alone is already active, and co-expression of *MKK9* and *MAPKKK19* does not have a significant additive or synergistic effect, as shown from statistical analysis of the cell death index and electrolyte leakage (Supplementary Fig. S8B, C available at *JXB* online). Meanwhile, cell death induced by expression of *BnaMKK9* was not significantly influenced in the absence of upstream *BnaMAPKKK19*, neither was *BnaMAPKKK19* in the absence of downstream *BnaMKK9*. It would be interesting to identify the substrates of MAPKKK19 and MAPKKK18 in *N. benthamiana,* and to explore the relationship between these two MAPKKKs and ROS-generating enzymes Rbohs (respiratory burst oxidase homologues) (Torres and Dangl, 2005). Moreover, whether the other cloned *BnaMAPKKK* and *BnaMKK* genes could regulate cell death and ROS signalling is still under investigation in our lab.

A complete module, which includes BnaMKK9–BnaMPK1/2–BnaWRKY53 was previously identified (Liang *et al.*, 2013), which links the BnaMKK9–MPK1/2 cascade to downstream WRKY transcription factor(s), and they may together regulate cell death and/or leaf senescence, since AtWRKY53 was identified to regulate leaf senescence (Miao *et al.*, 2007). Whether BnaMAPKKK19 mediates cell death through MKK9–BnaMAPK1/2–BnaWRKY53 and how the ethylene signalling pathway is integrated by this module need to be further elucidated. Besides BnaMAPKKK19, four more BnaMAPKKKs, namely BnaMAPKKK17, -20, BnaZIK2, and BnaRaf28, were also identified to interact with BnaMKK9 (Table 2; Supplementary Figs S5, S6 available at *JXB* online); however, none of these BnaMAPKKKs was shown to elicit cell death (data not shown), indicating that they are possibly involved in other biological processes.

The activation of AtMKK5 can lead to HR-like cell death through ethylene signalling perception (Liu *et al.*, 2008). *Arabidopsis* MKK4 and MKK5 belong to group C MKKs with D sites K/R-K/R-X(1–5)-L/I-X-L/I at the N-termini (Bardwell *et al.*, 2001; Grewal *et al.*, 2006). More recently, it is reported that the D site in SlMKK2 of *S. lycopersicum* is critical for interacting with SlMPK3 and triggering PCD (Oh *et al.*, 2013). Though *Arabidopsis* YODA–MKK4/MKK5–MPK3/MPK6 modules regulate stomatal development and patterning (Wang *et al.*, 2007), the direct upstream component(s) of AtMKK5 has not been reported yet. In the Y2H assay, two BnaMAPKKKs, namely BnaZIK2 and BnaRaf28, interacted with BnaMKK5 (Table 2; Supplementary Fig. S5 available at *JXB* online), and the interactions were confirmed through BiFC (Supplementary Fig. S6 available at *JXB* online). However, no cell death induced by *BnaZIK2* and *BnaRaf28* overexpression in *N. benthamiana* was observed (data not shown). Whether there are other canola *MAPKKK* genes mediating cell death or ROS signalling awaits further investigation. Also, whether and how BnaMAPKKK18 and -19 modulate plant immunity against fungal pathogens such as *S. sclerotiorum* needs to be experimentally determined.

### Conclusion

**Fig. 5.**
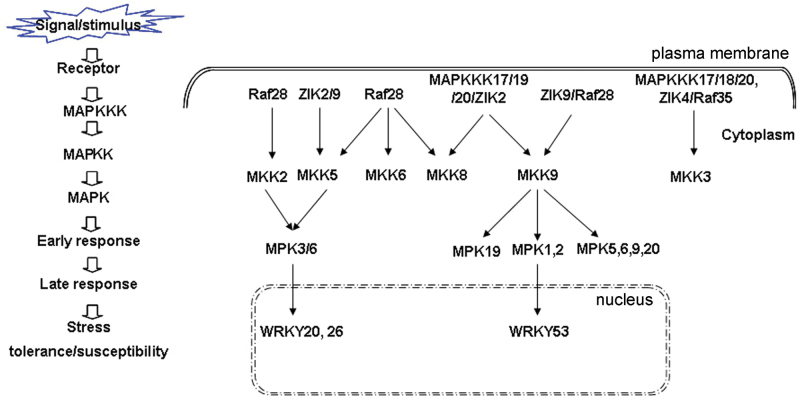
A proposed model summarizing MAPK cascades in canola. Dotted lines indicate the possibility of the MAPK cascades acting either in a parallel or a sequentially co-dependent manner. Solid lines indicate confirmed interactions and dashed lines with arrows mean possible regulation at the transcriptional level.

MAPK signalling pathways are very important in plant development, abiotic stress, and defence responses. So far, the function of only a few *MAPKKK* genes in *Arabidopsis*, rice, tomato, and tobacco have been reported (Rodriguez *et al.*, 2010; Meng and Zhang, 2013). In the present study, the identification of the *MAPKKK* gene family in the important oilseed crop, canola, was described. A total of 28 novel *MAPKKK* genes in canola were cloned and characterized, and two novel *MAPKKK* genes, *BnaMAPKKK18* and *-19*, mediating cell death were identified. A few complete MAPK modules were identified through linking MAPKKKs to recently identified downstream components (Liang *et al.*, 2013). For example, BnaZIK2/BnaRaf28–BnaMKK5–BnaMPK3/6–BnaWRKY20/26, BnaRaf28–BnaMKK2/5–BnaMPK3/6–BnaWRKY20/26, and BnaMAPKKK17/19/20/ZIK2/BnaRaf28–BnaMKK9–BnaMPK5/-9/-19/-20 (Fig. 5). Many of these have not been reported even in *Arabidopsis*, indicating that different combinations of BnaMAPKKK–BnaMKK–BnaMPKs are very likely to be involved in the responses to different external and internal stimuli in canola. In the present and previous studies, it was found that there are differences in expression patterns of orthologous *MAPKKK*, *MKK*, and *MPK* genes in response to different stimuli between *Arabidopsis* and canola, and it was also identified that even the interaction pairs are not conserved between these two species, which highlights the limitations of applying conclusions from the model species *Arabidopsis* to canola (Liang *et al.*, 2013).

In a previous study, canola *MAPKKK17*, *MAPKKK18*, as well as *MPK3*, *MPK4*, *MPK6*, and *MPK17* were identified as being responsive to *S. sclerotiorum* infection (Yang *et al.*, 2007). Seven *MKK* and 12 *MPK* genes from canola were recently identified, and their function was analysed in the context of abiotic and biotic stresses (Liang *et al.*, 2013). Here the identification and characterization of their upstream regulators, MAPKKKs, were described. Although the present 28 *BnaMAPKKK* genes comprise only 44% of the 66 identified genes for canola, 15 BnaMAPKKK–BnaMKK interaction pairs were identified, which provide clues to dissect the function of individual BnaMAPKKKs in canola. For instance, BnaRaf28 could interact with BnaMKK2, -5, -6, -8, and -9, while six different BnaMAPKKKs could interact with BnaMKK9, and six BnaMAPKKKs interacted with BnaMKK3 (Table 2; Fig. 5). It is proposed that the different combinations of BnaMKKK–BnaMKK pairs may fulfil their function in response to diverse abiotic stress signalling pathways and plant immune responses. However, the possibility cannot be excluded that even if two proteins are found to interact in Y2H assay or after co-expression in *N. benthamiana*, they may not necessarily do that in the natural situation. Moreover, it was identified that two novel *MAPKKK* genes, *BnaMAPKKK18* and *-19*, could induce cell death when transiently expressed in leaves of *N. benthamiana*, possibly through accumulation of ROS. Moreover, BnaMKK9 and its constitutively active form also induced strong cell death and H_2_O_2_ accumulation. However, a more complete picture of *MAPKKK* genes and the related signalling cascade in canola will not be available before the remaining *MAPKKK* genes are cloned from canola and characterized. Overall, the present study of *MAPKKK* genes in canola lays the foundation for further exploration of their roles in abiotic stress signalling and in plant immunity against *S. sclerotiorum*. It also provides important information for genetially manipulating the abundance and/or activity of related MAPKKKs and their components in the MAPK pathway to improve stress tolerance of canola.

## Supplementary data

Supplementary data are available at *JXB* online.

Figure S1. Phylogenetic analysis of MAPKKKs from representative species.

Figure S2. Multiple alignment of MEKK subfamily MAPKKK proteins in representative species.

Figure S3. Multiple alignment of ZIK subfamily MAPKKK proteins in representative species.

Figure S4. Multiple alignment of Raf subfamily MAPKKK proteins in representative species.

Figure S5. Yeast two-hybrid (Y2H) assay of interactions between MAPKKK and MKK proteins in canola.

Figure S6. Analysis of BnaMAPKKK and BnaMKK interactions in *N. benthamiana* through bimolecular fluorescence complementation (BiFC).

Figure S7. The heat maps of the expression profiles of MAPKKK genes of canola and *Arabidopsis* in responses to abiotic and biotic stresses.

Figure S8. Co-expression analysis of *BnaMAPKKK19* and *MKK9* in eliciting pathogen-independent cell death in *N. benthamiana* leaves.

Table S1. BnaMAPKKK EST summary.

Table S2. Primers used in this study.

Table S3. MAPKKK sequences from different species used for phylogenetic analysis.

Table S4. Similarity and identity analysis of MAPKKK genes/proteins between *Arabidopsis*, rice, and canola.

Table S5. Computational prediction of subcellular localizations of MAPKKK proteins in canola.

Supplementary Data
