# Supplementary material for: Identification and functional analysis of mitogen-activated protein kinase kinase kinase (MAPKKK) genes in canola (Brassica napus L.)
Source: J Exp Bot. 2014 Mar 6;65(8):2171–88. doi: 10.1093/jxb/eru092 (PMC3991747; doi:10.1093/jxb/eru092)
Supplement: Supplementary Data [file supp_65_8_2171__index.html]

Identification and functional analysis of mitogen-activated protein kinase kinase kinase (MAPKKK) genes in canola (Brassica napus L.) — Identification and functional analysis of mitogen-activated protein kinase kinase kinase (MAPKKK) genes in canola (Brassica napus L.) — Supplementary Data 

# Identification and functional analysis of mitogen-activated protein kinase kinase kinase (MAPKKK) genes in canola (*Brassica napus* L.)

## Supplementary Data

Data files

**Files in this Data Supplement:**

- Supplementary Data - Supplementary Data
