# Supplementary material for: Identification and functional analysis of mitogen-activated protein kinase kinase kinase (MAPKKK) genes in canola (Brassica napus L.)
Source: J Exp Bot. 2014 Mar 6;65(8):2171–88. doi: 10.1093/jxb/eru092 (PMC3991747; doi:10.1093/jxb/eru092)
Supplement: Supplementary Data [file supp_eru092_jexbot111013_file001.pdf]

Identification and functional analysis of mitogen-activated protein kinase kinase kinase (MAPKKK) genes in canola (*Brassica napus* L.)

Yun Sun, Chen Wang, Bo Yang, Feifei Wu, Xueyu Hao,Wanwan Liang, Fangfang Niu, Jingli Yan, Hanfeng Zhang, Boya Wang, Michael K Deyholos, Yuan-Qing Jiang

Table S1. BnaMAPKKK EST summary.

Gene	GenBank accession numbers of ESTs	No.of contigs	No.of singletons	total No.of ESTs
BnaMAPKKK1	DY022877,EV008430,GR446403,EV149603,EV149926, EV149087,EV149217	2	1	7
BnaMAPKKK2	CX192261	0	1	1
BnaMAPKKK3 (BnMAP3Kα1)	CX191632,EV172297,GT082081,EE542547,EE550677	2	0	5
BnaMAPKKK4	EV072989,EE444225,ES910539,CX187618,ES919039	2	1	5
BnaMAPKKK5	GT078796,GT078644,EV217621,EV158579	2	0	4
BnaMAPKKK6 (BnMAP3Kε1)	EV145804,EV054446,EV055798	1	1	3
BnaMAPKKK7	CD818725,EE403405,DY002368,EL629803,EE457278,ES979963,ES966150,EV042545,EV018421,EV078443,EL626620,EL627580	2	3	12
BnaMAPKKK8 (BnMAP3Kβ1)	EV192042,EV035080,FG566093,EV055951,CJ999505,EV023028	2	1	6
BnaMAPKKK9/MEKK2	EV161695,EV161322,EV161456,EV161613,EV161538	2	0	5
BnaMAPKKK11	EE429793,DY000072,ES266880,EV117746,FG553806,EL591134	1	3	6
BnaMAPKKK12	EE452764,EV008430,GR446403	1	1	3
BnaMAPKKK13	ES909856,ES913159,FG564143,FG557695	1	0	4
BnaMAPKKK14	EE477468,GR443470,CN728075,EV076377,EV078743,CX194918,EE461225,GR439026	2	1	8
BnaMAPKKK15	EV111390,EV111105	1	0	2
BnaMAPKKK17	DK466745,DK524925	1	0	2
BnaMAPKKK18	GR719871,GR727308	1	0	2
BnaMAPKKK19	CX279903	0	1	1
BnaMAPKKK20	EE444892,EE417560 ,GR436387	1	0	3
BnaZIK1	CD828824,EV027260,EV136051,ES905716,EV028082,EE432777,FG561292,EV190872,EV195091,CD836798,GT084453,GR462753,EE444646,EV134286,EV133959,EV006837,EE471093,ES905420,ES989300,EV184525,EV183080,CD835586,CD835289,GR463177,CD832636	6	0	25
BnaZIK2	ES911192,EV052919,EE565337,EV222224,EV221813,EV142598,EV142928, ES905716,EV028082, ES913429,EE465196	2	2	11
BnaZIK3	EE472372	0	1	1
BnaZIK4	EV073626,EV139126,EV138473,EV138658,EV197022,EV150739,EV150892,EV070186,EE492290,EE490805,EE490822,EE494296,EE496988,EE496899,EE496635,EE492085,EE495787,EE492932,EE495844,EE491945,EE491291,EE497772,EE496649,EE493567,EV100529, EE432777,FG561292,EV190872, EV197102, EL625512, EV100585, EV190796,EE563876,CX278066,CX189467	4	3	35
BnaZIK5	EE536542,GR458826,EV195091,CD836798,GT084453,GR462753,EE444646,EV134286,EV133959,EV006837,EE471093, CD835586, CD835289,GR463177,CD832636, EV163147,EV195009,EV162973,EV194388,EV194455,CN728521,EV216646,EV061853, EV137850, EV138327, EV138063	4	2	26

BnaZIK6	FG568049,ES980749,GR455306,ES983869, DY020517,EE411206,EV217230,EE419889,EV125474,EV181844,EV181764,GR457279,EV103908,EV104150,EV204069,EE566985, EV125550	2	1	17
BnaZIK7	EE536542	0	1	1
BnaZIK8	GT085120,GT084787,EL591342,EV032757,EV104353,EV104948,EV104623,EV104261,EV068090,EG020399,CN726130,EV163028, EV163329,ES922711,EV049769	3	1	15
BnaZIK9	EV184439,EV182994, ES905420, ES989300,EV184525, EV183080, EV197102 EV052012	2	2	8
BnaCTR1	ES989676,EE561092,ES909336,EV086661,ES980529,ES916126,ES981393,ES908728,EV118427,EV122652,EV132908,FG576903,ES913258,CX196077,EL629678,ES952663,EL586905,CJ999426,ES992664,EV192459,EE562099, CX188622,ES953149,ES964092,EV011520,EV026142,EV039146,EE421469,EV122573,EV132704	3	0	30
BnaEDR1 (Raf2)	EE557579	0	0	1
BnaRaf3	FG553342,EE492012,EE497602,EE494763,EE497280,EE490955,EE493282,EE497282,EE495237,EE496270,EE497166,EE491978,EE493180,EE492484,EE493124,EE495719,EE494086,EE497526,EE492074,EE493713,EE495879, EE491260,EE498010,EE497752,EE493692,EE491100,ES974247,EV117365,EV117280,EE473862,EE564679, ES957467,EV013879 ,EV058376 ,EL589581	3	0	35
BnaRaf4	EV175536 ,EV175611	1	0	2
BnaRaf6	EV205676 , EV205897 ,EV112696,EV065524 ,ES905061 ,EV114878, EV112785	3	0	7
BnaRaf7	GT077030	0	1	1
BnaRaf9	GR441138,CX193045, EV216923	1	**1**	3
BnaRaf11	ES900067,GR440297,EV181221,EV181137,ES898401,EE415962,ES898940,EE437643,CX281039,EE415166,EE411762,DY005002,ES912117,FG560755,EV198857,CX189012,GR441948,GR441859,EV217100,EV192425	3	3	20
BnaRaf13	CX192251,EE435654,EV202317,EE435564,EV122200,EV122282,EV198765,ES908916,EV198840	3	1	9
BnaRaf12	CX280761,CD836474 ,CD836011,EV152662, EE549634, EE568144	2	2	6
BnaRaf15	EV042506,ES967579	0	2	2
BnaRaf16	ES271921, ES271656, GR459694, ES271811,GR454502 , GR456294 ,GR458356, EV012120	3	1	8
BnaRaf17	EV193963,EV143899,EL629490,EV198792, EV204737, EE444321, ES909131, EV198869, EV008880, CX188807, EV143538, ES901419, EV193903, EV071758, EE513821, EV194231, EE410295	3	3	17
BnaRaf18	EV168971, G566845,CN726119,EV145114,EV145484, EE470702, EE555040, DY010700,EV181902,EE433611, EV181814, EV182318 , EV121398 , EV121321	3	1	14
BnaRaf20	EV148544 , EE445303 ,EE558140 , EV148393, EV211763	2	1	5

BnaRaf21	CX188760,EE392444,EV045763,EV131126,EV159014,GR442116,ES903985,ES912963,EE549185,CD828271,EV198342,GR441411,EV216575,EV200708, EV057267, EV161604, EV161528, EV121092, EV191002,FG562361, EV081150,EV168660, EV121003,EV190923, EV161401, EV161312,EV161685, EE455467,EV010619,EV168718, EV110458,EV110382, EV202815, EV110158, EV200634,EV110207, EE459573, EV222182, EV189732,EV175173, DY001106,EV182843, EE478620,GR450566,ES903484,EV176650,EV176575, EV131046, EV020832,EL622888, ES900851	9	6	51
BnaRaf22	CD828801,DY012414,FG555341,EE411079,EV053578,EE541889,EV038508,EV078680,EV176349,EV176422,ES989778,EV117988, EL623219,ES991338,EV120911, GR447086,EE434452,GR441880,FG567395,EV036952,DY001930,EV120893,EV088066,EV214281,EV214617,EV141062,ES966119, EV013186,EE536863, EL625655, EV059455, CD842174, EV186139,EV041195, ES909882, EE449173, EV123588, GT073523,CD833520,CD832597, CD835829,EV023686,CN826331,EV185811,EE405899,ES989158,EV062246,EV084408,EE568957,EE568610,EE470507,EV050318,ES990445,FG561994,FG556992,EV079399,EV076551,FG574371,FG554400,EE468789,FG553318,DY002182,EV142833	7	3	63
BnaRaf23	CB686056,EE483168,EV080799,EV171789,AY587554,AY587554,ES994863,CN827635,CN827634,EV171782,EV171787, ES907355,EE445038,ES908335,EV063584, CN728271,GR455301,ES985384, GR456681, ES900514, EV107604,EV107986,EV009491,EE552793	5	4	24
BnaRaf24	GR440156,GT084680,EV023244,EE463020,EV038513,EV177532,EV223456,EE489551,EV223237,EV168802,CX188972,CX196226,FG575581,EV020291,ES963392,ES973776,ES973772,EV130809,EV021734,EL628173,EV224524,EV224626,EV224194,EV224283,EE506566,EV145157,EV145499,EV207344,CD837080	3	1	29
BnaRaf26	ES989096,EV155356,GR454201,DY021755,GR453562,CX195388,CX195380,EV109439,EV155273	1	0	9
BnaRaf27	EV189564,GT085380,H74387,GT085282,GT084914,GT084955, EE484013,EV189638,EV165926,EV221771, EE440826,H74653 ,H74454,H74614,H74390,EV165995, H74431	2	3	16
BnaRaf28	CD842174,EV186139,EV041195,ES909882,EE449173,EV123588,GT073523,CD833520,CD832597,CD835829,EV023686,CN826331,EV185811,EE405899,EE537024,EL626484,EE458599,CD836673,CN830316,CN830315,ES904982,EV132004,EV132281,ES907483,EV190689,CB686365,FG576634,EV109612,CN730941,EV131716,EE404614,EV123510,EV190606,EV109226,FG571495,EV034938,GR442269, EL623219, ES991338, EV120911, GR447086, EE434452, GR441880, FG567395, EV036952, DY001930, EV120893, EV088066,EV214281, EV214617,EV141062,ES966119,FG567592, DW998470, FG572537, CD831261, CD835589, CD831446, FG568749, ES989158, EV062246,EV084408, EE568957, EE568610, CD832942, ES990066, EV003278, EE538539, EV002846, EV033982, EV012823, EV012866, CD836853	8	2	73
BnaRaf29	EV057267,EV161604,EV161528,EV121092,EV191002,FG562361,EV081150,EV168660,EV121003,EV190923,EV161401,EV161312,EV161685,EE455467,EV010619,EV168718	1	0	16

BnaRaf30	GR450566,ES903484,EV176650,EV176575,DY001106,EV182843,EE478620,ES903985,ES912963,EE549185,EV222182,EV189732,EV175173,ES959642,EV011656,ES907290,CD828107,ES906772,EV189809,EV182928,EE454914,EE419315	6	2	22
BnaRaf31	EE562897 , EE562760	1	0	2
BnaRaf32	EV148003,EV014780,EV147698,ES900634,ES900648	**1**	0	5
BnaRaf33	CD837014,CX278481,DY025237,EV022140,CX280005,EE444749,EE419158,GR439687,EE457604	3	0	9
BnaRaf34	EE562760,EE562897	1	0	2
BnaRaf35	EV207803,EV207443,CX188942,EL588144,GR438570,EL630251,EE494265,EE496543,EE495641, FG557003	1	1	10
BnaRaf36	GR454194, EV126104	0	2	2
BnaRaf37	EE449283, EE450402, EV022618, EV070828, GR44340	0	5	5
BnaRaf38	CD837014,CX278481,DY025237	1	0	3
BnaRaf39	ES902728,ES271574,EV131406,EV206069,EE467568,GR459322,EV036406,GR462543,GR457156,FG554126,GR462574,EV002033,EV131215,EV205655,ES982394,EE435970,EV014667,GR453462,GR439048,GR459363,EV222031,EV222394, EE554782,GR453503, GR453297,ES965014,FG568031,EV031135,EE567796	4	3	29
BnaRaf41	EL623529,EE553522,CN831212,EE469147,EV109838,CN727032	1	1	6
BnaRaf42	FG566845,CN726119,EV145114,EV145484,EE470702,EE555040,GR440156,GT084680,EV023244,EE463020,EV038513,EV177532,EV223456,EE489551,EV223237,EV168802,CX188972,CX196226,FG575581,EV020291,ES963392,ES973776,ES973772,EV130809,EV207803,EV207443,CX188942,EL588144,GR438570,EL630251,EE494265,EE496543,EE495641	3	0	33
BnaRaf43	EE423013,EV013275,EE438214,EL623537,GR457543,EV123924,GR456382,EE477516,EE418279,EE566532,EE569368,EE538516,GR443324,EV048864,EL591064,EE414233	3	1	16
BnaRaf46	CN728271, GR455301,ES985384,GR456681, EV163620, EV080799	1	2	6
BnaRaf47	EV009491, EE552793	1	0	2
BnaRaf48	ES910727,ES902967,EL628100,ES905974,EV219602,EV219362,EV134513,EV110252,EV110524,EV120338,ES925881,ES910160,ES907733,FG566519,EV120257,EV142866,GT084966,GT085401,EE538279,EV087758,EV142503,ES913826,EV116485,EV168494,EV168531,EV116565 ,CD831398,ES910727	5	1	28

These EST were identified using the cDNA sequences of 80 Arabidopsis MAPKKK genes as the queries against the *Brassica napus* (Bna) EST database in NCBI (www.ncbi.nlm.nih.gov/dbEST/index.html) with a cut-off E value of 10^{-4}. The ESTs were clustered into contigs or singlets using a criteria of more than 95% similarity over a range of 50 bp or more. The GenBank accession numbers of each EST were listed after each individual BnaMAPKKK genes.

Table S2. Primers used in this study.

**Gene**	**primer sequence (5'→3')**
BnaMAPKKK17	Q-F:GACGGCGACGCAACTCCTAAAC
	Q-R:GACCCATCCAACCCACCAACAC
	F:cgccATGGAATGGACTAGAGGAAGAATCC
	R:cgggatccTCAATTTACAGTCCCCCCCAC
	BiFC-F:cgGGGCCCGAATGGACTAGAGGAAGAATCCTAGG
	BiFC-R:cgggatccTCAATTTACAGTCCCCCCCAC
BnaMAPKKK18	Q-F:AGACGCCTGAGCTGCCTTC
	Q-R:TTAATAATTGCCTCGCTGTCCATCT
	BiFC-F: ggactagtAATTGGACTAGAGAAAAAACAATAGGCC
	BiFC-R :cgcgtcgacATCATTATTCCGTCGAACCGTGATC
	OE-F:cgccATGGACAATTGGACTAGAGAAAAAACAATAGGCC
	OE-R:<br>gcTCTAGAgAGCGTAATCTGGAACATCGTATGGGTAAGCGTAATCTGGAACATCGT<br>ATGGGTATCCACCTCCGGGCGCGCCGTCGACGGGCCCATCATTATTCCGTCGAAC<br>CGTG
	BnaM3K18-K32R-F:ACC ATC GCC GTG GGA TCC GCC GAG
	BnaM3K18-K32R-R:GTG AAA CTC GGC GGA TCC CAC GGC GAT
	BnM3K18-GW-NcoI-F:AAAGCAGGCTTCcATGggtAATTGGACTAGAGAAAAAACA
	BnM3K18-GW-SalI-R:GTCTCCACCTCCGGA**gtcgac**ATCATTATTCCGTCGAACCGT
BnaMAPKKK19	Q-F:GAGGCGCCGGAGGAGAG
	Q-R:CCGTACGGTGACCCAGTTA
	BiFC-F:ggACTAGTGAGTGGGTTCGAGGAGAAAC
	BiFC-R:cgcGTCGACCCGTACGGTGACCCAGTTATTG
	OE-F:cgCCATGGAGTGGGTTCGAGGA
	OE-R:<br>gcTCTAGAgCAGATCTTCTTCAGAGATCAGTTTCTGTTCCAGATCTTCTTCAGAGAT<br>CAGTTTCTGTTCTCCACCTCCGGGCGCGCCGTCGACGGGCCCCCGTACGGTGACCC<br>AGTTATT
BnaMAPKKK20	Q-F:TATGTCGTCCGAGTCAGAGATTCAA
	Q-R:GACCCAGCCTTCGGAGACAGAC
	F:cgccATGGAGTGGGTTCGTGGAG
	R:cgcgtcgacCCGTACGGTGACCCAGCC
	BiFC-F:ggactagtGAGTGGGTTCGTGGAGAAAC
	BiFC-R:cgcgtcgacCCGTACGGTGACCCAGCC
BnaZIK2	Q-F:GCTGACGGAAGAGGCTAGAATAAGA
	Q-R:CCGCGTAAGGTGGCTGAAAC
	F:catgccATGGTAAACATGAATCAGCTTTCTGAGTATGTAG
	R:cgcgtcgacCTACCGCCTTGAACTCGGTGG
	BiFC-F:ggactagtATGGTAAACATGAATCAGC
	BiFC-R:cgcgtcgacCTACCGCCTTGAACTCGGTGG
BnaZIK3	Q-F:TGGCCGGAGAATGAGATAAGGAGA
	Q-R:GGCGTCGACGGGAAGTGAGA
	F:cgccATGGACAATGGTGAAGAAAGCTTCGTTGAAG
	R:cgggatccTCATATATCCACGGCGTCGAC
BnaZIK4	Q-F:GCAACAGCAGGAGAGCCGAGA
	Q-R:ATAGAGACGGTAAAAACGAGAACGA
	F:catgccATGGACAACAATCTGGTGAGTTATCTTGAGC
	R:cgcgtcgacAGATTCAATGGCATCGACAGG
BnaZIK8	Q-F:ACCCCGCCATGACCGAGAG
	Q-R:CAAGGCGATGATGCTGCTGTTC
	F:catgccATGGTACTTCCTTGTGCGAGCTCC

	R:cgggatccTCAAGAAACAACACGACCAGTTCC
BnaRaf17	Q-F:CGAGCCATACGAAGCAGCAAAAC
	Q-R:AGCCCCAGTGGTGATCAGAAGGTA
	F:cgccATGGAGAGCTCCGAAGCGTCGGCGGCT
	R:cgcgtcgacTGAAGTGAATAAGCCCCAGTGG
BnaRaf28	Q-F:AACGGTCCCAGCTGATTGTCTCC
	Q-R:AACGGGCTTTCCTCGCAGTCG
	F:cgccATGGAGCTTGAGGGACCTAAGTTCGATATGC
	R:cgcgtcgacGTCGATTGTCATTGGTTGCGTC
	BiFC-F:ggactagtATGCTTGAGGGACCTAAG
	BiFC-R:cgcgtcgacGTCGATTGTCATTGGTTGCGTC
BnaRaf29	Q-F:GCGATCGTGCTGTGGGAACTT
	Q-R:ATCTGGTCTTTGAGCTGGGTCTTGT
	F:cgccATGGCGATCAAAGACGAGACG
	R:cgcgtcgacATGGTGGCCTTTTCTTAGACCTG
BnaRaf30	Q-F:CTACGGGATTGTGCTATGGGAGTT
	Q-R:GGATGCGTCTTCTTCGGTATTTTC
	F:ttaggtcATGATCGTGGTGATGGAGAGTTGCGG
	R:cgcgtcgacTCAATGATGTGTTGCACTTCTCCT
BnaRaf34	Q-F:CACTATTGCCACCGGATTCACCT
	Q-R:TTTGTCTTCAGTTTTAGCCTTTGGAGT
	F:cttaggtcATGATCCTTGCTATGGATTCATTGAATGGATACAG
	R:cgcgtcgacATAACATTGATTGAAGCAGAAAAACAAACC
BnaRaf35	Q-F:GGAAAGTTGTTGGGCATCTGAGC
	Q-R:CTGCACCATCGCCATCTTCTTTT
	F:GGAATTCATGTGCAGCAGTAATCGCG
	R:cgcgtcgacTTTCAAATTCATAGCAGCAGCCATATTC
BnaRaf36	Q-F:GCTCTGCCATGCCTAACCCTAAAT
	Q-R:TCCCATAAACATCGAATCACAAACC
	F:cttaggtcatgATCGATGAAGAGGCTTCTTCTTGGATT
	R:cgcgtcgacAGCGAATTTAGGGTTAGGCATG
BnaRaf39	Q-F:CAGAGATGGAGGAAGTCGTGAAGA
	Q-R:ATAATAAGATGGCCAAAGAGGGGTAA
	F:cgCCATGGAAACACCAAACGAGATAAAAGCTT
	R:ccCCCGGGTCAAGGACCACGACCGGAG
BnaCTR1	F:ttaggtcATGATCGAAATGCCCGGCGCTAGAAG
	R:ccCCCGGGCATATCCAAGCGATTAGGTGGAGGAAC
BnaRaf21	F:cgCCATGGATACGAATACAGATGAATCGGAGAG
	cgcGTCGACTGGTACGACGTTTACTTCAGTCATTAT
BnaRaf22	F:cgCCATGGTACTTGACGGAGCAAAGTTCAAC
	cgggatccTTAGTCGATAGTCATAGGTTGACTCATAC
BnaRaf23	F:cgCCATGGCGAACATCGCGGGACA
	cgcGTCGACTTTCCAAGGAAACTTGAATGTATCTTTC
BnaRaf27	F:cgCCATGGAAGATGATTACAAAACGCCG
	R:cgcGTCGACCAAATGTGAACCGGATGATGAG
BnaRaf33	F:cgCCATGGTAAAGGAAGGAAACGACGGGT
	R:cgcGTCGACCCAAACAGAACAAAAACAAGGAAAGTTTAA
BnaRaf37	F:cgCCATGGTACCTCGCGGGTACCAACGA
	R:GGAATTCCTTGGAAATACAATCTCTGAAGATCTTGAG
BnaRaf41	F:ttaggtcATGATCGGATCTGCAAGTGGGTTTTACTC
	R:cgcGTCGACTGAGCAGCAGAAGAAGAAGCT
BnaRaf46	F:cgCCATGGAGAACGTAGCGGCGC

	R:cgcGTCGACTTTCCAAGGAAACTTGAATGTGTCTTTCC
BnaZIK5	F:ggaattcCATATGGACGGTGCAGAGGATGC
	R:cgcGTCGACACCAGCTTGTTCTACCTTCTTCTGAGATAAC
BnaZIK6	F:ggaattcCATATGGCTTTTGGATCTTCTGGAACT
	R:cgggatccTCACAAACCTTTTTGCTTTTTCATAATCC
BnaZIK9	F:cgCCATGGTAATGAACAACATCAGCCATCTTGAATC
	R:cgcGTCGACGTCACTTGACCTACAAGAAAACACC
BnaMKK1	F: GGAATTCCATATGAAGAGAGGCAGCTTGAGTC
	R: CGGGATCCTAGCTGTTAGCTAGTGGGGG
BnaMKK2	F: GGAATTCCATATGAAGAAGGGTGGATTCAGCAATAATC
	R: CGGGATCCTTAAACGGAGAATGTACCAGACAGATTC
	BiFC-F:cgGGATCCATGAAGAAGGGTGGATTCAG
	BiFC-R:CGCGTCGACAACGGAGAATGTACCAGAC
BnaMKK3	F: GGAATTCCATATGGCAGCATTGGAGGAACTA
	R: CGGGATCCTTAACTTGTAATATAAAGCTCTTGCTTAGC
	BiFC-F:gcTCTAGAATGGCAGCATTGGAGGAACTA
	BiFC-R:gggatccACTTGTAATATAAAGCTCTTG
BnaMKK4	F: GGAATTCCATATGAAACCAATCCAACCGCC
	R: CGGAATTCTACGTCGTTGGGGAAGAAGAAG
BnaMKK5	F:ggaattccatATGGCCATGAGACGAAACAAACGTCCT
	R:cgcgtcgacCTAAGACGCCGGAGGCGGCG
	BiFC-F:gctctagaATGGCCATGAGACGAAAC
	BiFC-R:CGCGTCGACAGACGCCGGAGGCGGCG
BnaMKK6	F:ggaattccatATGAAGCTCAAATCGAACTTGAAGC
	R:cggaattcTTATCTAAGGCAGTTAACAGGTGGTTC
	BiFC-F:gctctagaATGAAGCTCAAATCGAACTTG
	BiFC-R:CGCGTCGACTCTAAGGCAGTTAACAGGTG
BnaMKK8	F: gcCCATGggcAATCTCAAGCTTTCTCTTCCAC
	R: cGAGCTCATCTTGCTGGAGAAGAAAAGGG
BnaMKK9	F:ggaattccatATGGCTCTTGTTAGAGAACGTCG
	R:cgggatccTCAAAGCTCTTCCTGGAGAAAAG
	BiFC-F:cgGGATCCATGGCTCTTGTTAGAGAACGT
	BiFC-R:cgcGTCGACAAGCTCTTCCTGGAGAAAAG
	BnaMKK9-K74R-Fwd AACACTCTCTACGCTCTGAGAACAGTCAACGGC
	BnaMKK9-K74R-Rvs GTCGCCGTTGACTGTTCTCAGAGCGTAGAGA
	MKK9-GW-F GGGGACAAGTTTGTACAAAAAAGCAGGCTTCATGGCTCTTGTTAGAGAACGTCG
	MKK9-GW-R GGGGACCACTTTGTACAAGAAAGCTGGGTCTCAAAGCTCTTCCTGGAGAAAAGG
	BnaMKK9-S193D/S199E-F GTCCGGGAATTGGATTCTTGCAACGAATAT
	BnaMKK9-S193D/S199E-R GACATATTCGTTGCAAGAATCCAATTCCCG
U5-attB1	G GGG **ACAAGTTTGTACAAAAAAGCAGGCT**TCc**ATG**
U3-attB2	GGG G**ACCACTTTGTACAAGAAAGCTGGGTC**TCCACCTCCGGA
BnaUP1	Q-F: AGCCTGAGGAGATATTAGCAGGAA
	Q-R: ATCTCACTGCAGCTCCACCAT
BnaUBC9	Q-F: GCATCTGCCTCGACATCTTGA
	Q-R: GACAGCAGCACCTTGGAAATG

Note: F and R are primers used for amplifying CDS and cloned into pGBKT7 or pGADT7. Q-F and Q-R are for real-time PCR, and GFP-F and GFP R are for GFP fusion. BiFC-F and BiFC-R are used for in vivo bimolecular florescence complementation (BiFC) analysis.

Table S3. MAPKKK sequences from different species used for phylogenetic analysis.

Species	Taxon name	Synonyms	GenBank Accession No. or locus No.	Subfamily
Arabidopsis thaliana	AtMAPKKK1	AtANP1	At1g09000	MEKK
	AtMAPKKK2	AtANP2	At1g54960	MEKK
	AtMAPKKK3	AtMAPKKK3α	At1g53570	MEKK
	AtMAPKKK4		At1g63700	MEKK
	AtMAPKKK5	AtMAP3Kγ	At5g66850	MEKK
	AtMAPKKK6	AtMAP3Kε2	At3g07980	MEKK
	AtMAPKKK7	AtMAP3Kε1	At3g13530	MEKK
	AtMAPKKK8	AtMEKK1	At4g08500	MEKK
	AtMAPKKK9		At4g08480	MEKK
	AtMAPKKK10	AtMEKK3	At4g08470	MEKK
	AtMAPKKK11	AtMEKK4	At4g12020	MEKK
	AtMAPKKK12	AtANP3	At3g06030	MEKK
	AtMAPKKK13		At1g07150	MEKK
	AtMAPKKK14		At2g30040	MEKK
	AtMAPKKK15		At5g55090	MEKK
	AtMAPKKK16		At4g26890	MEKK
	AtMAPKKK17		At2g32510	MEKK
	AtMAPKKK18		At1g05100	MEKK
	AtMAPKKK19		At5g67080	MEKK
	AtMAPKKK20		At3g50310	MEKK
	AtMAPKKK21		At4g36950	MEKK
	AtZIK1	WNK5	At3g51630	ZIK
	AtZIK2	WNK4	At5g58350	ZIK
	AtZIK3	WNK2	At3g22420	ZIK
	AtZIK4	WNK1	At3g04910	ZIK
	AtZIK5	WNK6	At3g18750	ZIK
	AtZIK6	WNK8	At5g41990	ZIK
	AtZIK7	WNK7	At1g49160	ZIK
	AtZIK8		At5g55560	ZIK
	AtZIK9	WNK9	At5g28080	ZIK
	AtZIK10	WNK10	At1g64630	ZIK
	AtZIK11	WNK3	At3g48260	ZIK
	AtRaf1	AtCTR1	At5g03730	Raf
	AtRaf2	AtEDR1	At1g08720	Raf
	AtRaf3	AtMAP3Kδ1	At5g11850	Raf
	AtRaf4		At1g18160	Raf
	AtRaf5		At1g73660	Raf
	AtRaf6	AtMAP3Kδ5	At4g24480	Raf
	AtRaf7		At3g06620	Raf
	AtRaf8		At3g06630	Raf
	AtRaf9		At3g06640	Raf

	AtRaf10	AtMAP3Kh3	At5g49470	Raf
	AtRaf11		At1g67890	Raf
	AtRaf12	AtMAP3Kδ4	At4g23050	Raf
	AtRaf13	AtMAP3Kq1	At2g31010	Raf
	AtRaf14	AtMAP3Kq2	At2g42630	Raf
	AtRaf15		At3g58640	Raf
	AtRaf16		At1g04700	Raf
	AtRaf17		At1g14000	Raf
	AtRaf18		At1g16270	Raf
	AtRaf19		At1g62400	Raf
	AtRaf20		At1g79570	Raf
	AtRaf21		At2g17700	Raf
	AtRaf22		At2g24360	Raf
	AtRaf23		At2g31800	Raf
	AtRaf24		At2g35050	Raf
	AtRaf25		At2g43850	Raf
	AtRaf26		At4g14780	Raf
	AtRaf27		At4g18950	Raf
	AtRaf28		At4g31170	Raf
	AtRaf29		At4g35780	Raf
	AtRaf30		At4g38470	Raf
	AtRaf31		At5g01850	Raf
	AtRaf32		At5g40540	Raf
	AtRaf33		At5g50000	Raf
	AtRaf34		At5g50180	Raf
	AtRaf35		At5g57610	Raf
	AtRaf36		At5g58950	Raf
	AtRaf37		At5g66710	Raf
	AtRaf38		At3g01490	Raf
	AtRaf39		At3g22750	Raf
	AtRaf40		At3g24720	Raf
	AtRaf41	ATN1	At3g27560	Raf
	AtRaf42		At3g46920	Raf
	AtRaf43		At3g46930	Raf
	AtRaf44		At3g50720	Raf
	AtRaf45	ATN1-like	At3g50730	Raf
	AtRaf46		At3g59830	Raf
	AtRaf47		At3g58760	Raf
	AtRaf48	AtMRK1	At3g63260	Raf
Brassica napus	BnaMAPKKK3	BnMAP3Kα1	CAA08995	MEKK
	BnaMAPKKK6	BnMAP3Kε1	CAB54520	MEKK
	BnaMAPKKK8	BnMAP3Kβ1	CAA08997	MEKK
	BnaMAPKKK17		KC190095	MEKK
	BnaMAPKKK18		KC190096	MEKK
	BnaMAPKKK19		KC190097	MEKK
	BnaMAPKKK20		KC190098	MEKK
	BnaZIK2		KC190099	ZIK

	BnaZIK3	KC190100	ZIK
	BnaZIK4	KC190101	ZIK
	BnaZIK5	TC171979	ZIK
	BnaZIK6	KF129405	ZIK
	BnaZIK8	KC190102	ZIK
	BnaZIK9	KF129406	ZIK
	BnaRaf17	KC190103	Raf
	BnaRaf28	KC190104	Raf
	BnaRaf29	KC190105	Raf
	BnaRaf30	KC190106	Raf
	BnaRaf34	KC190107	Raf
	BnaRaf35	KC190108	Raf
	BnaRaf36	KC190109	Raf
	BnaRaf39	KC190110	Raf
	BnaCTR1	KF129395	Raf
	BnaRaf21	KF129396	Raf
	BnaRaf22	KF129397	Raf
	BnaRaf23	KF129398	Raf
	BnaRaf27	KF129399	Raf
	BnaRaf33	KF129400	Raf
	BnaRaf37	KF129401	Raf
	BnaRaf41	KF129402	Raf
	BnaRaf46	KF129403	Raf
Brassica rapa	BraMAPKKK1	Bra018588	MEKK
	BraMAPKKK4	Bra027792	MEKK
	BraMAPKKK5	Bra012110	MEKK
		Bra031828	MEKK
	BraMAPKKK6	Bra001280	MEKK
	BraMAPKKK7	Bra027424	MEKK
		Bra039396	MEKK
		Bra001510	MEKK
	BraMAPKKK8	Bra000647	MEKK
		Bra039693	MEKK
	BraMAPKKK10	Bra037860	MEKK
	BraMAPKKK11	Bra029454	MEKK
	BraMAPKKK12	Bra020785	MEKK
	BraMAPKKK13	Bra015556	MEKK
	BraMAPKKK14	Bra018344	MEKK
		Bra022773	MEKK
	BraMAPKKK15	Bra002933	MEKK
		Bra035552	MEKK
		Bra028991	MEKK
	BraMAPKKK16	Bra026395	MEKK
		Bra010423	MEKK
	BraMAPKKK17	Bra018228	MEKK
		Bra021794	MEKK
		Bra005599	MEKK

BraMAPKKK18	Bra015388	MEKK
BraMAPKKK19	Bra012131	MEKK
BraMAPKKK20	Bra036070	MEKK
	Bra030001	MEKK
BraMAPKKK21	Bra011739	MEKK
BraRaf1	Bra009514	Raf
	Bra005778	Raf
BraRaf2	Bra031639	Raf
BraRaf3	Bra006122	Raf
	Bra023347	Raf
BraRaf4	Bra025927	Raf
BraRaf5	Bra015976	Raf
BraRaf6	Bra013806	Raf
BraRaf7	Bra029594	Raf
	Bra040282	Raf
	Bra040281	Raf
BraRaf9	Bra001205	Raf
BraRaf10	Bra036141	Raf
BraRaf12	Bra019338	Raf
BraRaf13	Bra021687	Raf
	Bra022820	Raf
BraRaf15	Bra007408	Raf
	Bra003347	Raf
BraRaf16	Bra015343	Raf
BraRaf17	Bra019692	Raf
	Bra026867	Raf
BraRaf18	Bra026049	Raf
	Bra026726	Raf
	Bra026072	Raf
BraRaf19	Bra027048	Raf
	Bra036661	Raf
	Bra027847	Raf
BraRaf20	Bra035125	Raf
	Bra003603	Raf
BraRaf21	Bra002078	Raf
	Bra024497	Raf
	Bra037258	Raf
BraRaf22	Bra007857	Raf
	Bra032102	Raf
	Bra007860	Raf
BraRaf23	Bra021744	Raf
	Bra022866	Raf
BraRaf24	Bra021945	Raf
	Bra022999	Raf
BraRaf26	Bra037067	Raf
BraRaf27	Bra013363	Raf
	Bra020982	Raf

BraRaf28	Bra011250	Raf
	Bra024020	Raf
	Bra010247	Raf
	Bra007859	Raf
BraRaf29	Bra010516	Raf
BraRaf30	Bra011897	Raf
	Bra010744	Raf
	Bra015215	Raf
BraRaf31	Bra028913	Raf
BraRaf32	Bra025563	Raf
BraRaf33	Bra000605	Raf
BraRaf34	Bra000612	Raf
BraRaf35	Bra006812	Raf
BraRaf36	Bra020343	Raf
	Bra006738	Raf
	Bra002606	Raf
BraRaf37	Bra012094	Raf
	Bra037152	Raf
BraRaf38	Bra039114	Raf
BraRaf39	Bra033921	Raf
	Bra023811	Raf
BraRaf41	Bra025286	Raf
BraRaf43	Bra018183	Raf
BraRaf46	Bra007480	Raf
	Bra003392	Raf
BraRaf47	Bra007418	Raf
	Bra014553	Raf
BraRaf48	Bra007738	Raf
	Bra040440	Raf
BraZIK1	Bra036787	ZIK
BraZIK2	Bra002658	ZIK
	Bra020372	ZIK
	Bra006771	ZIK
	Bra029915	ZIK
BraZIK3	Bra039952	ZIK
	Bra001867	ZIK
BraZIK4	Bra040414	ZIK
	Bra001129	ZIK
BraZIK5	Bra037565	ZIK
	Bra001718	ZIK
BraZIK6	Bra028518	ZIK
BraZIK8	Bra002897	ZIK
	Bra035577	ZIK
	Bra028972	ZIK
BraZIK9	Bra009990	ZIK
	Bra036118	ZIK
BraZIK11	Bra019537	ZIK

Oryza sativa	OsMAPKKK1	OsACDR1,OsEDR1	Os03g06410/AY167575	Raf
	OsMAPKKK2		Os10g29540/AK121718	Raf
	OsMAPKKK3		Os02g32610/AAF34436	Raf
	OsMAPKKK4		Os02g12810	Raf
	OsMAPKKK5		Os12g37570	Raf
	OsMAPKKK6	DSM1	Os02g50970/AK102767	Raf
	OsMAPKKK7		Os06g12590/AK099500/AK105681	Raf
	OsMAPKKK8		Os11g10100	MEKK
	OsMAPKKK9		Os02g44642/AK073040	MEKK
	OsMAPKKK10		Os04g47240	MEKK
	OsMAPKKK11		Os07g02780/AK069889	MEKK
	OsMAPKKK12		Os09g39320	Raf
	OsMAPKKK13		Os09g21510	MEKK
	OsMAPKKK14		Os04g52140/AK120898	Raf
	OsMAPKKK15		Os08g32600/AK103087	MEKK
	OsMAPKKK16		Os04g35700/AK061622/AK068725	MEKK
	OsMAPKKK17		Os09g37230/AK072690	Raf
	OsMAPKKK18		Os03g55560	MEKK
	OsMAPKKK19		Os02g35010/AK287889	MEKK
	OsMAPKKK20	OsWNK1	Os07g38530/DQ837532	ZIK
	OsMAPKKK21		Os07g25680/AK241123	Raf
	OsMAPKKK22		Os03g49640	MEKK
	OsMAPKKK23		Os12g40279	Raf
	OsMAPKKK24		Os04g56530/AK099839	MEKK
	OsMAPKKK25		Os02g38080	Raf
	OsMAPKKK26		Os07g29330/AK066198	Raf
	OsMAPKKK27		Os03g43760/AK243690	Raf
	OsMAPKKK28		Os03g15570/AK242766/CT835152/CT835203	MEKK
	OsMAPKKK29	OsWNK4	Os02g45130/AK073845	ZIK
	OsMAPKKK30		Os02g02780/AK060220	Raf
	OsMAPKKK31		Os01g45380/AK108130	Raf
	OsMAPKKK32		Os08g12750/AY156510	Raf

OsMAPKKK33		Os02g07790/AY156512	Raf
OsMAPKKK34		Os05g50190/AY224453	Raf
OsMAPKKK35		Os02g54510	Raf
OsMAPKKK36	OsWNK2	Os05g01780/AK070061	ZIK
OsMAPKKK37		Os04g51950/AY156511	Raf
OsMAPKKK38		Os06g45300/AY224431	Raf
OsMAPKKK39		Os06g08280/AK067771	Raf
OsMAPKKK40		Os01g48330/AK070808/AK102209 AK111983	Raf
OsMAPKKK41		Os06g43840/AK243413	Raf
OsMAPKKK42		Os03g60150/CT830832	Raf
OsMAPKKK43	ILA1	Os06g50920/AK073747	Raf
OsMAPKKK44		Os02g14530/AK111618	Raf
OsMAPKKK45		Os06g43030	Raf
OsMAPKKK46	OsWNK6	Os11g06140/AK070490	ZIK
OsMAPKKK47	OsWNK3	Os07g08750/AK060552/AK100930	ZIK
OsMAPKKK48		Os01g01740/AK059460	Raf
OsMAPKKK49		Os05g44290	Raf
OsMAPKKK50	OsWNK7	Os12g02250/AK067447/AK072172	ZIK
OsMAPKKK51		Os01g54350/AK102467	Raf
OsMAPKKK52	OsWNK9	Os12g06490/AK062812/AK073772	ZIK
OsMAPKKK53		Os11g02305	ZIK
OsMAPKKK54		Os03g28300/CT828499	Raf
OsMAPKKK55		Os01g50400	MEKK
OsMAPKKK56		Os05g01780/AY336987	ZIK
OsMAPKKK57		Os05g46750	MEKK
OsMAPKKK58		Os03g39150	Raf
OsMAPKKK59		Os12g41260/AK109696	Raf
OsMAPKKK60		Os03g53410	Raf
OsMAPKKK61		Os01g10450/AK069537	Raf
OsMAPKKK62		Os01g50420	MEKK
OsMAPKKK63		Os01g50370	MEKK
OsMAPKKK64	OsWNK5	Os07g39520/CT832200/AK072014 /AK072176/AK10526	ZIK

	OsMAPKKK65		Os07g43900	Raf
	OsMAPKKK66		Os10g04010	MEKK
	OsMAPKKK67	OsNPK1	Os10g04000	MEKK
	OsMAPKKK68		Os12g30570	Raf
	OsMAPKKK69		Os05g46760/AK105946	MEKK
	OsMAPKKK70		Os01g50410	MEKK
	OsMAPKKK71		Os02g21700	MEKK
	OsMAPKKK72		Os01g54480/AK104674	Raf
	OsMAPKKK73		Os03g18170	MEKK
	OsMAPKKK74		Os01g66860/AK070097	Raf
	OsMAPKKK75		Os02g39560/AK071922/AK099756	Raf
Selaginella moellendorffii	Sm86240		86240	MEKK
	Sm139837		139837	MEKK
	Sm65764		65764	MEKK
	Sm73721		73721	MEKK
	Sm113703		113703	MEKK
	Sm55695		55695	MEKK
	Sm82067		82067	MEKK
	Sm164743		164743	MEKK
	Sm76216		76216	MEKK
	Sm444546		444546	MEKK
	Sm438664		438664	MEKK
	Sm74353		74353	MEKK
	Sm73444		73444	MEKK
	Sm126389		126389	ZIK
	Sm134331		134331	ZIK
	Sm11317		11317	ZIK
	Sm130367		130367	ZIK
	Sm10499		10499	Raf
	Sm24258		24258	Raf
	Sm40493		40493	Raf
	Sm97070		97070	Raf
	Sm96997		96997	Raf
	Sm56634		56634	Raf
	Sm131553		131553	Raf
	Sm130298		130298	Raf
	Sm146775		146775	Raf
	Sm20996		20996	Raf
	Sm115356		115356	Raf
	Sm146897		146897	Raf
	Sm118910		118910	Raf
	Sm444789		444789	Raf
	Sm88196		88196	Raf

	Sm63609	63609	Raf
	Sm34528	34528	Raf
	Sm271276	271276	Raf
	Sm160379	160379	Raf
	Sm57136	57136	Raf
	Sm105905	105905	Raf
Physcomitrella patens	Pp1s151_4V6.1	Pp1s151_4V6.1	MEKK
	Pp1s151_3V6.1	Pp1s151_3V6.1	MEKK
	Pp1s216_64V6.1	Pp1s216_64V6.1	MEKK
	Pp1s8_299V6.1	Pp1s8_299V6.1	MEKK
	Pp1s387_39V6.1	Pp1s387_39V6.1	MEKK
	Pp1s10_200V6.1	Pp1s10_200V6.1	MEKK
	Pp1s44_113V6.1	Pp1s44_113V6.1	MEKK
	Pp1s351_40V6.1	Pp1s351_40V6.1	MEKK
	Pp1s35_323V6.1	Pp1s35_323V6.1	MEKK
	Pp1s136_5V6.3	Pp1s136_5V6.3	MEKK
	Pp1s31_252V6.1	Pp1s31_252V6.1	MEKK
	Pp1s43_178V6.1	Pp1s43_178V6.1	MEKK
	Pp1s20_383V6.1	Pp1s20_383V6.1	MEKK
	Pp1s281_41V6.1	Pp1s281_41V6.1	MEKK
	Pp1s99_12V6.1	Pp1s99_12V6.1	MEKK
	Pp1s281_44V6.1	Pp1s281_44V6.1	MEKK
	Pp1s100_149V6.1	Pp1s100_149V6.1	MEKK
	Pp1s17_36V6.1	Pp1s17_36V6.1	MEKK
	Pp1s66_286V6.1	Pp1s66_286V6.1	MEKK
	Pp1s131_142V6.1	Pp1s131_142V6.1	MEKK
	Pp1s188_1V6.1	Pp1s188_1V6.1	MEKK
	Pp1s68_168V6.1	Pp1s68_168V6.1	MEKK
	Pp1s462_10V6.1	Pp1s462_10V6.1	Raf
	Pp1s75_1V6.1	Pp1s75_1V6.1	Raf
	Pp1s11_61V6.1	Pp1s11_61V6.1	Raf
	Pp1s63_65V6.1	Pp1s63_65V6.1	Raf
	Pp1s43_196V6.1	Pp1s43_196V6.1	Raf
	Pp1s17_22V6.1	Pp1s17_22V6.1	Raf
	Pp1s257_31V6.1	Pp1s257_31V6.1	Raf
	Pp1s130_99V6.1	Pp1s130_99V6.1	Raf
	Pp1s31_3V6.1	Pp1s31_3V6.1	Raf
	Pp1s126_60V6.1	Pp1s126_60V6.1	Raf
	Pp1s46_110V6.1	Pp1s46_110V6.1	Raf
	Pp1s96_95V6.1	Pp1s96_95V6.1	Raf
	Pp1s120_103V6.1	Pp1s120_103V6.1	Raf
	Pp1s66_220V6.1	Pp1s66_220V6.1	Raf
	Pp1s43_45V6.1	Pp1s43_45V6.1	Raf
	Pp1s69_199V6.1	Pp1s69_199V6.1	Raf
	Pp1s281_5V6.1	Pp1s281_5V6.1	Raf
	Pp1s118_90V6.1	Pp1s118_90V6.1	Raf
	Pp1s35_214V6.1	Pp1s35_214V6.1	Raf

	Pp1s93_69V6.1	Pp1s93_69V6.1	Raf
	Pp1s131_116V6.1	Pp1s131_116V6.1	Raf
	Pp1s244_9V6.1	Pp1s244_9V6.1	Raf
	Pp1s172_82V6.1	Pp1s172_82V6.1	Raf
	Pp1s85_87V6.1	Pp1s85_87V6.1	Raf
	Pp1s31_260V6.1	Pp1s31_260V6.1	Raf
	Pp1s19_317V6.1	Pp1s19_317V6.1	Raf
	Pp1s136_112V6.1	Pp1s136_112V6.1	Raf
	Pp1s74_259V6.1	Pp1s74_259V6.1	Raf
	Pp1s402_19V6.1	Pp1s402_19V6.1	Raf
	Pp1s375_32V6.1	Pp1s375_32V6.1	Raf
	Pp1s248_25V6.1	Pp1s248_25V6.1	Raf
	Pp1s78_106V6.1	Pp1s78_106V6.1	Raf
	Pp1s180_6V6.1	Pp1s180_6V6.1	Raf
	Pp1s392_39V6.1	Pp1s392_39V6.1	Raf
	Pp1s237_2V6.1	Pp1s237_2V6.1	ZIK
	Pp1s116_192V6.1	Pp1s116_192V6.1	ZIK
	Pp1s384_6V6.1	Pp1s384_6V6.1	ZIK
	Pp1s236_45V6.1	Pp1s236_45V6.1	ZIK
	Pp1s236_53V6.1	Pp1s236_53V6.1	ZIK
Ostreococcus lucimarinus	OlRaf1	estExt_GenewiseEukaryote.C_Chr_90523|46664	Raf
	OlMAPKKK1	e_gwEuk.16.135.1|37745	MEKK
	OlRaf2	estExt_GenewiseEukaryote.C_Chr_50114|45682	Raf
	OlRaf3	e_gwEuk.12.171.1|39208	Raf
	OlMAPKKK2	e_gwEuk.13.217.1|41728	MEKK
	OlZIK1	estExt_fgenesh1_pg.C_Chr_80010|24885	ZIK
	OlRaf4	gwEuk.20.241.1|6189	Raf
	OlRaf5	gwEuk.11.188.1|3085	Raf
	OlRaf6	gwEuk.5.459.1|7730	Raf
	OlRaf7	e_gwEuk.5.319.1|38832	Raf
	OlRaf8	ost_05_007_076|87238	Raf
	OlRaf9	e_gwEuk.17.220.1|42011	Raf
	OlRaf10	gwEuk.14.176.1|3629	Raf
	OlRaf11	estExt_Genewise_ext.C_Chr_110034|50798	Raf
	OlRaf12	gwEuk.11.262.1|5051	Raf
Ostreococcus tauri	OtMAPKKKα1	XP_003082588	
Nicotiana tabacum	NtNPK1	AB055514	MEKK
Nicotiana benthamiana	NbMAPKKKα	AY500155	MEKK
	NbMAPKKKβ	AB649283	MEKK

	NbMAPKKKγ	AB649284	MEKK
	NbMAPKKKε2	AB649285	MEKK
Solanum lycopersicum	SlMAPKKKε	GU192457	MEKK
Lycopersicon esculentum	LeMAPKKKα	AY500156	MEKK
Drosophila melanogaster	DmWNK	AGB94867	ZIK
Caenorhabditis elegans	CeWNK	CCE71534	ZIK

The respective MAPKKK sequences, except those from canola, were either downloaded from the rice genome annotation project (http://rice.plantbiology.msu.edu), public NCBI database (http://www.ncbi.nlm.nih.gov) according to published papers in Pubmed, or identified from Phytozome database (www.phytozome.net) as described in the "Materials and methods" section. The protein name of each MAPKKK is depicted by a two to three-letter code denoting the species in combination with numbers or locus ID representing the chromosome localization in that species as shown in Phytozome database. The GenBank accession numbers represent the assigned codes of the sequences deposited in GenBank.

Table S4. Similarity and identity analysis of MAPKKK genes/proteins between Arabidopsis, rice and canola.

Please see the separate Excel file.

Table S5. Computational prediction of subcellular localizations of MAPKKK proteins in canola.

protein	Subcellular localization		
	**PSORT**	**CELLO**	**ESLpred**
BnaMAPKKK17	*chlo: 11.0, cyto: 1.0, mito: 1.0*	*Chloroplast 2.362 * Cytoplasmic 1.193*	*Cytoplasmic Reliability Index= 2 Expected Accuracy=~ 75%*
BnaMAPKKK18	*cyto: 11.0, chlo: 1.0, nucl: 1.0*	*Cytoplasmic 2.006 **	*Cytoplasmic Reliability Index= 3 Expected Accuracy=~ 94%*
BnaMAPKKK19	*cyto: 5.0, nucl: 3.0, chlo: 2.0, plas: 2.0, cysk: 1.0*	*Cytoplasmic 2.524 * Chloroplast 1.325*	*Cytoplasmic Reliability Index= 5 Expected Accuracy=~ 98%*
BnaMAPKKK20	*cyto: 6.0, nucl: 5.0, chlo: 3.0*	*Cytoplasmic 2.344 * Chloroplast 1.706 **	*Cytoplasmic Reliability Index= 3 Expected Accuracy=~ 94%*
BnaZIK2	*nucl: 8.0, cyto: 4.0, chlo: 1.0*	*Nuclear 2.246 * Cytoplasmic 1.05 PlasmaMembrane 0.833 Mitochondrial 0.475 Chloroplast 0.213*	*Cytoplasmic Reliability Index= 1 Expected Accuracy=~ 53%*
BnaZIK3	*nucl: 10.0, cyto: 2.0, cysk: 2.0*	*Cytoplasmic 2.521 * Nuclear 2.104 **	*Nuclear Reliability Index= 1 Expected Accuracy=~ 53%*
BnaZIK4	*nucl: 11.0, cyto: 2.0*	*Nuclear 3.247 * Cytoplasmic 1.479*	*Cytoplasmic Reliability Index= 2 Expected Accuracy=~ 75%*
BnaZIK5	*4.0, nucl: 2.5, cyto: 2.0, plas: 2.0, vacu: 2.0, cysk_nucl: 2.0*	*Cytoplasmic 4.266 * OuterMembrane 0.404 Extracellular 0.146 Periplasmic 0.115 InnerMembrane 0.070*	*Cytoplasmic Protein Details Reliability Index= 2 Expected Accuracy=~ 75%*
BnaZIK6	*nucl: 10.0, cyto: 2.0, chlo: 1.0*	*Extracellular 1.818 * Cytoplasmic 1.780 * OuterMembrane 0.740 Periplasmic 0.476 InnerMembrane 0.186*	*Nuclear Protein Reliability Index= 4 Expected Accuracy=~ 97%*
BnaZIK8	*cyto: 4.5, cyto_nucl: 4.5, nucl: 3.5, chlo: 3.0, cysk: 2.0*	*Cytoplasmic 2.280 * Nuclear 1.633 **	*Cytoplasmic Reliability Index= 3 Expected Accuracy=~ 94%*
BnaZIK9	*5.0, cyto: 5.0, cysk: 2.0, vacu: 1.0*	*Cytoplasmic 4.424 * Extracellular 0.349 OuterMembrane 0.122 Periplasmic 0.089 InnerMembrane 0.016*	*Nuclear ProteinReliability Index= 2 Expected Accuracy=~ 75%*

BnaCTR1	*chlo: 6.0, pero: 3.0, nucl: 2.0, mito: 2.0*	*Periplasmic 1.876 * Cytoplasmic 1.590 * OuterMembrane 1.044 InnerMembrane 0.249 Extracellular 0.242*	*Cytoplasmic Protein Details Reliability Index= 1 Expected Accuracy=~ 53%*
BnaRaf17	*cyto: 8.0, nucl: 3.0, plas: 2.0*	*Mitochondrial 1.991 * Cytoplasmic 1.959 **	*Cytoplasmic Reliability Index= 3 Expected Accuracy=~ 94%*
BnaRaf21	*cyto: 12.0, chlo: 1.0*	*Cytoplasmic 4.321 * Periplasmic 0.466 OuterMembrane 0.125 Extracellular 0.056 InnerMembrane 0.032*	*Cytoplasmic Protein Details Reliability Index= 5 Expected Accuracy=~ 98%*
BnaRaf22	*chlo: 7.0, cyto: 5.0, nucl: 1.0*	*Cytoplasmic 3.213 * Periplasmic 1.065 InnerMembrane 0.607 Extracellular 0.062 OuterMembrane 0.054*	*Mitochondrial Protein Details Reliability Index= 1 Expected Accuracy=~ 53%*
BnaRaf23	*mito: 7.5, chlo_mito: 6.2, cyto_mito: 5.3, chlo: 3.5*	*Cytoplasmic 3.843 * Periplasmic 1.103 InnerMembrane 0.028 OuterMembrane 0.018 Extracellular 0.008*	*Cytoplasmic Protein Details Reliability Index= 2 Expected Accuracy=~ 75%*
BnaRaf27	*cyto: 8.0, pero: 4.0, nucl: 1.0*	*Cytoplasmic 4.579 * Periplasmic 0.384 InnerMembrane 0.019 Extracellular 0.012 OuterMembrane 0.006*	*Cytoplasmic Protein Details Reliability Index= 1 Expected Accuracy=~ 53%*
BnaRaf28	*nucl: 3.0, cyto: 3.0, E.R.: 2.0, pero: 2.0, cysk: 2.0, chlo: 1.0*	*Mitochondrial 1.308 * Cytoplasmic 1.151 **	*Cytoplasmic Reliability Index= 1 Expected Accuracy=~ 53%*
BnaRaf29	*cyto: 9.0, nucl: 3.0, chlo: 1.0*	*Cytoplasmic 2.677 * Nuclear 0.919*	*Cytoplasmic Reliability Index= 4 Expected Accuracy=~ 97%*
BnaRaf30	*cyto: 11.0, chlo: 2.0*	*Cytoplasmic 3.297 **	*Cytoplasmic Reliability Index= 5 Expected Accuracy=~ 98%*
BnaRaf33	*cyto: 8.0, chlo: 2.0, nucl: 2.0, cysk: 2.0*	*Cytoplasmic 3.517 * Periplasmic 1.157 Extracellular 0.160 InnerMembrane 0.104 OuterMembrane 0.063*	*Cytoplasmic Protein Reliability Index= 3Expected Accuracy=~ 94%*
BnaRaf34	*cyto: 7.0, cysk: 3.0, chlo: 2.0, nucl: 1.0*	*Cytoplasmic 2.985 * Mitochondrial 0.897*	*Cytoplasmic Reliability Index= 4 Expected Accuracy=~ 97%*

BnaRaf35	*nucl: 7.0,<br>chlo: 5.0,<br>mito: 2.0*	*Nuclear 2.355 *<br>Cytoplasmic 1.938 **	*Nuclear Reliability Index= 4<br>Expected Accuracy=~ 97%*
BnaRaf36	*nucl: 9.0,<br>cyto: 4.0*	*Nuclear 1.990 *<br>Chloroplast 1.277<br>Mitochondrial 1.128*	*Nuclear Reliability Index= 4<br>Expected Accuracy=~ 97%*
BnaRaf37	*cyto: 5.0,<br>chlo: 4.0,<br>nucl: 4.0*	*Cytoplasmic 3.421 *<br>Periplasmic 1.024<br>InnerMembrane 0.409<br>OuterMembrane 0.096<br>Extracellular 0.050*	*Cytoplasmic Protein Details<br><br>Reliability Index= 2<br>Expected Accuracy=~ 75%*
BnaRaf39	*cyto: 11.0,<br>nucl: 2.0*	*Cytoplasmic 2.214 *<br>Nuclear 1.422*	*Cytoplasmic Reliability Index= 2<br>Expected Accuracy=~ 75%*
BnaRaf41	*cysk: 7.0,<br>cyto: 4.0,<br>chlo: 1.0,<br>nucl: 1.0*	*Cytoplasmic 3.219 *<br>Periplasmic 0.960<br>InnerMembrane 0.643<br>Extracellular 0.132<br>OuterMembrane 0.047*	*Cytoplasmic Protein Details<br><br>Reliability Index= 3<br>Expected Accuracy=~ 94%*
BnaRaf46	*chlo: 10.0,<br>mito: 3.0*	*Cytoplasmic 4.095 *<br>Periplasmic 0.757<br>OuterMembrane 0.083<br>InnerMembrane 0.035<br>Extracellular 0.030*	*Cytoplasmic Protein Details<br><br>Reliability Index= 1<br>Expected Accuracy=~ 53%*

Subcellular localizations are predicted using two different programs, one is PSORT (http://psort.nibb.ac.jp) and the other is CELLO v2.5 (http://cello.life.nctu.edu.tw) and the third is ESLPred (http://www.imtech.res.in/raghava/eslpred/index.html). nucl-nuclear, cyto-cytoplasm,pp-periplasm, mito-mitochondria,chlo-chloroplast,and pm-plasma membrane.

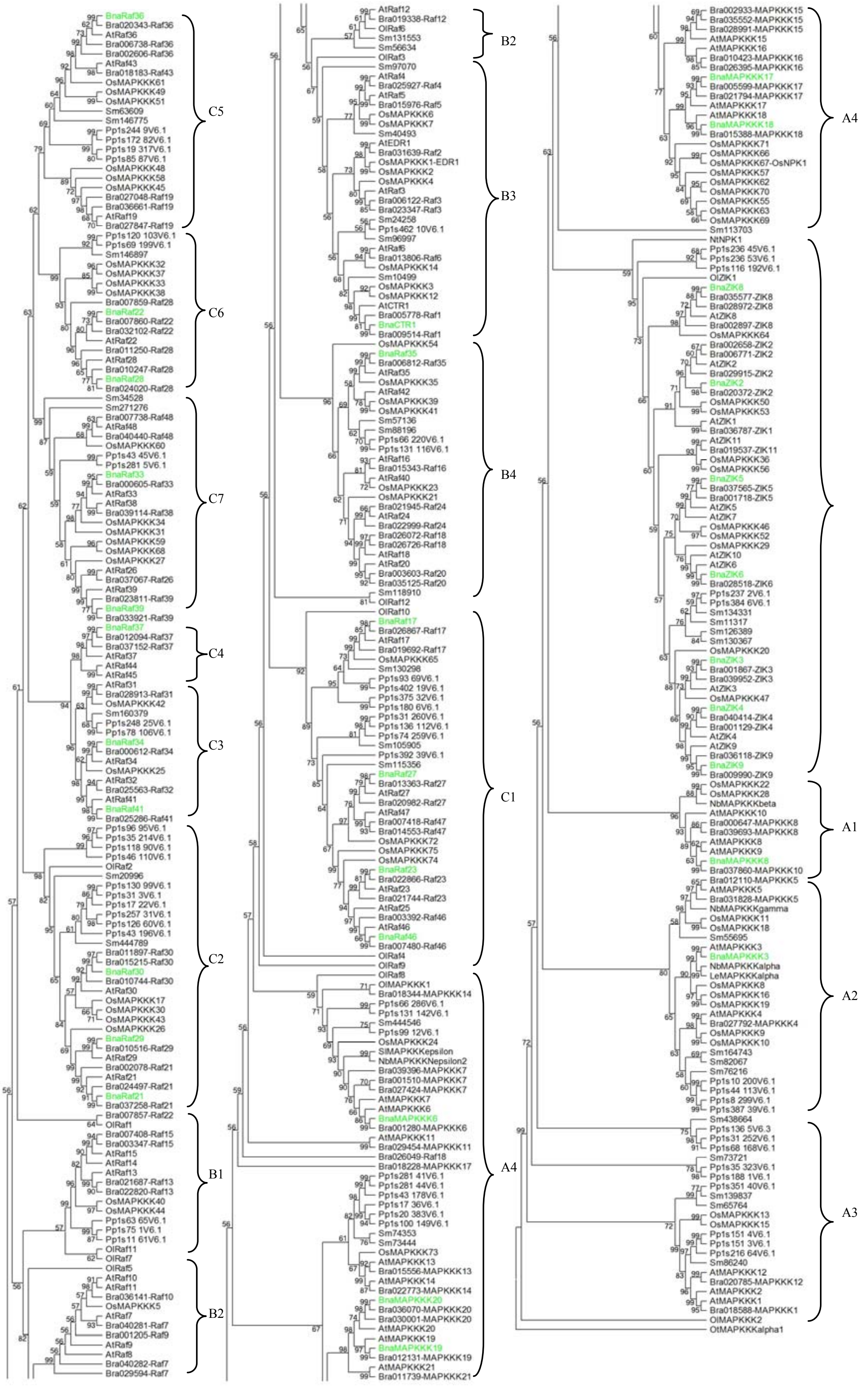

Figure S1 Phylogenetic analysis of MAPKKKs from eight species. The evolutionary relationship was inferred using the maximum parsimony (MP) method implemented in MEGA5.1 program. For clarity

of presentation, the respective MAPKKK proteins are depicted by a two to three-letter code denoting the species in combination with numbers, locus ID or GenBank accession numbers representing the exact MAPKPK from that species. An MAPKKK from the marine green alga *Ostreococcus tauri* (*Ot*) was used to root the tree. The MAPKKK proteins from various species can be divided into three major groups (A to C), as supported by highly significant bootstrap values. The analysis involved 426 amino acid sequences. The numbers on the nodes are percentages from a bootstrap analysis of 500 replicates. There were a total of 5622 positions in the final dataset. The green highlighted proteins are from canola. The numbers on the nodes are percentages from a bootstrap analysis of 500 replicates. *Arabidopsis thaliana (At), Oryza sativa (Os), Brassica napus (Bna), Brassica rapa (Bra), Physcomitrella patens (Pp), Selaginella moellendorffii (Sm), Nicotiana tabacum (Nt), Nicotiana benthamiana (Nb), Solanum lycopersicum (Sl), Lycopersicon esculentum (Le), Ostreococcus lucimarinus (Ol),* and *occus tauri (Ot)* .

A

```
BnaMAPKKK18      1 ------------------------------------------------------------
BnaMAPKKK17      1 ------------------------------------------------------------
BnaMAPKKK19      1 ------------------------------------------------------------
BnaMAPKKK20      1 ------------------------------------------------------------
BnaMAPKKK3       1 ------------------------------------------------------------
NbMAPKKKα        1 ------------------------------------------------------------
BnaMAPKKK8       1 ------------------------------------------------------------
AtMEKK1          1 ------------------------------------------------------------
NbMAPKKKβ        1 ------------------------------------------------------------
NbMAPKKKγ        1 ------------------------------------------------------------
BnaMAPKKK6       1 MARQMTSSQFHKSKTLDNKYMLGDEIGKGAYGRVYIGLDLENGDFVAIKQVSLENIVQED
consensus        1

BnaMAPKKK18      1 ------------------------------------------------------------
BnaMAPKKK17      1 ------------------------------------------------------------
BnaMAPKKK19      1 ------------------------------------------------------------
BnaMAPKKK20      1 ------------------------------------------------------------
BnaMAPKKK3       1 ------------------------------------------------------------
NbMAPKKKα        1 ------------------------------------------------------------
BnaMAPKKK8       1 ------------------------------------------------------------
AtMEKK1          1 ------------------------------------------------------------
NbMAPKKKβ        1 ------------------------------------------------------------
NbMAPKKKγ        1 ------------------------------------------------------------
BnaMAPKKK6      61 LNTIMQEIDLLKNLNHKNIVKYLGSLKTKTHLHIILEYVENGSLANIIKPNKFGPFPESL
consensus       61

BnaMAPKKK18      1 ------------------------------------------------------------
BnaMAPKKK17      1 ------------------------------------------------------------
BnaMAPKKK19      1 ------------------------------------------------------------
BnaMAPKKK20      1 ------------------------------------------------------------
BnaMAPKKK3       1 ------------------------------------------------------------
NbMAPKKKα        1 ------------------------------------------------------------
BnaMAPKKK8       1 ------------------------------------------------------------
AtMEKK1          1 ------------------------------------------------------------
NbMAPKKKβ        1 ------------------------------------------------------------
NbMAPKKKγ        1 ------------------------------------------------------------
BnaMAPKKK6     121 VTVYIAQVLEGLVYLHEQGVIHRDIKGANILTTKEGLVKLADFGVATKLNEADVNTHSVV
consensus      121

BnaMAPKKK18      1 ------------------------------------------------------------
BnaMAPKKK17      1 ------------------------------------------------------------
BnaMAPKKK19      1 ------------------------------------------------------------
BnaMAPKKK20      1 ------------------------------------------------------------
BnaMAPKKK3       1 ------------------------------------------------------------
NbMAPKKKα        1 ------------------------------------------------------------
BnaMAPKKK8       1 ------------------------------------------------------------
AtMEKK1          1 ------------------------------------------------------------
NbMAPKKKβ        1 ------------------------------------------------------------
NbMAPKKKγ        1 ------------------------------------------------------------
BnaMAPKKK6     181 GTPYWMAPEVIEMSGVCAASDIWSVGCTVIELLTCVPPYYDLQPMPALFRIVQDDSPPIP
consensus      181
```

```
BnaMAPKKK18      1 ------------------------------------------------------------
BnaMAPKKK17      1 ------------------------------------------------------------
BnaMAPKKK19      1 ------------------------------------------------------------
BnaMAPKKK20      1 ------------------------------------------------------------
BnaMAPKKK3       1 --------------------------------------MPTWWVRKSSKNKDDSHLLQTQ
NbMAPKKKα        1 --------------------------------------MPAWWGKKSTKNKETQSKEKER
BnaMAPKKK8       1 ---------------MDRIIARMK--KSSGRRG---DKTPTRRLERRDALKHINYDAAST
AtMEKK1          1 ---------------MDRILARMK--KSTGRRGGDKNITPVRRLERRDAARNINYDAASC
NbMAPKKKβ        1 ------------MHRLPGIFAHKKRVDSKQKCAVSSSFKAKPKLERLNARKDIDYEPCML
NbMAPKKKγ        1 ---------------MRWWQNAFSSSTSSSSASSSSSLSSKVHSDDSLITSSGGGGIRYT
BnaMAPKKK6     241 DSLSPDITDFLRQCFKKDSRQRPDAKTLLSHPWIRNSRRALQSSLRHSGTIRYMKGADSS
consensus      241                                              k

BnaMAPKKK18      1 ------------------------------------------------------------
BnaMAPKKK17      1 ------------------------------------------------------------
BnaMAPKKK19      1 ------------------------------------------------------------
BnaMAPKKK20      1 ------------------------------------------------------------
BnaMAPKKK3      23 TRS-------------------VSDKSIRRISADNS-KSSPDPVTPSRCTPRCSREFAGA
NbMAPKKKα       23 DKY-------------------VKPRSFDEVLCRNSPRNSKDLNLGGSGSGFSGSGFSGF
BnaMAPKKK8      41 STWSAEDISAS---LVTRSLELPDRTSFRIFGGGDVEMDQIYKSLGLSGPDDLAISFDAW
AtMEKK1         44 SSSSAEDLSVSTSSLMTRSLEFPEPTSFRIGGG-VGEMDRIYRSLGVSGPDDLAISFDAW
NbMAPKKKβ       49 EFN-------------------GDSKSFRIEGHDSELLDNFFEKLGFSGPDDFAIPAAEW
NbMAPKKKγ       46 LKSAFFFARRRCHARAKKLQNLAENDVDDWRSHYSENENLSSSKCSSSSDRRPPPQPLPL
BnaMAPKKK6     301 SEKDGEGSQDIAESVSAEKVGMSKTNSKSKLGVGSFRSEKDQSSASDIGEERADSEDDIM
consensus      301                           s

BnaMAPKKK18      1 ------------------------------------------------------------
BnaMAPKKK17      1 ------------------------------------------------------------
BnaMAPKKK19      1 ------------------------------------------------------------
BnaMAPKKK20      1 ------------------------------------------------------------
BnaMAPKKK3      63 SGFSD-------------EKKCHPLPLPSLSNDQVNGSVSGSGSVSSVSSSGSGE-----
NbMAPKKKα       64 DSGSS-------------LDKAHPLPRPSVGNDQGVVLGCGSVSVSSTSSSGSSDGPVNT
BnaMAPKKK8      98 EACKKRSSSDVINRFKSFELDLHNFPVPSLSEAAPSYGGMGENKPCLERTPTITVKSRGY
AtMEKK1        103 EACKKRSSSDVVNRFKSFDLDKVRDQDLSEEGPSGVVVGSDSMNHKVQGQDLSEAGPSGG
NbMAPKKKβ       90 DAMQLRSSS---------SSEVIGLGNSKIKDNIFEYNDIPIKSRDVVQLQISDASKADT
NbMAPKKKγ      106 PELQLLLRHDADATFSNEPIVPLPSPTDAHNSHHKTGEERVKDTHKDGVAHGSRLSSQDA
BnaMAPKKK6     361 SDQGPTLSIHDNKSSLQSSTCSISSDAKGTSQDGKSEPDGNLEMEASEGRRKASATKQVG
consensus      361

BnaMAPKKK18      1 ------------------------------------------------------------
BnaMAPKKK17      1 ------------------------------------------------------------
BnaMAPKKK19      1 ------------------------------------------------------------
BnaMAPKKK20      1 ------------------------------------------------------------
BnaMAPKKK3     105 -DQSQPTAPR------------------------------------------------KS
NbMAPKKKα      111 TDQAQFDILF------------------------------------------------RG
BnaMAPKKK8     158 LVPNHGDVAS---------GGGAGEDKR---GLVRTPTITVKSRGYLVPN-------QSD
AtMEKK1        163 IVTELSEIGNLITPVDRLVADGVVENRR---VMERTPTI-VKSKGYLVPNNVVAVGVGVG
NbMAPKKKβ      141 IRLEDCVTGN-----------GAVLNGNEPVTLVRCGG-------------------GGG
NbMAPKKKγ      166 RKKKKEHLGSRLSRKTPQKLHVTDRVSDIRISVPISAPTTPYASPGLSPLKAEDFLSPNC
BnaMAPKKK6     421 KESSIQMQQRSHSFGPKGEDRGLRKAVKTPSSYGGNELTRFSDPPGDACLHDLFHPLNKV
consensus      421
```

```
BnaMAPKKK18     1 ------------------------------------------------------------
BnaMAPKKK17     1 ------------------------------------------------------------
BnaMAPKKK19     1 ------------------------------------------------------------
BnaMAPKKK20     1 ------------------------------------------------------------
BnaMAPKKK3    116 N---------AAAASPKAGARPTSPLHNRFSGMTLESSSTGRNDDGRSSEYHPLP-----
NbMAPKKKα     123 NGDNRLSPLSRSPVRSRGTTTTSSPLHPRFSSMNLDSP-TGKLDNDVSSESHQLP-----
BnaMAPKKK8    199 VGIKGVRPPVLKPPPAK-RPPIDHQ-GSSWDFLTHFAPEDGIVRRPSSSSTSDNG-----
AtMEKK1       219 GGIKGLRPPVLKPPPAMKRPPIDHR-GSSWDFLTHFAPSETVKRPSSSSSSSEDGCDEEE
NbMAPKKKβ     171 GGIKGVRPPLLAPPPVMSLPIVDDSSCSTWDIFRAFGPKD-QRESGLCRSEVVNGDAECV
NbMAPKKKγ     226 MAFPGAFQVCSAPEMPPSDTPHYPDFSYKVIPEKNAFSVDNSPHQSPRTSPQRCRKMASG
BnaMAPKKK6    481 PEGKLNEASASTPASNANQGDSPVADGGKNDLATKLRARIAQKQMEGETGHSNDGGDLFR
consensus     481             p                                 s

BnaMAPKKK18     1 ---------------------------------------------------------MGN
BnaMAPKKK17     1 ----------------------------------------------------------ME
BnaMAPKKK19     1 ----------------------------------------------------------ME
BnaMAPKKK20     1 ----------------------------------------------------------ME
BnaMAPKKK3    162 ---------------------LPPGSPTSPSVVLPCSPTSPSSGVQGSWVVGGSEKEISK
NbMAPKKKα     177 ---------------------LPPGSPPSPSALPNPRTCG---------VAEGSNVNMSK
BnaMAPKKK8    252 ---EETLEEEV--------DTGDEACSFSTNEGGGDSSSTVSN-TSPIYANG--GSIITS
AtMEKK1       278 GKEEEAEAEEMGARFIQLGDTADETCSFTTNEG--DSSSTVSN-TSPIYPDG--GAIITS
NbMAPKKKβ     230 KNEVDEEENSTSRRRIGVSNLLSESCSFTTTSNDDDSSSSTTERMSSISPNGRFARYITY
NbMAPKKKγ     286 STSPLHNLLPNESSAARRECSAQGNVHPLPLPPLGATPSQQPTAIPPVSPYAELTPIKGQ
BnaMAPKKK6    541 LMMGVLKDDVIDIDGLVFDEKASPDNLLPLQAVEFSRLVSSLRPSETEDAIVTSCQKLVA
consensus     541                                                          m
```

Motif 1

Serine/Threonine protein kinases active-site signature

(L/I)GXGX(F/Y/T/S)(S/G/A)XVXX(G/A)X(5-18)AVK

```
BnaMAPKKK18     4 WTREKTIGRGSSATVYAATCQDS------GETIAVKSAEFHQSEFLQREAKLLSSLN--S
BnaMAPKKK17     3 WTRGRILGRGSTATVCAATRHNS------NEILAVKSSELHRSEFLQREAEILSSLS--S
BnaMAPKKK19     3 WVRGETIGHGTFSTVSLATPTNN-AYGAFPQVMAVKSADSHGAASLANEKWVLDILGNDC
BnaMAPKKK20     3 WVRGETIGYGTFSTVSLATPSNTKGSGEYPSLMAVKSSNSYGG--IANEKSVLDSLG-DC
BnaMAPKKK3    201 WKKGRFIGSGTFGKVYQGFNSEEGRICAIKEVKVISDDKNSKECLKQLNQEINVLSQLCH
NbMAPKKKα     207 WKKGKLLGRGTFGHVYLGFNRENGQMCAIKEVRVVSDDQTSKECLKQLNQEIILLSNLSH
BnaMAPKKK8    298 WQKGGLLGRGSFGSVFEGIS-GDGDFFAVKEVSLLEQGSQAQECIQQLEGEIALLSQLQH
AtMEKK1       333 WQKGQLLGRGSFGSVYEGIS-GDGDFFAVKEVSLLDQGSQAQECIQQLEGEIKLLSQLQH
NbMAPKKKβ     290 WDKGDLLGRGSFGSVYEGIS-DDGFFFAVKEVSLLDQGDGGRQSLYQLEQEIELLSQFEH
NbMAPKKKγ     346 WQKGKLIGRGTFGSVYVASNRETGALCAMKEVELLPDDPKSAESIRQLQQEINVLSQLKH
BnaMAPKKK6    601 MFRHRPEQKVVFVTQHGFLPVMDLLDSPKSRVTCAVLQLINEIIKDNIDFQENACLVGLI
consensus     601 w kgkiigrgsfgtvy a     g   aikev vv          l qle ei vlsn  h

BnaMAPKKK18    56 PYVIGYRGCEVTKEP--LATYNLLMEYAPYGTLADVAAKNGG-CIDEAR---------VV
BnaMAPKKK17    55 PYVIGYRGSETKRELNGVVTYNLLMEYAPYGTLTDAAAKNGG-GLEEAM---------IV
BnaMAPKKK19    62 DGLVRCLGEDRTVEN-GEEMHNLFLEYASRGSLASHLKKLNGEGLPEST---------VR
BnaMAPKKK20    60 PEIVRCFGEDQTVEK-GEKLHNLLLEYASRGSLASQLKKLNGEGLPETT---------VR
BnaMAPKKK3    261 PNIVQYYGSELSEET-----LSVYLEFVSGGSIYKLLTEYGAFTEPV-----------IQ
NbMAPKKKα     267 PNIVRYYGSELDDET-----LSVYLEYVSGGSIHKLLQEYGAFREPV-----------IQ
BnaMAPKKK8    357 QNIVRYRGTAKDGSN-----LYIFLELVTQGSLSKLYQRY-QLMDSV-----------VS
AtMEKK1       392 QNIVRYRGTAKDGSN-----LYIFLELVTQGSLLKLYQRY-QLRDSV-----------VS
NbMAPKKKβ     349 ENIVRYYGTDKDDSK-----LYIFLELVTQGSLLSLYQKY-HLRDSQ-----------VS
NbMAPKKKγ     406 PNIVQYYGSEIVGDR-----FYIYLEYVHPGSINKFIHDHCEAITES-I---------VR
BnaMAPKKK6    661 PLVMSFAGPERDRSREIRKEAAYFLQQLCQSSSLTLQMFIACRGIPVLVGFLEADYAKYR
consensus     661 pnivry Gse   e        lfleyvs gsl  l  k  g                v
```

Motif 2 **Motif 3**

Protein kinases ATP-binding region signature

(L/I/V/M/F/Y/C)XHXD(L/I/V)KXXN(L/I/V/M)XX

(R/K)X{2,3}(D/E)X(2,3)Y

```
BnaMAPKKK18   104 SYTRQILLGLEYVHNSKRIAHCD------------VKGSNVLVGVNGEAKIADFGCAKR-
BnaMAPKKK17   105 KYTREMLRGLEYIH-SRGVAHCD------------VKGSNVVVGEKGEAKIVDFGCAKR-
BnaMAPKKK19   112 RHTGSVLRGLRHIH-AKGFAHCD------------LKLGNILLFGDGAVKIADFGLAKRA
BnaMAPKKK20   110 RHTGSVLRGLRRIH-SRGFAHCD------------IKLENILLFGDGAVKIADFGLAR--
BnaMAPKKK3    305 NYTRQILYGLAYLH-GRNTVHRD------------IKGANILVDPNGEIKLADFGMAKH-
NbMAPKKKα     311 NYTRQILSGLSFLH-ARNTVHRD------------IKGANILVDPNGEIKLADFGMAKH-
BnaMAPKKK8    400 TYTRQILDGLKYLH-DKGFIHRD------------IKCANILVDANGAVKLADFGLAKV-
AtMEKK1       435 LYTRQILDGLKYLH-DKGFIHRD------------IKCANILVDANGAVKLADFGLAKV-
NbMAPKKKβ     392 VYTRQILHGLKYLH-DRNVVHRD------------IKCANILVDANGSVKLADFGLAKA-
NbMAPKKKγ     451 NFTRHILCGLAYLH-SKKTIHRD------------IKGANLLVDAYGVVKLADFGMAKH-
BnaMAPKKK6    721 EMVHLAIDGMWQVFKLKRSTPRNDFCRIAAKNGILLRLINTLYSLNEATLLASEGRSGQ-
consensus     721  ytrqil Gl ylh  k  vhrd            ik aNilvdgng vkladfGlakr
```

Motif 4

G(T/S)PX(F/Y/W)MAPEV

```
BnaMAPKKK18   151 ---VEPELT-EPVRGTPAFMAPEVARGER-------QGKESDIWALGCTVIEMVTGSHPW
BnaMAPKKK17   151 ---VDPEVESEPVVGTPAFMAPEVARGEK-------QGRESDIWAVGCTVIEMATGSPPW
BnaMAPKKK19   159 GEVTALMDGGVQIRGTPLYMAPESVNDNE-------YGSEADVWALGCAVVEMFSGKTAW
BnaMAPKKK20   155 -DLTASKEG-VEIRGTPLYMAPESVNYNE-------YGAAADVWALGCAVVEMFSGETAW
BnaMAPKKK3    351 ---VTAYSTMLSFTGSPYWMAPEVVMHKN------GYTLAVDVWSVGCTILEMATAKPPW
NbMAPKKKα     357 ---ITSSSLVLSFKGSPYWMAPEVVMNTS------GYGLPVDIWSLGCAILEMASSKPPW
BnaMAPKKK8    446 ---SKLN-DIKSCKGTPFWMAPEVINPKRT----DGYGSSADIWSLGCTVLEMLTGQIPY
AtMEKK1       481 ---SKFN-DIKSCKGTPFWMAPEVINRKDS----DGYGSPADIWSLGCTVLEMCTGQIPY
NbMAPKKKβ     438 ---TKLN-DVKSCKGTALWMAPEVVNRKN-----QGYGQAADIWSLGCTVLEMLTRQFPY
NbMAPKKKγ     497 ---LNGQAANLSLKGSPYWMAPELLQSVMQKDSNSDLAFATDIWSLGCTVIEMLNGKPPW
BnaMAPKKK6    780 ---LDQHEALLSVIDHPDVLKTRPGGGEEP-----SNSQRSDLYQPDGDRPRSSSAALDA
consensus     781    v     mvsvrgtp wmapevvn          yg   Diwslgctviem tg  pw
```

```
BnaMAPKKK18   200 TGGDTTDP-VSVLYRVGYMGETPELPSSLS--------EQAKDFLGKCLRREAKERWTAR
BnaMAPKKK17   201 TEASSGESSVSVLYRVGYSGEAPELPCLLT--------EEAKDFLEKCFKREAKERWTAT
BnaMAPKKK19   212 SFKE-GSHFMSLLIRIGVGDELPRIPEDLS--------EQGRDFLSKCFVKDPKKRWTAE
BnaMAPKKK20   206 SVEE-GSHFMSFLLRIGVGEELPRVPEELS--------EEGKDFLSKCFVKDPEKRWTVE
BnaMAPKKK3    402 SQFEGVAAIFKIGNSK----DMPEIPDHLS--------NDAKNFIRLCLQRNPTVRPTAA
NbMAPKKKα     408 SQYEGVAAIFKIGNSK----DFPEIPDHLS--------NDAKNFIKLCLQREPSARPTAS
BnaMAPKKK8    498 CDLENPVQALYRIGRG----VLPDIPDTLS--------LDGRDFITECLKVDPEERPTAA
AtMEKK1       533 SDLE-PVQALFRIGRG----TLPEVPDTLS--------LDARLFILKCLKVNPEERPTAA
NbMAPKKKβ     489 SHLENQMQALFRIGKG----EPPPVPNTLS--------IDARNFINQCLQVDPSARPTAS
NbMAPKKKγ     554 SEYEAAAAMFKVLKDT------PPIPETLS--------PEGKDFLRWCFCRNPAERPSAS
BnaMAPKKK6    832 TEDVKQHHRISISSNRTSTDKIQKLAESASNGYAVTQPEQVRPLLSLLEKETPSRHVSGQ
consensus     841 s  e      v vl  r      elpelpesls        eakdfl   cl rep  rpta
```

```
BnaMAPKKK18   251 QLLNHPFVTTKPNTEPEFVTGLVTNSPTSVTDQMFWRSVEEEE----------DQERPSW
BnaMAPKKK17   253 QLLNHPFLITKPNTEP--VSGLVSSSPTSVIDQTFWTSGGEEEETEELQEDSRNLDRLNL
BnaMAPKKK19   263 MLLNHPFVAVDLDQDDLEGVD-IQLK-TEEVFASPRCPFEFSS--------WVSASCDSG
BnaMAPKKK20   257 MLLNHPFVAVDCEDDDYDQRDELCLKGKDKVSKSPRCPFGFPD--------WESMSSESE
BnaMAPKKK3    450 QLLEHPFLRVHSP-RVANTSMHKDVPPRPYDGSSSMPTREP------PYSG---RHPVFH
NbMAPKKKα     456 QLLEHPFVKNQSTTKVTHVGVTKEAYPRSFDGNRTPPVLDSGGRNISPTKGNYASHPVIT
BnaMAPKKK8    546 ELLNHPFVRRPLAYSGSG-LGSASPHVHRRG-----------------------------
AtMEKK1       580 ELLNHPFVRRPLPSVGSGGSGSASPLLRR-------------------------------
NbMAPKKKβ     537 QLLEHPFVKRTLPS--SSGSASPQNLGKRL------------------------------
NbMAPKKKγ     600 MLLEHRFMRISHQPDVPSFLKPVGGVRIKEKLNSQK------------------EQTTYN
BnaMAPKKK6    892 LDYVKHIAGLEKHESILPLLRASIDTMPRYFSKTMSKKVMAIEGAASASGVLSGSGVLNA
consensus     901  llnhpfvr                    k     t
```

```
BnaMAPKKK18     301 WECHEERIGGLSWIGQAVVDPTWDMGGEDWITVRRNN-----------------------
BnaMAPKKK17     311 WACHSERIGRLRCVG-GLDGSRLDMEGGGWITVRVSCEGTMIGGSHEENILEVGGTVN--
BnaMAPKKK19     313 TQYHTLEAPEERVATLAAGSIPDWSVTNNWVTVR--------------------------
BnaMAPKKK20     309 IQYQPLDSPVERFASLVSESIPNWSVSEGWVTVR--------------------------
BnaMAPKKK3      500 PIK--SPSRENVRAITSLPVSPCSSPLRQLGPAYKSCFLSPPHPSYAFPVQE-SGYNHQA
NbMAPKKKα       516 ISRPLSCPREIVKTITSLPVSPTSSPLRQYEPARRSCYLSPPHPAYGIGGHSGYDENDYL
BnaMAPKKK8          ------------------------------------------------------------
AtMEKK1             ------------------------------------------------------------
NbMAPKKKβ           ------------------------------------------------------------
NbMAPKKKγ       642 LDQGRLSLER--------------------------------------------------
BnaMAPKKK6      952 RLGSDTSSGLLSHMVTTLSAEVASQYLEKVADLLLEFARADTTVKSYMCSQSLLSRLFHM
consensus       961


BnaMAPKKK18         ------------------------------------------------------------
BnaMAPKKK17         ------------------------------------------------------------
BnaMAPKKK19         ------------------------------------------------------------
BnaMAPKKK20         ------------------------------------------------------------
BnaMAPKKK3      557 EFAASPFRFKKDTSLIEPSSYRAQAPSSPFRSRLV-------------------------
NbMAPKKKα       576 MFQARPTTRNTLEPWLEIPQFKVQTPSRSPKLRPIL------------------------
BnaMAPKKK8          ------------------------------------------------------------
AtMEKK1             ------------------------------------------------------------
NbMAPKKKβ           ------------------------------------------------------------
NbMAPKKKγ           ------------------------------------------------------------
BnaMAPKKK6     1012 FNRVEPPILLKILKCTNHLSTDPNCLESLQRADAIKHLIPNLEVKEGNLVDQIHHEVLSA
consensus      1021


BnaMAPKKK18         ------------------------------------------------------------
BnaMAPKKK17         ------------------------------------------------------------
BnaMAPKKK19         ------------------------------------------------------------
BnaMAPKKK20         ------------------------------------------------------------
BnaMAPKKK3          ------------------------------------------------------------
NbMAPKKKα           ------------------------------------------------------------
BnaMAPKKK8          ------------------------------------------------------------
AtMEKK1             ------------------------------------------------------------
NbMAPKKKβ           ------------------------------------------------------------
NbMAPKKKγ           ------------------------------------------------------------
BnaMAPKKK6     1072 LFNLCKINKRRQEQAAENGIIPHLMLFVMSDSPLKQYALPLLCDMAHASRNSREQLRSHG
consensus      1081


BnaMAPKKK18         ------------------------------------------------------------
BnaMAPKKK17         ------------------------------------------------------------
BnaMAPKKK19         ------------------------------------------------------------
BnaMAPKKK20         ------------------------------------------------------------
BnaMAPKKK3          ------------------------------------------------------------
NbMAPKKKα           ------------------------------------------------------------
BnaMAPKKK8          ------------------------------------------------------------
AtMEKK1             ------------------------------------------------------------
NbMAPKKKβ           ------------------------------------------------------------
NbMAPKKKγ           ------------------------------------------------------------
BnaMAPKKK6     1132 GLDVYLSLLDDEYWSVIALDSIAVCLAQDNDNRKVEQALLKDDAIYTLVNFFQSCPERHF
consensus      1141
```

```
BnaMAPKKK18        ------------------------------------------------------------
BnaMAPKKK17        ------------------------------------------------------------
BnaMAPKKK19        ------------------------------------------------------------
BnaMAPKKK20        ------------------------------------------------------------
BnaMAPKKK3         ------------------------------------------------------------
NbMAPKKKα          ------------------------------------------------------------
BnaMAPKKK8         ------------------------------------------------------------
AtMEKK1            ------------------------------------------------------------
NbMAPKKKβ          ------------------------------------------------------------
NbMAPKKKγ          ------------------------------------------------------------
BnaMAPKKK6    1192 VHILEPFLKIITKSSRINTTLAVNGLTPLLIARLDHQDAIARLNLLKLIKAVYEHHPRPK
consensus     1201

BnaMAPKKK18        ------------------------------------------------
BnaMAPKKK17        ------------------------------------------------
BnaMAPKKK19        ------------------------------------------------
BnaMAPKKK20        ------------------------------------------------
BnaMAPKKK3         ------------------------------------------------
NbMAPKKKα          ------------------------------------------------
BnaMAPKKK8         ------------------------------------------------
AtMEKK1            ------------------------------------------------
NbMAPKKKβ          ------------------------------------------------
NbMAPKKKγ          ------------------------------------------------
BnaMAPKKK6    1252 QLIVENDLPQRLQNLIEERREGQHLGGQVLVKQMATSLLKALHINTVL
consensus     1261
```

B

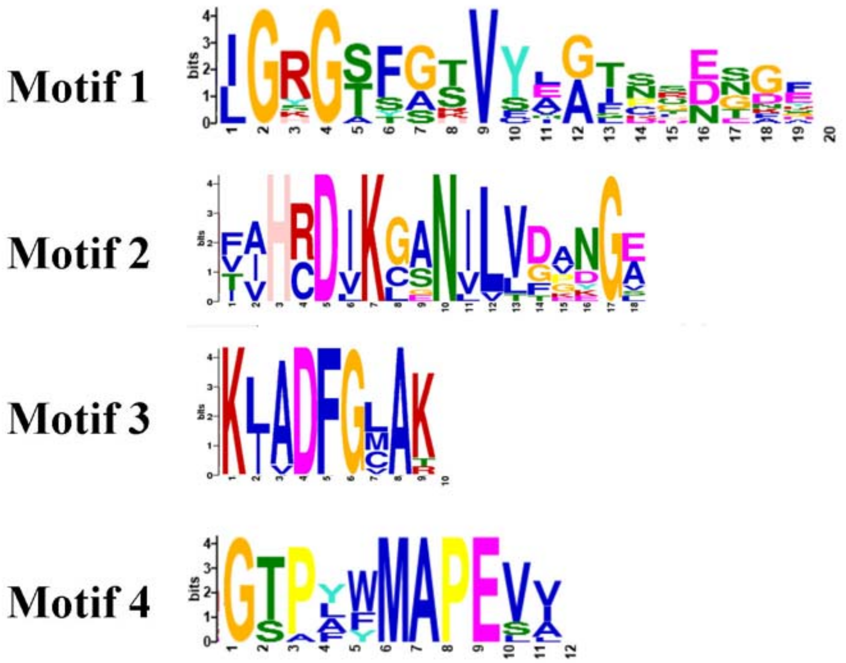

Figure S2. Multiple alignment of MEKK-subfamily MAPKKK proteins in representative species and schematic diagram of four motifs of BnaMAPKKKs. (A) Multiple alignment of MEKK-subfamily MAPKKK proteins in representative species. Multiple sequence alignment was performed using the ClustalX1.83 and illustrated by BOXSHADE (http://www.ch.embnet.org/software/BOX_form.html). Identical amino acids are shaded in black,

and similar amino acids are shaded in gray. Four motifs were shown as overbars, which are motif 1 of Serine/Threonine protein kinases active-site signature, motif 2 of Protein kinases ATP-binding region signature, motif 3 of Tyrosine kinase phosphorylation site and motif 4 of G(T/S)PX(F/Y/W)MAPEV. (B) Schematic diagram of four motifs of BnaMAPKKKs. Motif analysis was determined by using MEME4.0 program as described in Materials and Methods section.

A

Motif 1

Protein kinases ATP-binding region signature

(L/I)GXG(A/S)(F/V/S)KXVXX(G/A)X(5-18)AVK

I II

```
BnaZIK4       1 ----MDNNLVSYLEPDYSEFVEVDPTGRYGRYNEVLGKGASKTVYRAFDEYEGIEVAWNQ
AtZIK4        1 ----MNN--LSYLEPDYSEFVEVDPTGRYGRYNEVLGKGASKTVYRAFDEYEGIEVAWNQ
BnaZIK9       1 ---MMNN--ISHLESDDSEFVEVDPSGRYGRYNEVLGKGSSKTVYRGFDEYEGIEVAWNQ
AtZIK9        1 ---MMNN--LSHLESDYSEYVEVDPTGRYGRYNEVLGKGSSKTVYRGFDEYQGIEVAWNQ
OsMAPKKK20    1 ---MMGPKANAAAAGDLPEYAEVDPTGRYGRYNDVLGKGASKTVYRAFDEYQGMEVAWNQ
BnaZIK3       1 ---MGNG--EESFVEDCSEYVEIDPSGRYGRYDEVLGKGASKTVYKAFDEYEGIEVAWNQ
AtZIK3        1 ----MNG--EESFVEDCSVFVEIDPSGRYGRYDEILGKGASKTVYRAFDEYEGIEVAWNQ
BnaZIK2       1 ----------MVNMNQLSEYVETDPTGRYGRFEEVLGRGAMKTVFKAIDEKLGIEVAWSQ
BnaZIK8       1 MVLPCASSESESLDKDSEPFVETDPTGRYGRYDELLGSGAVKKVYRAFDQEEGIEVAWNQ
BnaZIK6       1 -MAFGS----SGTSEEEADFADKDPTGRYIRYHDVLGRGAFKTVYKAFDEVDGIEVAWNL
AtZIK6        1 -MASGSGFLGQISSMEEADFAEKDPSGRYIRYDDVLGRGAFKTVYKAFDEVDGIEVAWNL
AtZIK10       1 ---------------MEEADFVQKDPTGRYIRYNDVLGRGAFKTVYKAFDEVEGIEVAWNL
BnaZIK5       1 --MDGAEDAAPIHEPPDPEVLEVDPTLRYIRYKEVIGKGAFKTVYKAFDEVDGIEVAWNQ
consensus     1                d sefvevDPtgRYgRy evlGkGasKtVyrafDeyeGiEVAWnq

BnaZIK4      57 VKLYDFLQSPEDLERLYCEIHLLKTLKHKNIMKFYTSWVDTSNRNINFVTELFTSGTLRQ
AtZIK4       55 VKLYDFLQSPEDLERLYCEIHLLKTLKHKNIMKFYTSWVDTANRNINFVTELFTSGTLRQ
BnaZIK9      56 VKLYDFLQSPQELERLYCEIHLLKTLKHKSIMKFYTSWVDTDNRNINFITEMFTSGTLRQ
AtZIK9       56 VKLYDFLQSPQELERLYCEIHLLKTLKHKSIMKFYASWVDTDNRNINFVTEMFTSGTLRQ
OsMAPKKK20   58 VKLHDFLQSPEDLERLYCEIHLLKTLKHRNIMKFYTSWVDVSRRNINFITEMFTSGTLRQ
BnaZIK3      56 VKLRNFTRNPEELEKFFREIHLLKTLNHQNIMKFYTSWVDTNNLAINFVTELFTSGTLRQ
AtZIK3       55 VKLRNFTRNPEELEKFFREIHLLKTLNHQNIMKFYTSWVDTNNLSINFVTELFTSGTLRQ
BnaZIK2      51 VKLKEVLRSSVDLQRLYSEVHLLSTLNHKSIIRFYTSWIDVDSHTLNFITELFTSGTLRQ
BnaZIK8      61 VKLRCFSDDAAMLERLYSEVRLLKSLKNTNIIALYKVWRDERSNTLNFITEICTSGNLRE
BnaZIK6      56 VSIEDVMQMPGQLERLYSEVHLLKALKHENIIKLFNSWVDEKNKTINMITELFTSGSLRL
AtZIK6       60 VSIEDVMQMPGQLERLYSEVHLLKALKHENIIKLFYSWVDEKNKTINMITELFTSGSLRV
AtZIK10      47 MSIEDVLQMPGQLDRLYSEVHLLNSLKHDNIIKLFYSWVDDHNKSINMITELFTSGSLTL
BnaZIK5      59 VRIDDVLQSPNSLERLYSEVRLLKSLKHSNIIRFYNSWIDDKNKTVNIITELFTSGSLRH
consensus    61 vkl dflqsp dLerly EihLLktLkh nImkfytsWvD  nrsiNfiTElfTSGtLrq
```

Motif 2 **Motif 3**

Serine/Threonine protein kinases active-site signature

[LIVMFYC]X[HY]XD[LIVMFY]KXXN[LIVMFYCT]XXX

```
BnaZIK4     117 YRLRHKRVNIRAVKHWCRQILRGLHYLHNHDPPIIHRDLKCDNIFVNGNQGEVKIGDLGL
AtZIK4      115 YRLRHKRVNIRAMKHWCRQILRGLHYLHSHDPPVIHRDLKCDNIFVNGNQGEVKIGDLGL
BnaZIK9     116 YRLKHKRVNIRAVKHWCRQILRGLDYLHTHDPPVIHRDLKCDNIFVNGNQGEVKIGDLGL
AtZIK9      116 YRLKHKRVNIRAVKNWCRQILRGLNYLHTHDPPVIHRDLKCDNIFINGNQGEVKIGDLGL
OsMAPKKK20  118 YRQKHMRVNIWAVKHWCRQILSGLLYLHSHDPPIIHRDLKCDNIFVNGNQGEVKIGDLGL
BnaZIK3     116 YRLRHRRVNIKAVKQWCKQILKGLLYLHSCSPPIIHRDLKCDNIFINGNQGEVKIGDLGL
AtZIK3      115 YRLRHRRVNIRAVKQWCKQILKGLLYLHSRSPPIIHRDLKCDNIFINGNQGEVKIGDLGL
BnaZIK2     111 YKNKYMRIDIRAIKSWARQILEGLVYLHEHDPPVIHRDLKCDNIFVNGHLGQVKIGDLGL
BnaZIK8     121 YRKKHRHVSMRALKKWSKQILKGLVYLHTHDPCIIHRDLNCSNVFVNGNIGQVKIGDLGL
BnaZIK6     116 YRKKHRKVDPKAIKNWARQILKGLTYLHSQKPPVIHRDLKCDNIFVNGNTGEIKIGDLGL
AtZIK6      120 YRKKHRKVDPKAIKNWARQILKGLNYLHSQNPPVIHRDLKCDNIFVNGNTGEVKIGDLGL
AtZIK10     107 YRKKHRKVDPKAIMNWARQILKGLHYLHSQTPPVIHRDLKCDNIFVNGNTGKVKIGDLGL
BnaZIK5     119 YRKKHRKVNMKAVKCWARQILMGLRYLHTQVPPIIHRDLKCDNIFINGNHGEVKIGDLGL
consensus   121 Yr khrrvnirAvk WcrQILrGL YLHshdPpvIHRDLkCdNiFvNGnqGevKIGDLGL
```

Motif 3 **Motif 4**

Tyrosine kinase phosphorylation site

[R/K]X{2,3}[D/E]X{2,3}Y **GTPEFMAPE(L/V/M)(Y/F/L)**

```
BnaZIK4      177 AAILRKSHAAHCVG--------------------------------TPEFMAPEVYEEAY
AtZIK4       175 AAILRKSHAAHCVG--------------------------------TPEFMAPEVYEEAY
BnaZIK9      176 AAMLQHSHAAHCVG--------------------------------TPEFMAPEVYAEEY
AtZIK9       176 AACLQHSHAAHCVG--------------------------------TPEFMAPEVYKEEY
OsMAPKKK20   178 AAILRKSHAVHCVG--------------------------------TPEFMAPEVYEEEY
BnaZIK3      176 AAILRKSHAVRCVG--------------------------------TPEFMAPEVYDEEY
AtZIK3       175 AAILRKSHAVRCVGTSKPSHHWNFIALIMFFTTLDLPLLCLCVVKGTPEFMAPEVYDEEY
BnaZIK2      171 ARMLRDCRSAHSVLG-------------------------------TPEFMAPELFEGNY
BnaZIK8      181 AAVVGKNHLAHSILG-------------------------------TPEFMAPELYEEKY
BnaZIK6      176 ATVLQQPTARSVIG--------------------------------TPEFMAPELYEEEY
AtZIK6       180 ATVLQQPTARSVIG--------------------------------TPEFMAPELYEEEY
AtZIK10      167 AAVMQQPTARSVIG--------------------------------TPEFMAPELYEEEY
BnaZIK5      179 ATVMEQAHAKSVIG--------------------------------TPEFMAPELYDEIY
consensus    181 Aail ksha hcvg                                TPEFMAPEvyeeeY

BnaZIK4      205 NELVDIYSFGMCILEMVTFDYPYSECTHPAQIYKKVMSGKKPDALYMVKDPEVKYFIEKC
AtZIK4       203 NELVDIYSFGMCILEMVTFDYPYSECTHPAQIYKKVMSGKKPDALYKVKDPEVKCFIEKC
BnaZIK9      204 NELVDIYSFGMCVLEMVTFDYPYSECTHPAQIYKRVISGKKPDSLDKVKDSEVRGFIEKC
AtZIK9       204 NQLVDIYSFGMCVLEMVTFDYPYSECSHPAQIYKRVISGKKPDGLDKVKDPEVRGFIEKC
OsMAPKKK20   206 NELVDIYSFGMCVLEMVTFEYPYSECTHPVQIYKKVISGTKPEALYKVKDPMVRQFVEKC
BnaZIK3      204 NELVDVYAFGMCVLEMVTFDYPYSECTHPAQIYKKVTSGKKPEAFYLVKDPEVREFVEKC
AtZIK3       235 NELVDVYAFGMCVLEMVTFDYPYSECTHPAQIYKKVTSGKKPEAFYLVKDPEVREFVEKC
BnaZIK2      200 NELIDVYSFGMCFLEMITSEFPYSECNSPVHIYKKVSSGKLPSAFYRVEDIEAQIFIRKC
BnaZIK8      210 TELIDVYSYGMCVLELVSLEIPYSECDSVAKIYRKVSSGVKPEALNKVKDLEAKAFIEKC
BnaZIK6      204 TELVDIYSFGMCMLEMVTCEYPYNECRNQAQIYKKVTSGIKPQSLSRVDDPQVRQFIEKC
AtZIK6       208 NELVDIYSFGMCMLEMVTCEYPYNECRNQAQIYKKVTSNIKPQSLGKVDDPQVRQFIEKC
AtZIK10      195 NELVDIYSFGMCMLEMVTCEYPYRECRNQAQIYKKVTSGIKPQSLSKVDDPQVKQFIEKC
BnaZIK5      207 TELADIYSFGMCMLEMVTFEYPYCECRNSAQIYKKVSSGIKPASLSRVQDPEVKQFIEKC
consensus    241 neLvDiYsfGMCiLEmvtfeyPYsECthpaqIYkkVtSgkkPdal kVkDpevr FieKC

BnaZIK4      265 LATVSLRVSARELLDDPFFRIDDGEFDLRSVDVDYPVMPPLVRQPHHHLADYYNYPSNSS
AtZIK4       263 LATVSLRVSARELLDDPFLRIDDGEFDLRSVDMEDSVG-PLYRQP-HHLPDYYNYPSNSS
BnaZIK9      264 LASASLRLSARELLDDQFLCID--------------------------------YPSNYY
AtZIK9       264 LATVSLRLSACELLDDHFLCIDES--DMRRVESEKGLI-DEAGTPLRHSYHIPHYSNGYY
OsMAPKKK20   266 LATASRRLSARELLKDPFLQVDDLVFCPG--DGDYSLMNYLRQPYLEHAYSNVSMMSNGL
BnaZIK3      264 LATVTSRLTALELLQDPFLQDD----------------------VDEFDMRPIDYYNGYD
AtZIK3       295 LANVTCRLTALELLQDPFLQDDN---------------------MDGFVMRPIDYYNGYD
BnaZIK2      260 LVPASQRVSARELLQDPFLSSDE---------------------------SWMVYARGAG
BnaZIK8      270 IAPKKLRPSAAELLRDPFFDGIVD------------------------------------
BnaZIK6      264 LLPAESRPKALELSMDPFLARDGS----------------KDSAPLLASSSTAS------
AtZIK6       268 LLPASSRPTALELSKDPFLARDGG----------------KDSALLASSSTSSKYV----
AtZIK10      255 LLPAPSRPTALELLKDQLLAVDGA----------------KDSTLTASSNTTFKPA----
BnaZIK5      267 LLPASERLSAKELLLDPFLQVNGL----------------TMNNPLPLPDIVMPKEGAFG
consensus    301 la as RvsA ELl Dpfl id                                 y

BnaZIK4      325 SLNRQYSNG-------------------YHEYQNRWAYNPGETEETHGIELFECRNEN--
AtZIK4       321 SLNRQYSNGNYPSNSSSLNRQYSNGYNSHHEYQNGWAYNPAETEETHGIELFESRNND--
BnaZIK9      292 SDN----------------------------DQSQWTYNGDEMIESHGIEIFEFQNEDDH
AtZIK9       321 SLY----------------------------NQNQWDYNGDETVESHEIDLLEFQNDD--
OsMAPKKK20   324 SESIDEDTP----------------------TEDRWDCE-DDDIKADGIDLFN-------
BnaZIK3      302 E---FLRQP---------------------LIDPPLYHDES---QICEIDLFAQ------
AtZIK3       334 ETGVFLRHP---------------------LIDDPLYHDQFESSQICEIDLFAN------
BnaZIK2      293 NLKPFLNEN--------------------------------------EMDRLKL------
BnaZIK8      294 ------------------------------------------------------------
BnaZIK6      302 KPPQPEHLP----------------------MDVDHHHNENKSVSVSSSRKSNNGESYPW
AtZIK6       308 RPPQLEHLP----------------------MDVD--HNENKSVSSN--------EDYPW
AtZIK10      295 MPPQCEYRP----------------------MDVEYKKNTSVSICSSAK----SSQECAL
BnaZIK5      311 ERCLMSEGP----------------------PATRPSKPTSMDLDEDSNLPIVTFSDNSG
consensus    361         p                             n    ti    iel
```

```
                                          AD
BnaZIK4      364 --DQEEDKSSGNVDISIKGKRRDDG-GLFLRLRITDKE----------------------
AtZIK4       379 --DQEEEKKSGNVDITIKGKRRDDG-GLFLRLRIADKE----------------------
BnaZIK9      324 EEAEEYDNKFDNVHISIKGKRRDNGDGLFLRLRIADKE----------------------
AtZIK9       351 -DEEEEDKRFGSVDISIKGKRRDNGDGLFLRLKTVNKE----------------------
OsMAPKKK20   354 ---GHEDEPLGNVDITIKGRKSEDG-SIFLRLRIADND----------------------
BnaZIK3      329 ----DDEEDSDHVDISIKGKRNGSD-GIFLRLRISDAE----------------------
AtZIK3       367 ----DDE---DHVDISIKGKRNGDD-GIFLRLRISDAEGIVSIFFDSFKIIGLKNVTRFR
BnaZIK2      309 -----EDDELGRTQMTITGRLKAEDNTILLEVIIADEN----------------------
BnaZIK8      294 -----EEEEEENNDNGGTGRVVS-------------------------------------
BnaZIK6      340 CQTIGLQRFAEDKEFRLRGERSDDA-TASMLLRIGDSS----------------------
AtZIK6       336 SQTIELQRIAENKEFRLRGERSDDV-TASMVLRIADPS----------------------
AtZIK10      329 LQTMEVQRVAESTEFKLSGERRDDV-AASMALRIAGSS----------------------
BnaZIK5      349 SRCIEVRRAKRGNFFVLKGEENDEQ-SVSLILRIVDEN----------------------
consensus    421      eedk  d vdisikGkr dd  glflrlriad e

BnaZIK4      399 -----GRVRNIYFPFDIETDTALSVATEMVAELDMDDHGVTKIANMIDGEISRLVPSWRP
AtZIK4       414 -----GRVRNIYFPFDIETDTALSVATEMVAELDMDDHGVTKIANMIDGEISSLVPSWRP
BnaZIK9      362 -----GLVRNIYFPFDIESDTAISVAREMVEELEMDDRDVTKIANMIDGEIASLVPRWRS
AtZIK9       388 -----GCVRNIYFPFDIETDTAISVAREMVEELEMDDRDVTKIANMIDGEIASLVPNWS-
OsMAPKKK20   388 -----GHVRNIYFPFDIEADTALSVATEMVAELDITDHEVTRIAEMIDGEVSALVPDWRP
BnaZIK3      362 -----GRIRNIYFPFETETDTAWSVAAEMVSELDITNQDVAKIAEMIDAEIAALVPDWKV
AtZIK3       419 VLGNVGRIRNIYFPFETAIDTAWSVAVEMVSELDITNQDVAKIAEMIDAEIAALVPDWKN
BnaZIK2      342 -----GMAKKVAFPFGIMNDTSVDVAMEMVKELEITDWDPVEIAEMIDGEISSLVPGWRY
BnaZIK8          ------------------------------------------------------------
BnaZIK6      377 -----GKGRIVHFAFYLNSDTATAIAEEMVEELHLTSQEVIVIADMIDDLIMQLHSERSS
AtZIK6       373 -----GKCRIVHFAFYLESDTATAIAEEMVEELHLTSQEVVVIADMIDDFIMQLLSDR-T
AtZIK10      366 -----GQARKVDFDFNLKTDTARAVTGEMVEELDLSSHEVTVIAEMIDELIMKLKANR--
BnaZIK5      386 -----GRVRNIHFLFYQEGDTASKVSSEMVEQLELTDQNVTFIAELIDILLVNMIPTWKT
consensus    481      grvrniyfpfdietdta sva emv eldmtd dvtkiaemidgei  lvp wr

BnaZIK4      454 GPEFEESLAAAAAAAANANICSNCVSNRTSMGSVMDFLRTNPGANVAQCCRNGCGETHGR
AtZIK4       469 GPEFEECLAAAAAANAAS-ICNNCVSNRTSMGSVMDFLRTNPGANVIQCCRNGCGETHGR
BnaZIK9      417 GLGFESSF-----------------CNCASNRSAVDFN-------VRQCCRNMCGEKHGR
AtZIK9       442 --IFCSSE-----------------SNRSSVGSVMDFN-------EMQCGRDGCEEKHGR
OsMAPKKK20   443 GPGIEESQ--------DTTYCHNCGSNVSSCGSLYAYMS-------SAARGCQCAELHGR
BnaZIK3      417 NNN----------------------------------------VSNSNKEEEKSH
AtZIK3       479 DTESSQNV-----------------NNNKNNNTAGFCGECASNGYIQETVSSG---EKSH
BnaZIK2      397 EEGDETPH-----------------VYHSSSSSQASFSN--------YMTRGRQDWQQDD
BnaZIK8          ------------------------------------------------------------
BnaZIK6      432 SSHPNQTS---------------------------------------PHLAVREDHHEAA
AtZIK6       427 SSHHNQNS---------------------------------------PRLTH--EDHEAA
AtZIK10      419 -SLPNANS---------------------------------------VYQSK---DEEAG
BnaZIK5      441 DVTVDHLI---------------------------------------HSQLNQSSRSHQN
consensus    541                                   s                   e hgr

BnaZIK4      514 FEEITIRETEVRLREIWKLQQQQESRELSSIDSGQNHSEEEEEE--VYEDPEITFSCEAS
AtZIK4       528 FEEITIRETEVRLRELWKLQQQQESRELSSIDSGHNHSEEEEEEEEVLYEDPENMFSCEAG
BnaZIK9      453 FEEITSGVNNSDEEEVFSCRSSD-------------------------------------
AtZIK9       476 FEEITFEITVNDSDEED-------------------------------------------
OsMAPKKK20   488 FEEITFQANGEQTDLQDSGGSSDDGGGQTQHVKDQEAVHSNGFVQMGRRGPRDQFCFSSF
BnaZIK3      432 HYHHQFECSDDRSCSSVHGRFEEISYQAEGQEQGS--GDVVVVSGEG-NNNRIHYADIWG
AtZIK3       519 HNHHEFDSSEDKSCSSVHG-----------------------------------RFADMWG
BnaZIK2      432 LHDETHSQSSYSSGSLSNLNYTYVDENSSQPPAVNRTHNVTRFRPEESYHVRSGKANMHA
BnaZIK8          ------------------------------------------------------------
BnaZIK6      453 NQLTANSKDE----ESMKSGISTDYYLPFSSNGS-----AGQEAES-MSSFLDSCSMMST
AtZIK6       446 NQQTVNSKDEEAAGQSMKSDISADYYFPYSANDGNAAMEAGRDAES-MSSYLDSCSMMST
AtZIK10      436 --------------ESMKSEISADYYHRVSSNEGSRLGCCCEAVESLLSSFLDSCSMVSN
BnaZIK5      462 EAKPQSQEESTFHDACESVRHSWTSDCPRSEEEEKQFVDAIKGENGSDNQEAEEATEPVS
consensus    601                    k   s
```

```
BnaZIK4       572 N----TLNHLTGSGSFSFLPSLYCDEV-EKTEDQVQQEFRWLKAKCQMDIREMHDEQLKS
AtZIK4        588 N----EINHISGSGSFSFMPSKYCDEPSEKTENQVQQELRWLKAKCQIELRDIQDEQLKT
BnaZIK9           ------------------------------------------------------------
AtZIK9            ------------------------------------------------------------
OsMAPKKK20    548 QEQSCSPRHYEYDTSLQAKGFDMKHEVKMAKYKARKMAHLRRAIHPSLDFDNLNGERRMK
BnaZIK3       489 LRDSRSDGGEEEEES--SLKPRRKVEGEWWPENEIRRELRWLKARHKRVRDHQT------
AtZIK3        545 LRESYSDDGEK--------QSSRKVRSGRWSENEMRRELRWLKARHKIQLMKMRGQTICE
BnaZIK2       492 ASSSSNLRQASDNRTLPRNRSLADVQGQLLQRSLTEEARIRRLIRTVGDVEAVGFQPPYA
BnaZIK8           ------------------------------------------------------------
BnaZIK6       503 LY---ISDNEYPDDLKTELNMIESQYNQSVQDLLKLKEDAVENAKRKWIMKKQKGL----
AtZIK6        505 IYNLSISDNDYPEDLKTELNLIESQFNQSFQDLLKLKEDAIENAKRKWITKKQKAVNIS-
AtZIK10       482 KQ---------SEDLKTELNVIESQYNQSCQRLLRMKEEAIEKAKRKWMKLS--------
BnaZIK5       522 LE--------EEERLRQELEEIEAKYQEEMKEIAKKREEAIMETKRKLSQKKVEQAG---
consensus     661                   l            e     e     ak

BnaZIK4       627 MWSETGEE---GLKDGFSG---SVSGLGRGGD-EDKVF----------------------
AtZIK4        644 RWPESGEEVEISPKDGFLG---SVSGLGREEDTVKEMF----------------------
BnaZIK9           ------------------------------------------------------------
AtZIK9            ------------------------------------------------------------
OsMAPKKK20    608 SSLNKLQSFHIGKNHNFRIPTCERSPGARDAEEDPDIFNLAYHSRHPDPGAQRARHCEVD
BnaZIK3       541 -----ICEKPSSASP---------------------------------------------
AtZIK3        597 TPIEISLTPGTSVSL---------------------------------------------
BnaZIK2       552 VSRKPPSSRR--------------------------------------------------
BnaZIK8           ------------------------------------------------------------
BnaZIK6           ------------------------------------------------------------
AtZIK6            ------------------------------------------------------------
AtZIK10           ------------------------------------------------------------
BnaZIK5           ------------------------------------------------------------
consensus     721

BnaZIK4           658 ---------------GGRSVPKCLKRTTSLPVDAIES
AtZIK4            679 ---------------GERLVPKCLKRTTSLPVDAIDS
BnaZIK9               -------------------------------------
AtZIK9                -------------------------------------
OsMAPKKK20        668 AQSSPDLMFTARSYYTGAQLPTNLPRTKSVTLNAVDA
BnaZIK3           551 ---------------------PLLYRAISLPVDAVDI
AtZIK3            612 ---------------------PLLYRAISLPVDAVDM
BnaZIK2               -------------------------------------
BnaZIK8               -------------------------------------
BnaZIK6               -------------------------------------
AtZIK6                -------------------------------------
AtZIK10               -------------------------------------
BnaZIK5               -------------------------------------
consensus         781
```

B

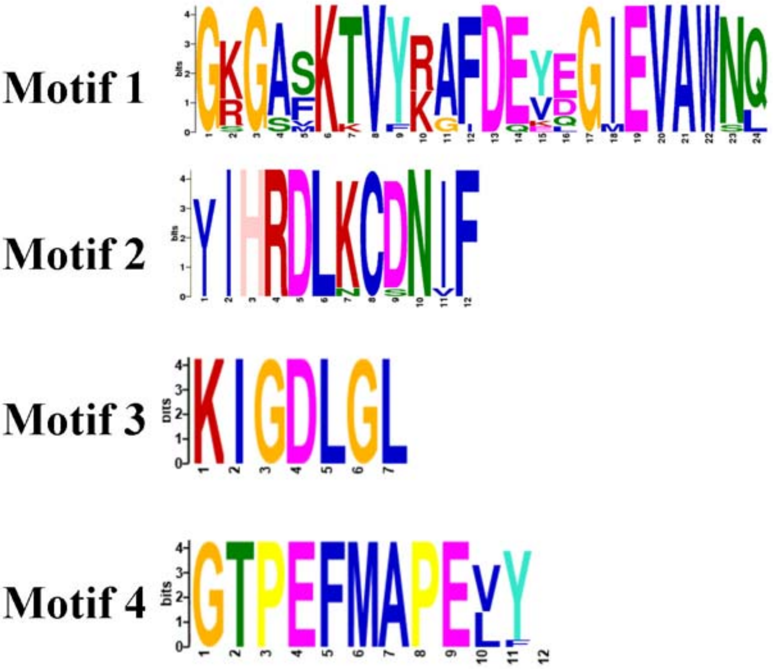

C

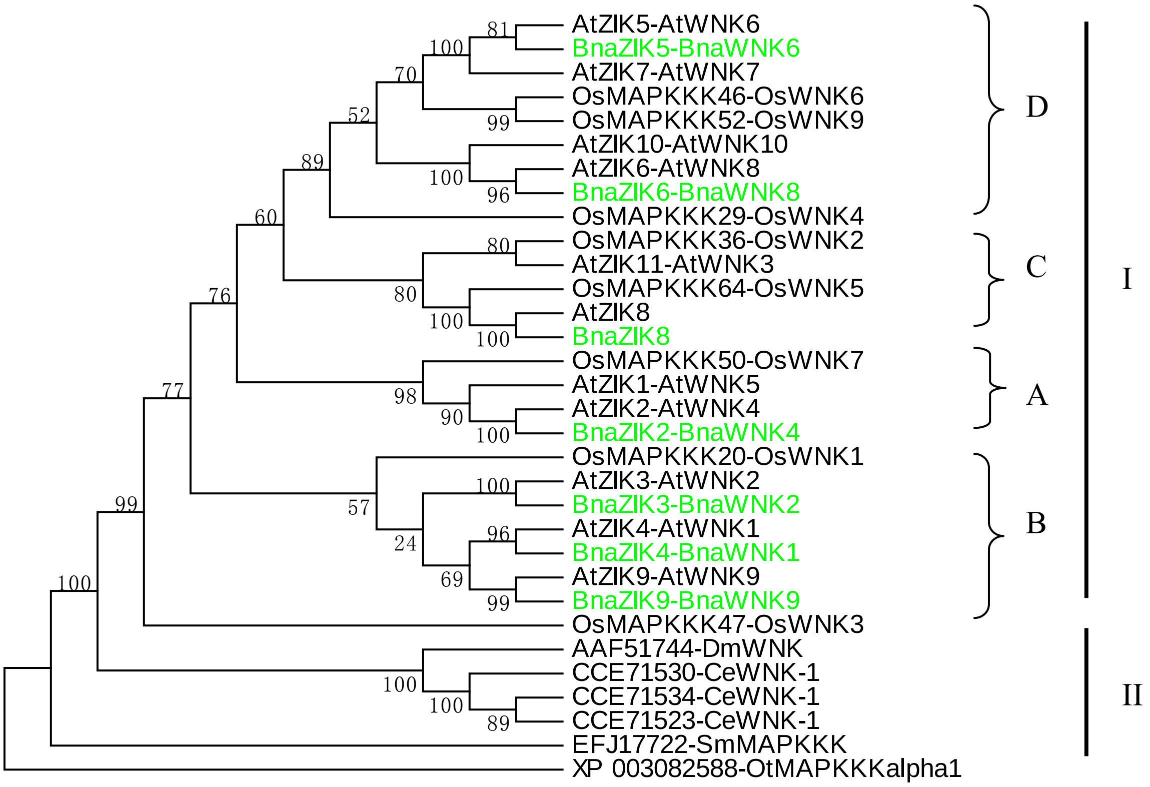

Figure S3. Multiple alignment of ZIK-subfamily MAPKKK proteins in representative species, schematic diagram of four motifs of BnaZIKs, and phylogenetic analysis of ZIKs (WNKs) in representative species. (A) Multiple alignment of ZIK-subfamily MAPKKK proteins in representative species. Multiple sequence alignment was performed using the ClustalX1.83 and illustrated by BOXSHADE (http://www.ch.embnet.org/software/BOX_form.html). Identical amino acids are shaded in black, and similar amino acids are shaded in gray. Four motifs were shown as

overbars, which are motif 1 of Serine/Threonine protein kinases active-site signature, motif 2 of Protein kinases ATP-binding region signature, motif 3 of Tyrosine kinase phosphorylation site and motif 4 of G(T/S)PX(F/Y/W)MAPEV. The Subdomain Iand II， as well as the autoinhibitory domain (AD) are indicated as above. The Lysine (K) residue in subdomain I and asparagine or serine in the serine/threonine protein kinase active-site signature was highlighted above. (B) Schematic diagram of four motifs of BnaZIKs. Motif analysis was determined by using MEME4.0 program as described in Methods. (C) Phylogenetic analysis of ZIKs (WNKs) in representative species. The phylogenetic tree was inferred using the maximum parsimony method of MEGA5.1 program. For clarity of presentation, the respective ZIK proteins are depicted by a two to three-letter code denoting the species in combination with numbers, locus ID or GenBank accession numbers representing the exact member from that species. A ZIK from the marine green alga *Ostreococcus tauri* (*Ot*) was used to root the tree. The ZIK proteins from animals form a distinct group (II) while those from plant species can be divided into four major subgroups (IA to ID), as supported by highly significant bootstrap values. The green highlighted proteins are from canola. The analysis involved 32 amino acid sequences. There are a total of 254 positions in the final dataset. The numbers on the nodes are percentages from a bootstrap analysis of 500 replicates. *A. thaliana* (At), *B. napus (Bna), O. sativa (Os), Selaginella moellendorffii (Sm）, Ostreococcus tauri (Ot), Drosophila melanogaster (Dm)* and *Caenorhabditis elegans (Ce)*.

A

```
BnaCTR1               1 ---------------------MEMPGARRSNYTLLSQFPDDQVSVSVTGAPPPHYDSSLS
AtCTR1                1 ---------------------MEMP-GRRSNYTLLSQFSDDQVSVSVTGAPPPHYDSLSS
OsMAPKKK6-DSM1        1 ----------------------------MKNFFRKLHIGEGSGDGASSSPPPPPSSRKGS
AtEDR1                1 ----------------------------------MKHIFK---KLHRGGNQEQQNRTNDA
OsMAPKKK1-EDR1        1 ----------------------------------MKNLFKSKIKWQHRSNDPASSQGQGQ
BnaRaf33              1 ------------------------------------------------------------
BnaRaf39              1 ------------------------------------------------------------
BnaRaf36              1 ------------------------------------------------------------
BnaRaf22              1 ------------------------------------------------------------
BnaRaf28              1 ------------------------------------------------------------
BnaRaf34              1 ------------------------------------------------------------
BnaRaf41              1 ------------------------------------------------------------
BnaRaf37              1 ------------------------------------------------------------
BnaRaf21              1 ------------------------------------------------------------
BnaRaf29              1 ------------------------------------------------------------
BnaRaf30              1 ------------------------------------------------------------
OsMAPKKK43-ILA1       1 ------------------------------------------------------------
BnaRaf17              1 ------------------------------------------------------------
BnaRaf23              1 ------------------------------------------------------------
BnaRaf46              1 ------------------------------------------------------------
BnaRaf27              1 ------------------------------------------------------------
BnaRaf35              1 MCSSNRVIRFAMDSGSVNSSSVTSPGSSLNDEPHRVKFLCSFLGSILPRPQDGKLRYVGG
consensus              1

BnaCTR1              40 -SASNNNSGNNGKSKS---GFDWDHHPSGGGGDHRP---PNRAG-NMYSSSLGLQRQSSG
AtCTR1               39 ENRSNHNSGNTGKAKAERGGFDWDPS-GGGGGDHRLNNQPNRVGNNMYASSLGLQRQSSG
OsMAPKKK6-DSM1       33 GGVGGNHHLHAEQRQPSASAVSSWLDSVPGRPQPPTPSTPSEAEGSPFSSSVGSGAEERR
AtEDR1               24 APPSDQNRIHVSANPPQ------ATPSSVTETLPVAGATSSMASPAP---TAASNRADYM
OsMAPKKK1-EDR1       27 GLPQRRRRRHRRRAPRRGGRLRYPCRRYRRRPLPPPRRRRGLAAAGPGGGGGGTGGEDYM
BnaRaf33              1 ------------------------------------------------------------
BnaRaf39              1 ------------------------------------------------------------
BnaRaf36              1 ------------------------------------------------------------
BnaRaf22              1 ------------------------------------------------------------
BnaRaf28              1 ------------------------------------------------------------
BnaRaf34              1 ------------------------------------------------------------
BnaRaf41              1 ------------------------------------------------------------
BnaRaf37              1 ------------------------------------------------------------
BnaRaf21              1 ------------------------------------------------------------
BnaRaf29              1 ------------------------------------------------------------
BnaRaf30              1 ------------------------------------------------------------
OsMAPKKK43-ILA1       1 ------------------------------------------------------------
BnaRaf17              1 ------------------------------------------------------------
BnaRaf23              1 ------------------------------------------------------------
BnaRaf46              1 ------------------------------------------------------------
BnaRaf27              1 ------------------------------------------------------------
BnaRaf35             61 ETRIVSVTRDVSYEELMSKMRELYEDAAVLKYQQPDEDLDALVSVVNDDDVVNMMEEYDK
consensus             61
```

```
BnaCTR1           92 SSFGESSLSGDYYVPTLSAAGNEIEMVGFPQDVGL--------GDSRMQMGMDSAGGSSS
AtCTR1            98 SSFGESSLSGDYYMPTLSAAANEIESVGFPQDDGFRLGFGGGGGDLRIQMAADSAGGSSS
OsMAPKKK6-DSM1    93 QSVAAERRRSQEEEWERRRSQEEEAVREMRRSQEE--------DEVEERVIRESSEAEER
AtEDR1            75 SSEEE--------------------------------------YQVQLALAISASNSQSS
OsMAPKKK1-EDR1    87 LSEEE--------------------------------------FQMQLAMALSASNSECV
BnaRaf33           1 ------------------------------------------------------------
BnaRaf39           1 ------------------------------------------------------------
BnaRaf36           1 ------------------------------------------------------------
BnaRaf22           1 ------------------------------------------------------------
BnaRaf28           1 ------------------------------------------------------------
BnaRaf34           1 ------------------------------------------------------------
BnaRaf41           1 ------------------------------------------------------------
BnaRaf37           1 ------------------------------------------------------------
BnaRaf21           1 ------------------------------------------------------------
BnaRaf29           1 ------------------------------------------------------------
BnaRaf30           1 ------------------------------------------------------------
OsMAPKKK43-ILA1    1 ------------------------------------------------------------
BnaRaf17           1 ------------------------------------------------------------
BnaRaf23           1 ------------------------------------------------------------
BnaRaf46           1 ------------------------------------------------------------
BnaRaf27           1 ------------------------------------------------------------
BnaRaf35         121 LGSGDGFTRLRIFLFSSPEMDGSSGYDDQRESERRYVDALNNLVEGTDFRKVQQYPDSPR
consensus         121

BnaCTR1          144 GKSWAQQTEESYQLQLALALRLSSEATCADDPNFLDPVPDESALRTSPSSAETVSHRFWV
AtCTR1           158 GKSWAQQTEESYQLQLALALRLSSEATCADDPNFLDPVPDESALRTSPSSAETVSHRFWV
OsMAPKKK6-DSM1   145 KRVREKEDDDLEEFQLQLVLEMSARDNPEEMEIEVAKQISLGFCPPQSSTAEALAARYWN
AtEDR1            97 EDPEKHQIRAATLLSLGSHQRMDSRRDSS------------------EVVAQRLSRQYWE
OsMAPKKK1-EDR1   109 GDLDGEQIRKAKLISLGRGNRFAAVRDD-------------------EQTADALSRRYRD
BnaRaf33           1 ------------------------------------------------------------
BnaRaf39           1 ------------------------------------------------------------
BnaRaf36           1 ------------------------------------------------------------
BnaRaf22           1 ------------------------------------------------------------
BnaRaf28           1 ------------------------------------------------------------
BnaRaf34           1 ------------------------------------------------------------
BnaRaf41           1 ------------------------------------------------------------
BnaRaf37           1 ------------------------------------------------------------
BnaRaf21           1 ------------------------------------------------------------
BnaRaf29           1 ------------------------------------------------------------
BnaRaf30           1 ------------------------------------------------------------
OsMAPKKK43-ILA1    1 ------------------------------------------------------------
BnaRaf17           1 ------------------------------------------------------------
BnaRaf23           1 ------------------------------------------------------------
BnaRaf46           1 ------------------------------------------------------------
BnaRaf27           1 ------------------------------------------------------------
BnaRaf35         181 FNLNNDDFSMVEPFNQLAIESGGGGSQRGNEIPVAQYNNLHQLRIPRVGSGQMIAARYGD
consensus         181
```

```
BnaCTR1          204 NG-----------------CLSYYDKVPDGFYMIDGLDPYIWTLCIDLNESGRIPSIESL
AtCTR1           218 NG-----------------CLSYYDKVPDGFYMMNGLDPYIWTLCIDLHESGRIPSIESL
OsMAPKKK6-DSM1   205 FN-----------------ALGYDDRISDGFYDLYVTG--------NGPASITMPSLKDL
AtEDR1           139 YG-----------------VLDYEEKVVDSFYDVYSLS-------TDSAKQGEMPSLEDL
OsMAPKKK1-EDR1   150 YN-----------------FLDYHEKVIDGFYDIFGPS-------MESSKQGKMPSLADL
BnaRaf33           1 ------------------------------------------------------------
BnaRaf39           1 ------------------------------------------------------------
BnaRaf36           1 ------------------------------------------------------------
BnaRaf22           1 ------------------------------------------------------------
BnaRaf28           1 ------------------------------------------------------------
BnaRaf34           1 ------------------------------------------------------------
BnaRaf41           1 ------------------------------------------------------------
BnaRaf37           1 ------------------------------------------------------------
BnaRaf21           1 ---------------------------------MTNTDESESCGSR---AVVASPSKENP
BnaRaf29           1 --------------------------------MAIKDETEESCGSR---AVVS-VTKENP
BnaRaf30           1 -----------------------------------MIVVMESCGSR---VLPMMSEAAME
OsMAPKKK43-ILA1    1 ----------------------------------------MDHGGQVSPVPATATAPRKV
BnaRaf17           1 ------------------------------------------------------------
BnaRaf23           1 ------------------------------------------------------------
BnaRaf46           1 ------------------------------------------------------------
BnaRaf27           1 ------------------------------------------------------------
BnaRaf35         241 VEGTWSPYYSPRHHGHFQEFPSSPSSARYRMPYGEVLDKGFDRMPEDYARAGHHPLYEHQ
consensus        241

BnaCTR1          247 RAIDSGVDSSLEAILVDRRVDPAFKELHN-------RVHDISCSCITTKEVVDQLAKLIC
AtCTR1           261 RAVDSGVDSSLEAIIVDRRSDPAFKELHN-------RVHDISCSCITTKEVVDQLAKLIC
OsMAPKKK6-DSM1   240 RAQSLSHRVNWEAVLVHRGEDPELMKLDQTALIMSLELRESKPSEFVGNDLVQKLAGLVA
AtEDR1           175 ESNHG--TPGFEAVVVNRPIDSSLHELLEIA-----ECIALGCSTTSVSVLVQRLAELVT
OsMAPKKK1-EDR1   186 QTGIG--DLGFEVIVINRAIDTTLQEMEQVA-----QCILLDFPVANIAALVQRIAELVT
BnaRaf33           1 ------------------------------------------------------------
BnaRaf39           1 ------------------------------------------------------------
BnaRaf36           1 ------------------------------------------------------------
BnaRaf22           1 ------------------------------------------------------------
BnaRaf28           1 ------------------------------------------------------------
BnaRaf34           1 ------------------------------------------------------------
BnaRaf41           1 ------------------------------------------------------------
BnaRaf37           1 ------------------------------------------------------------
BnaRaf21          25 RQYRMKLDVYGEILQRLQESNYEEAALPDFEDQLWQHFNRLPARYALDVKVERAEDVLTH
BnaRaf29          25 RQQRMKLEVYGDVLQRIQESNYEEANLPDFDDHLWLHFNRLPARYAMDVNVERAEDVLTH
BnaRaf30          23 RRERMKVEVFEEVILRLRQSDNIDTDLPGFVDDLWAHFNRLPARYALDVNIERAEDVVMH
OsMAPKKK43-ILA1   21 RREHMRLGVYHDVLQRLRDAGAPEALAPDFAEKLWTHFHRFNASYAMDVNVERAEDVLMH
BnaRaf17           1 ------------------------------------------------------------
BnaRaf23           1 ------------------------------------------------------------
BnaRaf46           1 ------------------------------------------------------------
BnaRaf27           1 ------------------------------------------------------------
BnaRaf35         301 GQVPDNVVWVPAGATPLESKGGFPGNVLHGGPSGYEGGGNICESCRVPFHKNHQPFDQAN
consensus        301
```

```
BnaCTR1          300 NRMGGSVIMGEDELVPMWKECINGLKECFKVVVPIGSLSVGLCRHRALLFKVLADIIDLP
AtCTR1           314 NRMGGPVIMGEDELVPMWKECIDGLKEIFKVVVPIGSLSVGLCRHRALLFKVLADIIDLP
OsMAPKKK6-DSM1   300 RHMGGTFFDSEGMLVKYQKMMRYLRTSIGSVVVPLGQLKIGLARHRALLFKVLADNIGIP
AtEDR1           228 EHMGGSAEDSSIVLARWTEKSSEFKAALNTCVFPIGFVKIGISRHRALLFKVLADSVRLP
OsMAPKKK1-EDR1   239 DHMGGPVKDANDMLTRWLEKSTELRTSLHTSLLPIGCIKIGLSRHRALLFKILADSVGIP
BnaRaf33           1 ------------------------------------------------------------
BnaRaf39           1 ------------------------------------------------------------
BnaRaf36           1 ----------------------------IDEEASSWIRRAKFSQTVSYRLNSSKLAFFPV
BnaRaf22           1 ------------------------------------------------------------
BnaRaf28           1 ------------------------------------------------------------
BnaRaf34           1 ------------------------------------------------------------
BnaRaf41           1 ------------------------------------------------------------
BnaRaf37           1 ------------------------------------------------------------
BnaRaf21          85 QRLLKLAEDPATR-PVFDVHSVQVTPRNS------ADSDPALEEDAQSSSHTSGQGVLAP
BnaRaf29          85 QRLLKLAEDPATR-PVFEVRCVQVSPSLNGHSADIDASDPAVKEDAQSSYHSSR--VLAP
BnaRaf30          83 HRLLLSALDPHNRRPAIQVRLVQVQQPPPDS-------DSLTNETSSPPRRKRS--IHPP
OsMAPKKK43-ILA1   81 MKLLEKAIHPENQ-PAFSVRIVQVPLDIDAS---EADSQSNITEDDNCPTPRTP-AEHPA
BnaRaf17           1 --------------------------------------------------------MSSE
BnaRaf23           1 -----------------MANIAGQLKRGISRQLSSVSLRRTFSRQFTRQASHDPRRINMR
BnaRaf46           1 -----------------MENVAAQLKRGISRQFSTGSMRRTLSRQFTRQSSLDPRRNNMR
BnaRaf27           1 ---------------------------------------------------MEDDYKTPR
BnaRaf35         361 GFQPVPSAHCAQCPPNRETFMLNADPKPPTPHGAYANETFGHERGWIGQQQVNPNPNPNP
consensus        361                                                         i
```

ACT domain

Ankyrin repeats

```
BnaCTR1          360 CRIAKGCKYCNRDDAASCLVRFGLDREYLVDLVGKPGHLWEPD-----------------
AtCTR1           374 CRIAKGCKYCNRDDAASCLVRFGLDREYLVDLVGKPGHLWEPD-----------------
OsMAPKKK6-DSM1   360 CRLLKGRQYTGSDDGALNIVKFDDGREFIVDLVADPGTLIPSDGAVLSTEFEESSFSNNH
AtEDR1           288 CRLVKGSHYTGNEDDAVNTIRLEDEREYLVDLMTDPGTLIPADFASASNNTVEP------
OsMAPKKK1-EDR1   299 CKLVKGSNYTGDDDDAINIIKMN-EREFLVDLMAAPGTLIPSDVLSWKGNSLNSNARLTQ
BnaRaf33           1 ------------------------------------------------------------
BnaRaf39           1 ------------------------------------------------------------
BnaRaf36          33 KLNQEKLPGLKPIPDTSSSSTSTQADVYVVDSQVQRNPVTNKQRSVSPSPQMALPD----
BnaRaf22           1 ----------MLDGAKFNVLNHNNNDNNYYTFTQDFYQKLN-EGTNMSMS-MQTSN----
BnaRaf28           1 ----------MLEGPKFDMHAVG-NHHNYDAFTQDFYQKLGEEGTNMSTDSIQTSN----
BnaRaf34           1 ------------------------------------------------------------
BnaRaf41           1 ------------------------------------------------------------
BnaRaf37           1 ------------------------------------------------------------
BnaRaf21         138 PTFGSSPNFEAITLG-NKIVEDV--DSAVNATLT-TRPMHEITFSTIDKPKLLSQL----
BnaRaf29         142 PTFGSSPNFEAFTQAYKHHAEDD--DSAVNAQFPNSRPMHEITFSTIDKPKLLSHL----
BnaRaf30         134 PAFGSSPNLEALALAAHD-HDHDEGDNSVRNNSLYSRPLHEITFSTLDKPKLLLQL----
OsMAPKKK43-ILA1  136 PIFGSTTALKALVRQASSKNLLD----DNQDIDAILRPMHEITFASDDKPKGLTQL----
BnaRaf17           5 ASATAGDGGEQAVSAAGTSAGYDKQKEKARVSRTSLILWHAHQNDAAAVRKLLEED----
BnaRaf23          44 FSFGRQSSLDPIRRS-PEGSG-HPQLAVPDNLDATMQLLFVECGGDVEGVRDLLDD----
BnaRaf46          44 FSFGRQSSLDPIRRS-PESLGCEPKMSVPENLDSTMQLLFMASKGDVDGVEELLDE----
BnaRaf27          10 FTIGRQSSMAPEKIPEPSIHSEEEVLEDGEEIDGGVRLMYLCNEGDVEGIKELLDS----
BnaRaf35         421 PRIDEGRPLLSIVGRPSDHYTLDGPGMNYPFGHRPGPEISNEGFHDKPLGGIPLNPSNPS
consensus        421                                       l              v
```

```
BnaCTR1          403 ------------------------------------------------------------
AtCTR1           417 ------------------------------------------------------------
OsMAPKKK6-DSM1   420 HFNKDNDIRQLGSSNSLSNSACSSFECELLDRRSTWINVGPSDSDGATTSQTSKNNQQNT
AtEDR1           342 ---------------------------------------------------CNSNGNKFP
OsMAPKKK1-EDR1   358 N----------------------------------------PLAGSSSTTDSNLSANALP
BnaRaf33           1 ------------------------------------------------------------
BnaRaf39           1 ------------------------------------------------------------
BnaRaf36          89 ------------------------------------------------------------
BnaRaf22          45 ------------------------------------------------------------
BnaRaf28          46 ------------------------------------------------------------
BnaRaf34           1 ------------------------------------------------------------
BnaRaf41           1 ------------------------------------------------------------
BnaRaf37           1 ------------------------------------------------------------
BnaRaf21         190 ------------------------------------------------------------
BnaRaf29         196 ------------------------------------------------------------
BnaRaf30         189 ------------------------------------------------------------
OsMAPKKK43-ILA1  188 ------------------------------------------------------------
BnaRaf17          61 ------------------------------------------------------------
BnaRaf23          98 ------------------------------------------------------------
BnaRaf46          99 ------------------------------------------------------------
BnaRaf27          66 ------------------------------------------------------------
BnaRaf35         481 AEERGFHYGNNLYAPGPESVHSASHSHIYPQQNIWQNVPNHISGAPGLPMQQVNGTANQT
consensus         481

BnaCTR1          403 --------------------------------SLLNGPSTISISSPLRFPRPRPVEP---
AtCTR1           417 --------------------------------SLLNGPSSISISSPLRFPRPKPVEP---
OsMAPKKK6-DSM1   480 LSDSFGILSVSTFTSENRPITNESRSTDDIAAAKNKERSSVTINSSSTSPSPSSPEVGST
AtEDR1           351 TAQFSNDVPKLSEG-------EGSSHSSMANYSSSLDRRTEAERTDSSYPKVGPLRNIDY
OsMAPKKK1-EDR1   378 PGHKGGQLPLFSSGDWILASQSGYEKDGATTSSQASSSGTTSVAAGSAFDSSWTLVSHGQ
BnaRaf33           1 ------------------------------------------------------------
BnaRaf39           1 ------------------------------------------------------------
BnaRaf36          89 ------------------------------------------------------------
BnaRaf22          45 ------------------------------------------------------------
BnaRaf28          46 ------------------------------------------------------------
BnaRaf34           1 ------------------------------------------------------------
BnaRaf41           1 ------------------------------------------------------------
BnaRaf37           1 ------------------------------------------------------------
BnaRaf21         190 ------------------------------------------------------------
BnaRaf29         196 ------------------------------------------------------------
BnaRaf30         189 ------------------------------------------------------------
OsMAPKKK43-ILA1  188 ------------------------------------------------------------
BnaRaf17          61 ------------------------------------------------------------
BnaRaf23          98 ------------------------------------------------------------
BnaRaf46          99 ------------------------------------------------------------
BnaRaf27          66 ------------------------------------------------------------
BnaRaf35         541 VIRNPMDSASRYSIGVENQSVLVGSPQNISGFDAMSSPGQPYYPNPHFQDRAFPLDPNWV
consensus         541
```

```
BnaCTR1          428 ------------------------------------------------------------
AtCTR1           442 ------------------------------------------------------------
OsMAPKKK6-DSM1   540 PAVRRMKVKDISEYMINAAKENPQLAQKIHEVLLENGVVAPPDLFSEDSMEEPKDLIVYD
AtEDR1           404 SSPSSVTSSTQLENNSSTAIGKGSRGAIIECSRTNMNIVPYNQNSEEDPKNLFADLNPFQ
OsMAPKKK1-EDR1   438 SDDPSTSAGMSAQQKVILPGGEHPWNENINARNENIKLVSDLQGNSES-INLFADLNPFG
BnaRaf33           1 ------------------------------------------------------------
BnaRaf39           1 ------------------------------------------------------------
BnaRaf36          89 ------------------------------------------------------------
BnaRaf22          45 ------------------------------------------------------------
BnaRaf28          46 ------------------------------------------------------------
BnaRaf34           1 ------------------------------------------------------------
BnaRaf41           1 ------------------------------------------------------------
BnaRaf37           1 ------------------------------------------------------------
BnaRaf21         190 ------------------------------------------------------------
BnaRaf29         196 ------------------------------------------------------------
BnaRaf30         189 ------------------------------------------------------------
OsMAPKKK43-ILA1  188 ------------------------------------------------------------
BnaRaf17          61 ------------------------------------------------------------
BnaRaf23          98 ------------------------------------------------------------
BnaRaf46          99 ------------------------------------------------------------
BnaRaf27          66 ------------------------------------------------------------
BnaRaf35         601 PSENQAVRSEYLQGLKPLTGPMLQTNLDGAPIMQTPDCVEVVRPVESKVAQGGEHINCVD
consensus        601
```

ACT domain **Ankyrin repeats**

```
BnaCTR1          428 ----------AVDFRELAKQYFTDSESLNLVFDPASDDIGFSMFHR-----GGENDGSAE
AtCTR1           442 ----------AVDFRLLAKQYFSDSQSLNLVFDPASDDMGFSMFHRQYDNPGGENDALAE
OsMAPKKK6-DSM1   600 TTLFQSKDEMKKRMNELGSREYADRGHGPLLPHHPGHELPSKVPHRAPLDSLKPVEGLGI
AtEDR1           464 NKGADKLYMPTKSGLNNVDDFHQQKNN--PLVGRSPAPMMWKNYS-CNEAPKRKENSYIE
OsMAPKKK1-EDR1   497 GREPKRTSVPLNGPDNRNNELQRRRENVVPSTRRPQQRLVMKNWSPYNDVSNNKQYNYVE
BnaRaf33           1 --------------------MKEGNDGFVRADQIDLKSLDEQLERHLSRALTLEKNKKRD
BnaRaf39           1 ---------------------------METPNEIKASPGNNLRNRGAVGNDSKKDMIFRA
BnaRaf36          89 --------------VFKEARSERQRFSTPHPRRMDSEKGMKPKLSHKNSFDKRRSFNLRS
BnaRaf22          45 --------------AGGFVSMSVDNSSVGSSDALIGHPGLKPLRNNAYSPSVGQSVYRPR
BnaRaf28          46 --------------AGGSVSMSVDNSSVGSSDALIGHPGLKPMR-HPYSLSVGQSVFRPG
BnaRaf34           1 ------------------------------------------------------------
BnaRaf41           1 ----------------------------------------------------------MG
BnaRaf37           1 -----------------------------MPRGYQRAPSIEATTEIPAEKKLHPNYPFLM
BnaRaf21         190 --------------TSLLGELGLNIQEAHAFSTVDGFSLDVFVVDGWSQEETDGLKDALS
BnaRaf29         196 --------------TALLGELGLNIQEAHAFSTADGFSLDVFVVDGWSQEETEGLKDALK
BnaRaf30         189 --------------TALLAELGLNIQEAHVFSTTDGFSLDVFVVDGWPYEEVDRLRVALE
OsMAPKKK43-ILA1  188 --------------SSLLGNLNLDIKEVHALSTNDGYFLDIFIVIGWDHKETQLLEEALE
BnaRaf17          61 -----------------PSLVHARDYDKRTPLHVASLHGWIDVVNCLIEFGADVNAQDRW
BnaRaf23          98 -----------------GIDVNSIDLDGRTALHIAACEGHVEVVKLLLTRKANIDARDRW
BnaRaf46          99 -----------------GIDVNSIDLDGRTALHIASCEGHYDVVKVLLSRRANIDARDRW
BnaRaf27          66 -----------------GIDANYRDIDDRTALHVAACQGLKDVVEILLDRGADVDPKDRW
BnaRaf35         661 TGVSDGVPCLDKHQPLAEGKNDIVEVSPSAAAPPEGAELSVERLSFLPELMESVKRAALE
consensus        661                     l              g  i   ml
```

```
BnaCTR1          473 NGGGSVPPGANMPPQNIMRASNQVQDAVPINAPPINQPVVNRANRDLG------------
AtCTR1           492 NGGGSLPPSANMPPQNMMRASNQIEAAP-MNAPPISQPVPNRANRELG------------
OsMAPKKK6-DSM1   660 DHPPDIQDNTSFISQYEPSAPPQEASSQLTKQLPVTAAAVATAAVVASSMVVAAAKSNND
AtEDR1           521 NLLPKLHRDPRYGNTQSSYATSSSNGAISS---------NVHGRDNVTFVSPVAVPSSFT
OsMAPKKK1-EDR1   557 DSFARRNIGDNAASSSQVARPSAKNTNLNVGVRTDTPYMAAHNYDNSMAGSSAMKMTSTA
BnaRaf33          41 EEESAAAIGASASSSPVAALNGG-----------FVGQRKQRLE----------------
BnaRaf39          34 DRIDLKNLDIQLEKHLSRVWSRN-----------IEKNPKPKEE----------------
BnaRaf36         135 PSVPIRDLSTLRIQERVKSMKDTGWSKIFDNAGRKVSADDAAEE----------------
BnaRaf22          91 KITHPLDNDALTHALMDNMYPTEG--------------LANYEE----------------
BnaRaf28          91 RVTHALNDDALAQALMDSSYPTQG--------------LANYEE----------------
BnaRaf34           1 MILAMDSLNG----------------------------YRLEPK----------------
BnaRaf41           3 SASGFYSNEG----------------------------FELDPK----------------
BnaRaf37          32 SSKGLESDDDSDDDYSDLDYDDQF-------------HFSINDD----------------
BnaRaf21         236 KEILKLKDQPGSKQKSIAFFEHDKSSNE-LIPACIEIPTDGTDE----------------
BnaRaf29         242 KEILKLKDQPSSRQKSITFFEHDKSTNE-LLPACVEIPTDGTDE----------------
BnaRaf30         235 TEAAKIELQDQSWPMQQSFSPE-KQNG--TAKDHVVIPTDGTDV----------------
OsMAPKKK43-ILA1  234 KEIHNYEPQMPSKSSCWPPELAGKQSLINSQVNHVQIPKDNTDE----------------
BnaRaf17         104 KNTPLADAEGARKQKMIELLKSHGGLSYGQNGSHFEPKPVQPPI----------------
BnaRaf23         141 GSTAAADAKYYGNMDVYNILKARGARVPKTKRTPMVVANPR-------------------
BnaRaf46         142 GSTAAVDAKYYGNTEVYNLLKARGAKAPKTRKTPMTVGNPK-------------------
BnaRaf27         109 GSTPLADAIFYKNVDVIKILETHGAKHP---MAPMHVKTPR-------------------
BnaRaf35         721 GAAEVKAHPEEANDPVRPELVENESEHVNAQVEHEDSDSDNPNNFKIEP-----------
consensus         721                                  i


BnaCTR1          521 ------------------------------------------------------------
AtCTR1           539 ------------------------------------------------------------
OsMAPKKK6-DSM1   720 VNFDVPVAAAATVTAAAVVATTAAVSKQYEHLEPGNQLHSLPSPSEGNESIEKSADEFWD
AtEDR1           572 STENQFRPSIVEDMNR-NTNNELDLQPHTAAVVHGQQND----ESHIHDHRKYTSDDIST
OsMAPKKK1-EDR1   617 GIGKVPDKVLYGDLDKGLTNSRLGDQPPIERHKWGNSVEGRIPTGTVHNQAKEHKENFDG
BnaRaf33          74 ------------------------------------------------------------
BnaRaf39          67 ------------------------------------------------------------
BnaRaf36         179 ------------------------------------------------------------
BnaRaf22         121 ------------------------------------------------------------
BnaRaf28         121 ------------------------------------------------------------
BnaRaf34          17 ------------------------------------------------------------
BnaRaf41          19 ------------------------------------------------------------
BnaRaf37          63 ------------------------------------------------------------
BnaRaf21         279 ------------------------------------------------------------
BnaRaf29         285 ------------------------------------------------------------
BnaRaf30         276 ------------------------------------------------------------
OsMAPKKK43-ILA1  278 ------------------------------------------------------------
BnaRaf17         148 ------------------------------------------------------------
BnaRaf23         182 ------------------------------------------------------------
BnaRaf46         183 ------------------------------------------------------------
BnaRaf27         147 ------------------------------------------------------------
BnaRaf35         770 ------------------------------------------------------------
consensus         781
```

```
BnaCTR1           521 ---------------------------------------------------LDGDDMDIPWCDLN
AtCTR1            539 ---------------------------------------------------LDGDDMDIPWCDLN
OsMAPKKK6-DSM1    780 KQNFEIDHGQDNTLDQEKDSAEVRQDAERTSDKSSGTESAKSEITLDDVAEFEIQWEEIT
AtEDR1            627 GCDPRLKDHESTSSSLDSTSYRNDPQVLDD-----------------ADVGECEIPWNDLV
OsMAPKKK1-EDR1    677 KQDNKKLHPDPKKSPLDRFMDTSMPSRNPESVSPSFARSHKLDTMFDDVSECEIHWEDLV
BnaRaf33           74 ----------------------------------------------------------WEIDPSKLI
BnaRaf39           67 ----------------------------------------------------------WEIELAKLE
BnaRaf36          179 ----------------------------------------------------------FRIDTSKLF
BnaRaf22          121 ----------------------------------------------------------WTINLRNLR
BnaRaf28          121 ----------------------------------------------------------WTIDLRKLH
BnaRaf34           17 ----------------------------------------------------------WEIDPQLLF
BnaRaf41           19 ----------------------------------------------------------WLVDPRHLF
BnaRaf37           63 ----------------------------------------------------------VLVDAKEVS
BnaRaf21          279 ----------------------------------------------------------WEIDVKQLK
BnaRaf29          285 ----------------------------------------------------------WEIDMKQLK
BnaRaf30          276 ----------------------------------------------------------WEINLKQLK
OsMAPKKK43-ILA1   278 ----------------------------------------------------------WEINFDVLD
BnaRaf17          148 ---------------------------------------------------PKKCDWEIEPAELD
BnaRaf23          182 -----------------------------------------------------EVPEYELNPQELQ
BnaRaf46          183 -----------------------------------------------------EVPEYELNPLELQ
BnaRaf27          147 -----------------------------------------------------EVPEYEINPSELD
BnaRaf35          770 ------------------------------------------------TKAEAEALSRGLQTIKNDD
consensus         841                                                        wei    l
```

Motif 1 (subdomain I)

Protein kinases ATP-binding region signature

(I/V)GXG(S/F)(F/Y) (S/G/A) (K/D/T/E)VX(K/R/H)(G/A) X(W/F)(H/F/N)G

```
BnaCTR1           535 IK--EKIGAGSFGTVHRAEWHGSDVAVKILMEQDFH--------AERVNEFLREVAIMKR
AtCTR1            553 IK--EKIGAGSFGTVHRAEWHGSDVAVKILMEQDFH--------AERVNEFLREVAIMKR
OsMAPKKK6-DSM1    840 IG--ERIGLGSFGEVYRGEWHGTEVAVKKFLQQDIS--------SDALEEFRTEVRIIKR
AtEDR1            671 IA--ERIGLGSYGEVYHADWHGTEVAVKKFLDQDFS--------GAALAEFRSEVRIMRR
OsMAPKKK1-EDR1    737 IG--ERIGLGSYGEVYRADWNGTEVAVKKFLDQDFY--------GDALDEFRSEVRIMRR
BnaRaf33           83 IK--TVLARGTFGTVHRGIYDGQD-VAVKLLDWGEEGHRSEAEIVSLRADFAQEVAVWHK
BnaRaf39           76 MS--NVIARGAYGIVYKGIYDGQD-VAVKVLDWGEDGYATTAETSALRASFRQEVGVWHK
BnaRaf36          188 LG--LRFAHGLYSKLYHGKYEDKA-VAVKLITVPDD-DENGCLGARLEKQFTKEVTLLSR
BnaRaf22          130 MG--PPFAQGAFGKLYKGSYNGVD-VAIKILERPEN---SLEKAQFMEQQFQQEVTMLAN
BnaRaf28          130 MG--PAFAQGAFGKLYRGTYNGED-VAIKLLERPEN---SPEKAQALEQQFQQEVSMLSF
BnaRaf34           26 VG--PKIGEGAHAKVYEGKYKNQT-VAIKIIHRGET----PEEIAKRDSRFLREVEMLSR
BnaRaf41           28 VG--PKIGEGAHAKVYEGKYRNQT-VAIKIIKRGES----PEEIAKRDSRFAREIAMLSK
BnaRaf37           72 LG--ELIGEGSSSKVYRGLFREVNPVSVRIFQPERA----AAVSVEQKRRFEREVLLLSK
BnaRaf21          288 IE--KKVASGSYGDLHKGTYCSQE-VAIKFLK-------PERVNTEMLREFSQEVYIMRK
BnaRaf29          294 IE--KKVACGSYGELYKGTYCSQE-VAIKILK-------PERVNTEMLREFSQEVYIMRK
BnaRaf30          285 FG--HKIASGSYGNLYKGTYLSQE-VAIKILK-------PERLDSELEKEFSQEVFIMRK
OsMAPKKK43-ILA1   287 IQ--EKVASGTYGDLYRGTYFGED-VAIKVLK-------SDRLNENMQEEFNEEVFIMRK
BnaRaf17          162 FSNAAMIGKGSFGEIVKAYWRGTPVAVKRILPSLSD-------DRLVIQDFRHEVDLLVK
BnaRaf23          195 VRKADGISKSCQGIYQVAKWNGTKVSVMILDKDLYK-------DNETIEAFKHELTLLEK
BnaRaf46          196 VRKVDGISK---GTYQVAKWNETRVSVKIFDKDSYS-------DPERVNAFNHELTLLAK
BnaRaf27          160 FTQSKEITK---GTYCMAMWRGIQVAVKKLDDKVLS-------DEDQVRKFHDELALLQR
BnaRaf35          789 LEEIRELGSGTYGSVYHGKWKGSDVAIKRIKASCFAG--KPSERERLIEDFWKEALLLSS
consensus         901 i    kig gsfg vyrg y g d vavkll              elv eF  Ev ilkk
```

```
BnaCTR1           585 LRHPNIVLFMGAVTQPP------------------NLSIVTEYLSRGSLYRLLHKSGAREQ
AtCTR1            603 LRHPNIVLFMGAVTQPP------------------NLSIVTEYLSRGSLYRLLHKSGAREQ
OsMAPKKK6-DSM1    890 LRHPNVVLFMGAITRVP------------------NLSIVTEFLPRGSLFRLIHRP--NNQ
AtEDR1            721 LRHPNVVFFLGAVTRPP------------------NLSIVTEFLPRGSLYRILHRP--KSH
OsMAPKKK1-EDR1    787 LRHPNIVLFMGAVTRPP------------------NLSIVSEYLPRGSLYKILHRP--NCQ
BnaRaf33          140 LDHPNVTKFIGATMGASGLQLQT-ESGPLAMPNNICCVVVEYLQGGALKSFLIKN-RRRK
BnaRaf39          133 LNHPNVTKFVGASMGTTNLKIPSSAEMENSLPQRACCVVVEYLPGGTLKQYLFRN-RRRK
BnaRaf36          244 LSHPNVIKFVGAYKDPP------------------SYCVLTEYLPEGSLRSYLHKP-ENRT
BnaRaf22          184 LKHPNIVRFIGACRKPM------------------VWCIVTEYAKGGSVRQFLTKR-QNRA
BnaRaf28          184 LKHPNIVRFIGACIKPM------------------VWCIVTEYTKGGSVRQFLTKR-QNRA
BnaRaf34           79 VQHKNLVKFIGACKEPV-------------------MVIVTELLQGGTLRKYLVNL-RPAC
BnaRaf41           81 VQHKNLVKFIGACKEPM-------------------MVIVTELLLGGTLRKYLVSL-RPKR
BnaRaf37          126 IQHENIVQFIGACVKPK-------------------LMIVTELMEGNTLHKFMLST-RPNP
BnaRaf21          338 VRHKNVVQFLGACTRSP------------------TLCIVTEFMARGSIYDFLHK--QKCA
BnaRaf29          344 VRHKNVVQFIGACTRSP------------------NLCIVTEFMARGSIYDFLHK--QKGV
BnaRaf30          335 VRHKNVVQFIGACTKPP------------------HLCIVTEFMPGGSVYDYLHK--QKGV
OsMAPKKK43-ILA1   337 IRHKNIVRFLGACTKSP------------------TLCIVTEFMKNGSVYDYLHK--RKGS
BnaRaf17          215 LRHPNIVQFLGAVTERK------------------SLMLITEYLRGGDLHQYLKEK---GG
BnaRaf23          248 VRHPNVVQFVGAVTQNV------------------PMMIVSEYHPKGDLGSYLQKK---GR
BnaRaf46          246 ARHPNIVQFVGAVTQNL------------------PMMIVVEHNPKGDLSGYLQKK---GR
BnaRaf27          210 LRHPNIVQVLGAVTQSN------------------PMMIVTEYLPRGDLRELLKRK---VQ
BnaRaf35          847 LHHPNVVSFYGIVRDGPD----------------GSLATVAEFMVNGSLKQFLQKK--DRT
consensus         961 lrHpNiv fmGa t pp                   l ivtEyl  gslk flhk
```

Motif 2

Serine/Threonine protein kinases active-site signature

[LIVMFYC]X[HY]XD[LIVMFY]KXXN[LIVMFYCT]XXX

```
BnaCTR1           628 LDERRRLSMAYDVAKGMNYLHNRN----------------------------PPIVHRDLK
AtCTR1            646 LDERRRLSMAYDVAKGMNYLHNRN----------------------------PPIVHRDLK
OsMAPKKK6-DSM1    931 LDERKRLRMALDVARGMNYLHNCT----------------------------PVIVHRDLK
AtEDR1            762 IDERRRIKMALDVAMGMNCLHTST----------------------------PTIVHRDLK
OsMAPKKK1-EDR1    828 IDEKRRIKMALDVAKGMNCLHISV----------------------------PTIVHRDLK
BnaRaf33          198 LAFKVVVQLALDLARGLSYLHSQK------------------------------IVHRDVK
BnaRaf39          192 LAIRVVVQLALDLSRGLSYLHSER------------------------------IVHRDVK
BnaRaf36          286 LSLKKLIEFALDIAKGMEYIHSRR------------------------------IIHRDLK
BnaRaf22          226 VPLKIAVKQALDVARGMAYVHAHN------------------------------LIHRDLK
BnaRaf28          226 VPLKLAVKQALDVARGMAYVHERN------------------------------FIHRDLK
BnaRaf34          120 LETPVAIGFALDIARGMECLHSHG------------------------------IIHRDLK
BnaRaf41          122 LDIRLAVAFALDIARAMECLHSHG------------------------------IIHRDLK
BnaRaf37          167 LDLKLAISFALDIARGMEFLNANG------------------------------IIHRDLK
BnaRaf21          379 FKLQTLLKVALDVAKGMCYLHQNNIIHRDLKTANLLMDEHGLAKGVCYLHQNNIIHRDLK
BnaRaf29          385 FKLQSLLKVALDVSKGMNYLHQN-----------------------------NIIHRDLK
BnaRaf30          376 FKLPALFKVAIDICKGMNYLHQN-----------------------------NIIHRDLK
OsMAPKKK43-ILA1   378 FKLPSLLKAAVDISKGMNYLHQN-----------------------------KIIHRDLK
BnaRaf17          255 LTPATAVNFALDIARGMTYLHNE----------------------------PNVIIHRDLK
BnaRaf23          288 LSPSKVLRFALDIARGMNYLHECK---------------------------PEPVIHCDLK
BnaRaf46          286 LSPSKALRFALDIARGMNYLHECK---------------------------PDPFIHCDLR
BnaRaf27          250 LKPATAVRYALDIARGMSYLHEIK---------------------------GDPIIHRDLE
BnaRaf35          890 IDRRKRLIIAMDTAFGMEYLHGKN------------------------------IVHFDLK
consensus        1021 l  r  lrmAlDiargm ylh                                 iiHrDlk
```

Motif 2 **Motif 3** **Motif 4 (Subdomain VIII)**

Tyrosine kinase phosphorylation site **GTXX(W/Y)MAPE**

[R/K]X{2,3}[D/E]X{2,3}Y

```
BnaCTR1           661 SPNLLVDK----KYTVKVCDFGLSRLKASTFLS------SKTAAGTPEWMAPEVLRD---
AtCTR1            679 SPNLLVDK----KYTVKVCDFGLSRLKASTFLS------SKSAAGTPEWMAPEVLRD---
OsMAPKKK6-DSM1    964 SPNLLVDK----NWVVKVCDFGLSKMKNKTFLS------SRSTAGTAEWMAPEVLRN---
AtEDR1            795 TPNLLVDN----NWNVKVGDFGLSRLKHNTFLS------SKSTAGTPEWMAPEVLRN---
OsMAPKKK1-EDR1    861 SPNLLVDN----NWNVKVCDFGLSRLKHSTFLS------SKSTAGTPEWMAPEVLRN---
BnaRaf33          229 TENMLLDK----TRTVKIADFGVARVEASNPND------MTGETGTLGYMAPEVLN----
BnaRaf39          223 TENMLLDY----QRNLKIADFGVARVEAQNPKD------MTGETGTLGYMAPEVLD----
BnaRaf36          317 PENVLIDK----DFQLKIADFGIA-CEEEYCDM------LADDPGTYRWMAPEMIK----
BnaRaf22          257 SDNLLISA----DKSIKIADFGVARIEVHTEG-------MTPETGTYRWMAPEMIQ----
BnaRaf28          257 SDNLLISA----DRSIKIADFGVARIEVQTEG-------MTPETGTYRWMAPEMIQ----
BnaRaf34          151 PENLLLTA----DHKTVKLADFGLAREESLTEM------MTAETGTYRWMAPELYSTVTL
BnaRaf41          153 PENLILSA----DHKTVKLADFGLAREESLTEM------MTAETGTYRWMAPELYSTVTL
BnaRaf37          198 PS--INDL----FWLNVQISLIQDCGIEETKGF------MTSEAGTYRWMAPEIFSHEAL
BnaRaf21          439 TANLLMDE----HGLVKVADFGVARVQIESGV-------MTAETGTYRWMAPEVIEH---
BnaRaf29          416 TANLLMDE----HDVVKVADFGVARVQTQSGV-------MTAETGTYRWMAPEVIEH---
BnaRaf30          407 AANLLMDE----NEVVKVADFGVARVKAQTGV-------MTAETGTYRWMAPEVIEH---
OsMAPKKK43-ILA1   409 TANLLMDE----HELIKVADFGVARVKAESGI-------MTAETGTYRWMAPEVIEH---
BnaRaf17          288 PRNVLVVNS--SADHLKVGDFGLSKLIKVQNS--HDVYKMTGETGSYRYMAPEVFKH---
BnaRaf23          322 PKNIMLD----NGGLLKVAGFGLISFEKLSSDKSKVLNHGAHIDLSNYCVAPEVYRD---
BnaRaf46          320 PKNILLD----RGGQLKICGFGLVKLSKISEDNFKVVKHEAHIDKSNYYVAPEIYKN---
BnaRaf27          284 PSNIILRD----DTGHLKVADFGVSKLVTVKED-----KPFTCLDTSCRYIAPEVFTS---
BnaRaf35          921 CENLLVNMRDPQRPVCKIGDLGLSKVKQKTLVS-------GGVRGTLPWMAPELLSG---
consensus        1081 s nllvd         vkvadfgl rl   t        mtgetgtyrwmAPEvl

BnaCTR1           708 -----EQSNEKSDVYSFGVILWELATLQQPWGNLNP---AQVVAAVGFKNKRLEIPRNLN
AtCTR1            726 -----EPSNEKSDVYSFGVILWELATLQQPWGNLNP---AQVVAAVGFKCKRLEIPRNLN
OsMAPKKK6-DSM1   1011 -----EPSDEKCDVFSYGVILWELCTLLQPWGGMNA---MQVVGAVGFQNRRLDIPDNTD
AtEDR1            842 -----EPSNEKCDVYSFGVILWELATLRLPWRGMNP---MQVVGAVGFQNRRLEIPKELD
OsMAPKKK1-EDR1    908 -----EQSNEKCDVYSFGVILWELATLRMPWSGMNP---MQVVGAVGFQDKRLDIPKEID
BnaRaf33          275 ----GNPYNRKCDVYSFGICLWERYCCDMPYPDLTF---SEVTSAVVRQNLRPDIPRCCP
BnaRaf39          269 ----GKPYNRRCDVYSFGICLWEIYCCAMPYPDLSF---ADVSSAVVRQNLRPDIPRCCP
BnaRaf36          362 ----RKPHGRKADVYSFGLVLWEMVAGAIPYEDMNP---IQAAFAVVHKNIRPAIPEDCP
BnaRaf22          302 ----HRTYNQKVDVYSFGIVLWELITGQLPFQNMTA---VQAAFAVVNRGVRPTIPNDCL
BnaRaf28          302 ----HRPYTQKVDVYSFGIVLWELITGLLPFQNMTA---VQAAFAVVNRGVRPTVPADCL
BnaRaf34          201 RLGEKKHYNNKVDAYSFAIVLWELLHNKLPFEGMSN---LQAAYAAAFKNVRPSA-ESLP
BnaRaf41          203 RHGEKKHYNHKVDAYSFAIVLWELINKLPFEGMSN---LQAAYAAAFKNLRPSA-EDLP
BnaRaf37          246 QVGEKKEYDHKVDVYSFTIVFWELLTNKVPFKGKNN---IFVAYAAS-KNQRPSL-DNIP
BnaRaf21          485 -----KPYSHKADVFSYAIVLWELLTGDIPYAFLTP---LQAAVGVVQKGLRPKIPKKTH
BnaRaf29          462 -----KPYDHRADVFSYAIVLWELLTGELPYSYLTP---LQAAVGVVQKGLRPKIPKQTH
BnaRaf30          453 -----KPYDHKADVFSYGIVLWELLTGKLPYEYMTP---LQAAVGVVQKGLRPKIPKKTH
OsMAPKKK43-ILA1   455 -----KPYDSKADVFSFGVVLWELLTGKIPHEFLTP---LQAAIGVVQEGLRPVIPKATD
BnaRaf17          341 -----RKYDKKVDVFSFAMILYEMLEGEPPFANYEPYEAAKHVSDG-HRPSFRS--KGCT
BnaRaf23          375 -----EIFDRSADSYSFGVVLYEMIEGVQPFHPKPPEEAVKLMCLEGRRPSFKAKTKSCP
BnaRaf46          373 -----NIFDKRVDVHSFGVILYEMTEGVSIFHPKPPEEIAELICMEGRRPTIKTKSKSYP
BnaRaf27          332 -----EEYDTKADVFSFALIVQEMIEGRMPFAEKEDSEASEAYASK-ERPLFKAPSKLYP
BnaRaf35          971 ---KSNMVSEKIDVYSFGIVMWELLTGEEPYADMHC---ASIIGGIVNNTLRPKIPQWCD
consensus        1141      kpy rk DvySfgivlwElvtg lpw  l p   mq    av  k lrp ipk
```

```
BnaCTR1           760 PQVAAIIETCWTNEPWKRPSFATIMDLLRPLIKSAVPPPNRLDM----------------
AtCTR1            778 PQVAAIIEGCWTNEPWKRPSFATIMDLLRPLIKSAVPPPNRSDL----------------
OsMAPKKK6-DSM1   1063 PAIAEIIAKCWQTDPKLRPSFADIMASLKPLLKNMTAQAPRQRVQQTDE-----------
AtEDR1            894 PVVGRIILECWQTDPNLRPSFAQLTEVLKPLNRLVLPTPQ--------------------
OsMAPKKK1-EDR1    960 PLVARIIWECWQKDPNLRPSFAQLTSALKTVQRLVTPSHQESQSPPVPQEIWVNSSTP--
BnaRaf33          328 SALAAVMKRCWDANPDKRPEMDEVVPMLESIDTTKGG-GMIPADQQQGCLCFRRETWSLS
BnaRaf39          322 TSLSNIMKRCWDANPVKRPEMEEVVKMLEGVDTSKGG-GMIPEDQRPGCFCFVSGRGP--
BnaRaf36          415 GAMKALIEQCWSVAPDKRPEFWQIVKVLEQFRVSLEREGSLNLTSNKICRDPRKSLKHWI
BnaRaf22          355 PALTEIMTRCWDGNAQVRPSFVEVVNMLEAAETEIMT--TARKARFRCCMSQPMTID---
BnaRaf28          355 PVLGEIMTRCWDANPEVRPCFAEVVNLLEAAETEIMT--TARKARFRCCMTQPMTID---
BnaRaf34          257 EDLGVIVTSCWNEDPNARPNFTQIIQLLLNYLSKVGSPALSAIPQR-ILASKNSLLPPDS
BnaRaf41          259 GELGLIVTSCWKEDPNERPNFTEIIQMLLRYLSTVSPPQIVPPPVRRVFSSENVVFSPDS
BnaRaf37          301 AGVGCLLEACWAADPKARPEFKEITASLEKLLRSLCSE--EDASSANVATEDSTTYLVQE
BnaRaf21          537 PRVKGLLERCWQQEPKERPDFEEIIEMLQQIMTEVNVVP---------------------
BnaRaf29          514 PKLTELLEKCWQQDPAQRPDFAEIKEMVTQLLHEVGDEEHQKDKRS-----GYFSGLRKG
BnaRaf30          505 PKMRELMERLWEKDPSLRPDFAEIKEQLEEIAKEVGEEGEEKKKASRGGGG-IFAALRRS
OsMAPKKK43-ILA1   507 PKLALLLESCWQQNAVNRPDFVQILQKLDEIAGEHGIDLTHPHKEKEKGGFFTFGKVH--
BnaRaf17          393 PDLRELIVKCWDADMNQRPSFLDILKRLEKIKETLPSDHHWGLFTS--------------
BnaRaf23          430 EEMRELIEECWDVKAVVRPTFSEIIVRLDRIFVQCSKQGWWKDTFKFPWK----------
BnaRaf46          428 PELKELIEECWHPDISVGPTFSEIICRLEIIVLNCSKQGWWKDTFKFPWK----------
BnaRaf27          386 HGLKTLIEECWQDKPAKRPTFREIIKRLESILHHMGHKRQWRMRPLTCFQNFEHKKKHNW
BnaRaf35         1025 PEWKGLMESCWASEPTERPSFADISQKLRNMAAAMNLK----------------------
consensus        1201 p l  lie cW  dp  rPsf eim lle l   m

BnaCTR1                ------------------------------------------------------------
AtCTR1                 ------------------------------------------------------------
OsMAPKKK6-DSM1         ------------------------------------------------------------
AtEDR1                 ------------------------------------------------------------
OsMAPKKK1-EDR1         ------------------------------------------------------------
BnaRaf33           387 AMLSKIIQNSRFVSCLLSSFLLLLIRKEKGLLGLFMVYDMFSIFFLNFPCFCSVW
BnaRaf39               ------------------------------------------------------------
BnaRaf36           475 KKLGPQGGGTTGGGVGGSSSSGGLGRSAMPNPKFA-------------------------
BnaRaf22               ------------------------------------------------------------
BnaRaf28               ------------------------------------------------------------
BnaRaf34           316 PGTSSLMATKLDECG----ETPKAKTEDKRKGLFFCFNQCY-------------------
BnaRaf41           319 PGTCSLMNVRDKDGSGQKVNAADSSEKETKGSFFFCCS----------------------
BnaRaf37           359 RVVYDFPKPKIKTSKRKKRNKVMNMMAPFLKIFRDCISK---------------------
BnaRaf21               ------------------------------------------------------------
BnaRaf29           569 HH----------------------------------------------------------
BnaRaf30           564 ATHH--------------------------------------------------------
OsMAPKKK43-ILA1        ------------------------------------------------------------
BnaRaf17               ------------------------------------------------------------
BnaRaf23               ------------------------------------------------------------
BnaRaf46               ------------------------------------------------------------
BnaRaf27           446 DMSSHDGSSSGSHL----------------------------------------------
BnaRaf35               ------------------------------------------------------------
consensus         1261
```

B

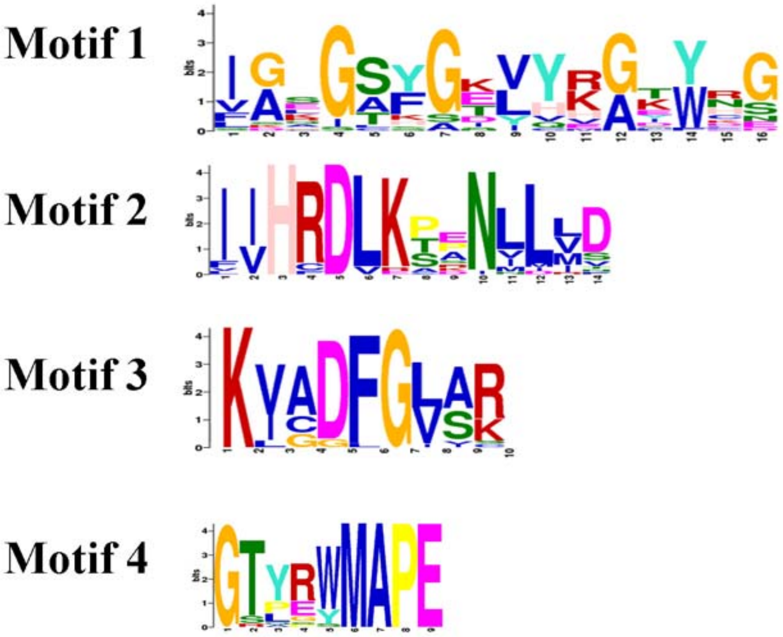

Figure S4. Multiple alignment of Raf-subfamily MAPKKK proteins in representative species and schematic diagram of four motifs of BnaRafs. (A) Multiple alignment of Raf-subfamily MAPKKK proteins in representative species. Multiple sequence alignment was performed using the ClustalX1.83 and illustrated by BOXSHADE (http://www.ch.embnet.org/software/BOX_form.html). Identical amino acids are shaded in black, and similar amino acids are shaded in gray. Four motifs were shown as overbars, which are motif 1 of Serine/Threonine protein kinases active-site signature, motif 2 of Protein kinases ATP-binding region signature, motif 3 of tyrosine kinase phosphorylation site and motif 4 of G(T/S)PX(F/Y/W)MAPEV. (B) Schematic diagram of four motifs of BnaRafs. Motif analysis was determined by using MEME4.0 program as described in Materials and Methods section.

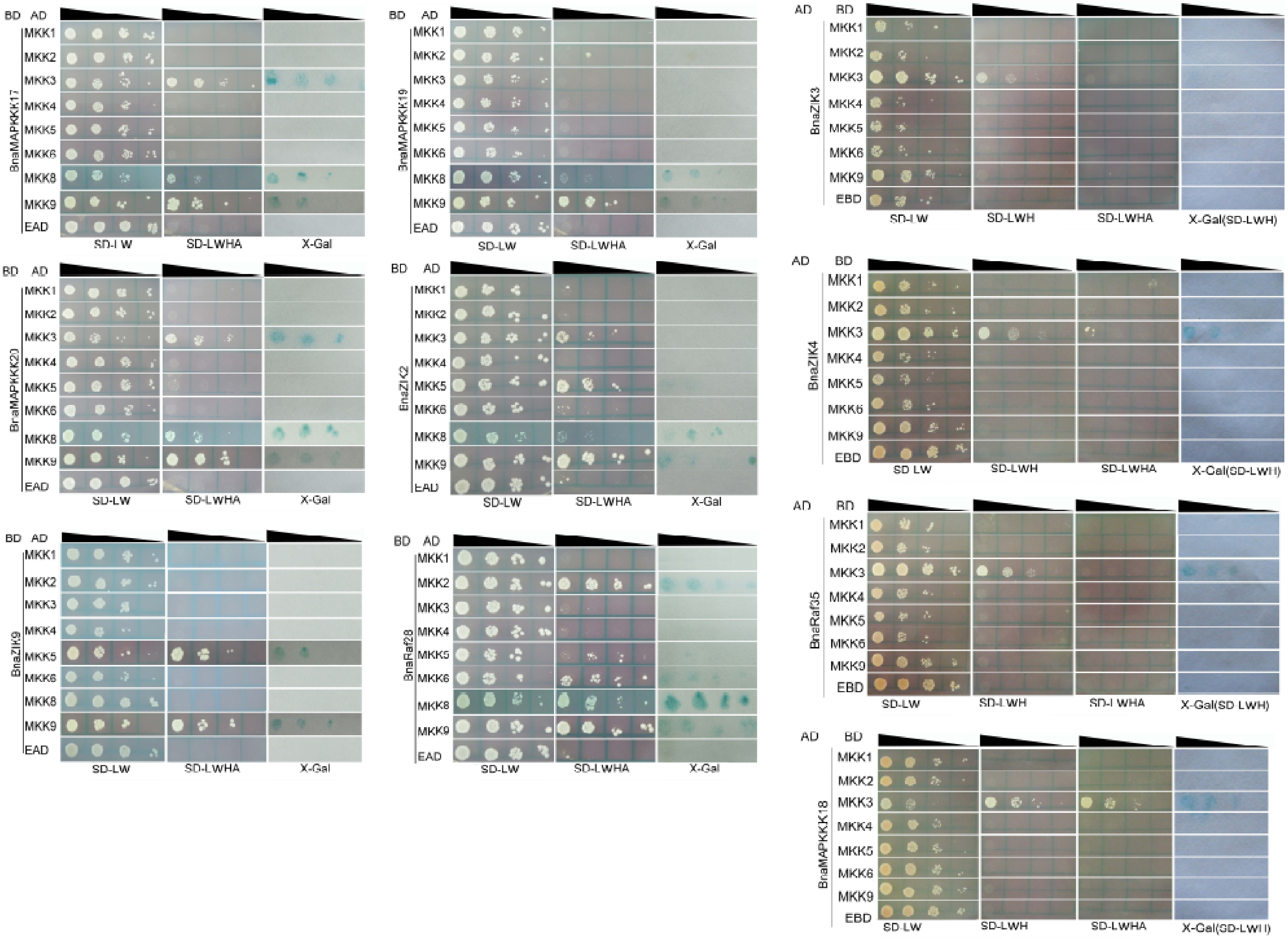

BD AD
BnaMAPKKK17
MKK1
MKK2
MKK3
MKK4
MKK5
MKK6
MKK8
MKK9
EAD
SD-LW
SD-LWHA
X-Gal
BD AD
BnaMAPKKK19
MKK1
MKK2
MKK3
MKK4
MKK5
MKK6
MKK8
MKK9
EAD
SD-LW
SD-LWHA
X-Gal
AD BD
BnaZIK3
MKK1
MKK2
MKK3
MKK4
MKK5
MKK6
MKK9
EBD
SD-LW
SD-LWH
SD-LWHA
X-Gal(SD-LWH)
BD AD
BnaMAPKKK20
MKK1
MKK2
MKK3
MKK4
MKK5
MKK6
MKK8
MKK9
EAD
SD-LW
SD-LWHA
X-Gal
BD AD
BnaZIK2
MKK1
MKK2
MKK3
MKK4
MKK5
MKK6
MKK8
MKK9
EAD
SD-LW
SD-LWHA
X-Gal
AD BD
BnaZIK4
MKK1
MKK2
MKK3
MKK4
MKK5
MKK6
MKK9
EBD
SD-LW
SD-LWH
SD-LWHA
X-Gal(SD-LWH)
BD AD
BnaZIK9
MKK1
MKK2
MKK3
MKK4
MKK5
MKK6
MKK8
MKK9
EAD
SD-LW
SD-LWHA
X-Gal
BD AD
BnaRaf28
MKK1
MKK2
MKK3
MKK4
MKK5
MKK6
MKK8
MKK9
EAD
SD-LW
SD-LWHA
X-Gal
AD BD
BnaRaf35
MKK1
MKK2
MKK3
MKK4
MKK5
MKK6
MKK9
EBD
SD-LW
SD-LWH
SD-LWHA
X-Gal(SD-LWH)
AD BD
BnaMAPKKK18
MKK1
MKK2
MKK3
MKK4
MKK5
MKK6
MKK9
EBD
SD-LW
SD-LWH
SD-LWHA
X-Gal(SD-LWH)

Figure S5. Yeast two-hybrid (Y2H) assay of interactions between MAPKKK and MKK proteins in canola. The yeast strain AH109 harboring the indicated plasmid combinations was grown on either the nonselective (SD-LW) or selective (SD-LWHA) media, followed by X-Gal staining. The remaining 18 BnaMAPKKKs did not interact with any of the eight BnaMKKs assayed and therefore are not shown here. Decreasing cell densities in the dilution series are illustrated by narrowing triangles. EAD and EBD are empty pGADT7 and pGBKT7 vector, respectively.

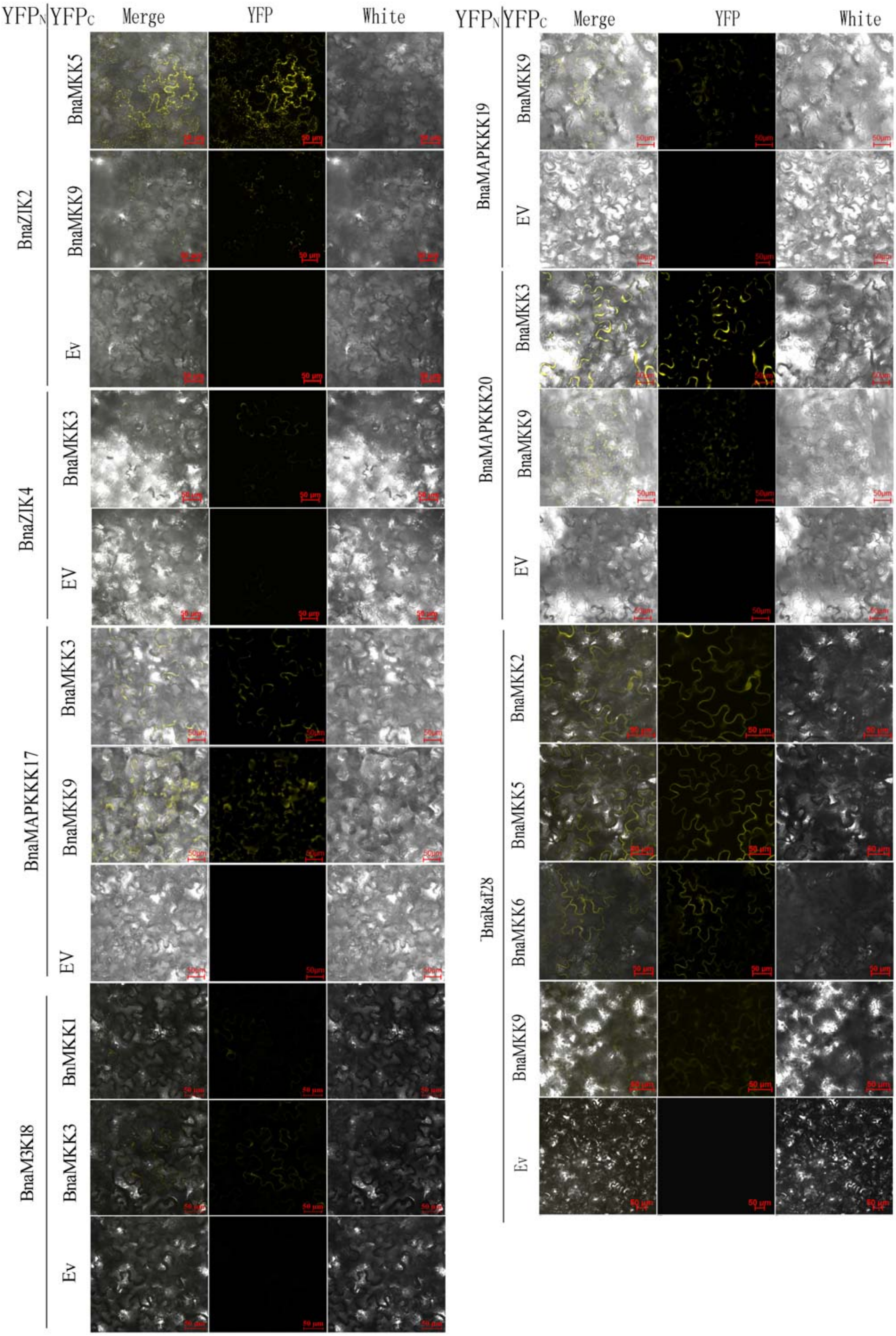
YFPN YFPC
Merge
YFP
White
BnaZIK2
BnaMKK5
BnaMKK9
Ev
BnaZIK4
BnaMKK3
EV
BnaMAPKKK17
BnaMKK3
BnaMKK9
EV
BnaM3K18
BnMKK1
BnaMKK3
Ev
BnaMAPKKK19
BnaMKK9
EV
BnaMAPKKK20
BnaMKK3
BnaMKK9
EV
BnaRaf28
BnaMKK2
BnaMKK5
BnaMKK6
BnaMKK9
Ev
50 μm

Figure S6. Analysis of BnaMAPKKK and BnaMKK interactions in *N. benthamiana* leaf epidermal cells through bimolecular fluorescence complementation (BiFC). BnaMAPKKK17, 19, 20, ZIK2, Raf28 was fused to N- terminal while BnaMKK2, -3, -5, -6 and -9 were fused to C-terminal halves of YFP, individually. The fluorescence of YFP in leaf discs was observed 3-4 d after agroinfiltration by confocal laser microscopy. Bar = 50 μm.

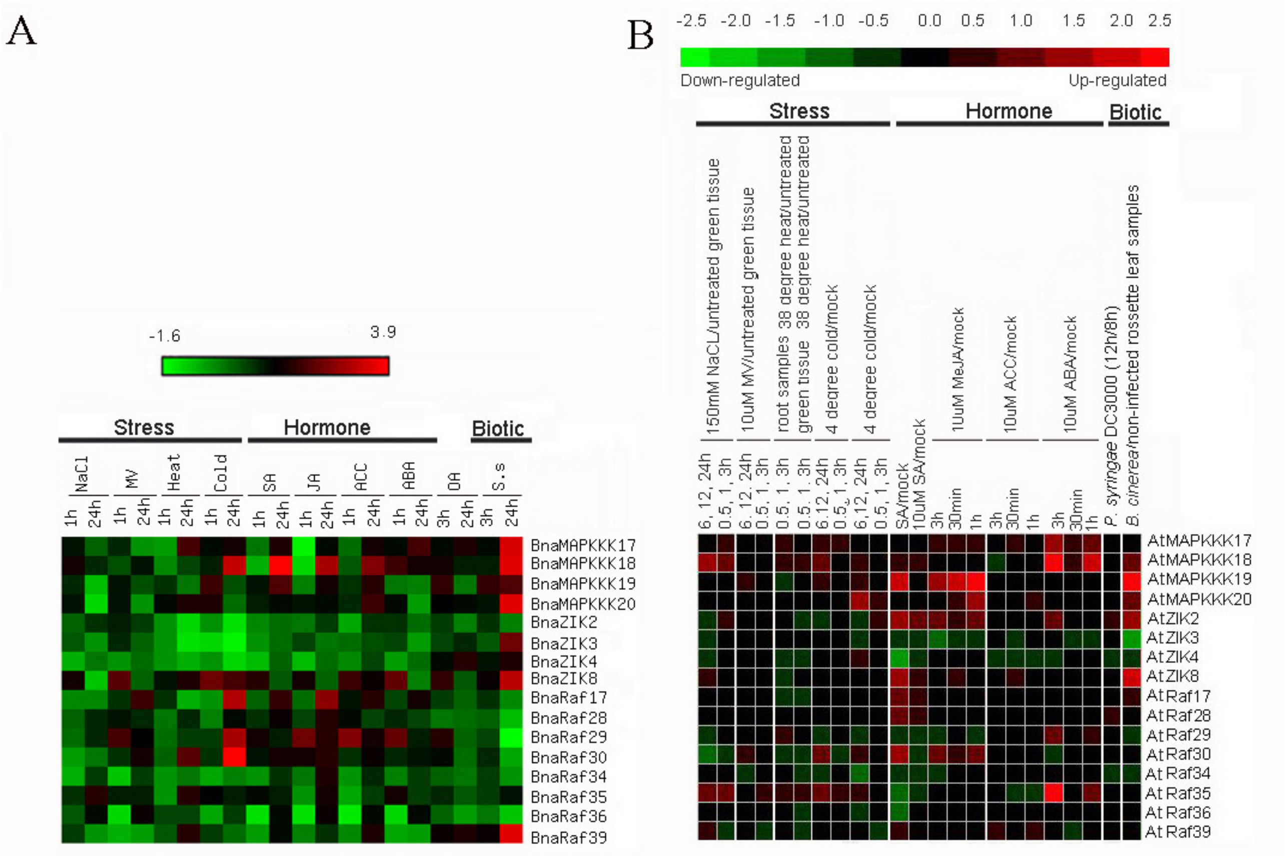

Figure S7. The heat maps of the expression profiles of MAPKKK genes of canola and Arabidopsis in response to abtioic and biotic stresses. (A) The expression ratios (treatment/mock, $\log_2$ scale) of 16 BnaMAPKKK genes in response to abiotic and biotic stress derived from our quantitative RT-PCR were transformed into heat map using Matrix2png (http://www.chibi.ubc.ca/matrix2png/bin/matrix2png.cgi). (B) The expression ratios (treatment/mock, $\log_2$ scale) of 16 AtMAPKKK are derived from Genevestigator (https://www.genevestigator.com/gv/plant.jsp), which contains Arabidopsis ATH1 GeneChip analysis data. The up-regulated expression was indicated in the red rectangle while the down-regulated expression in the green rectangle shape.

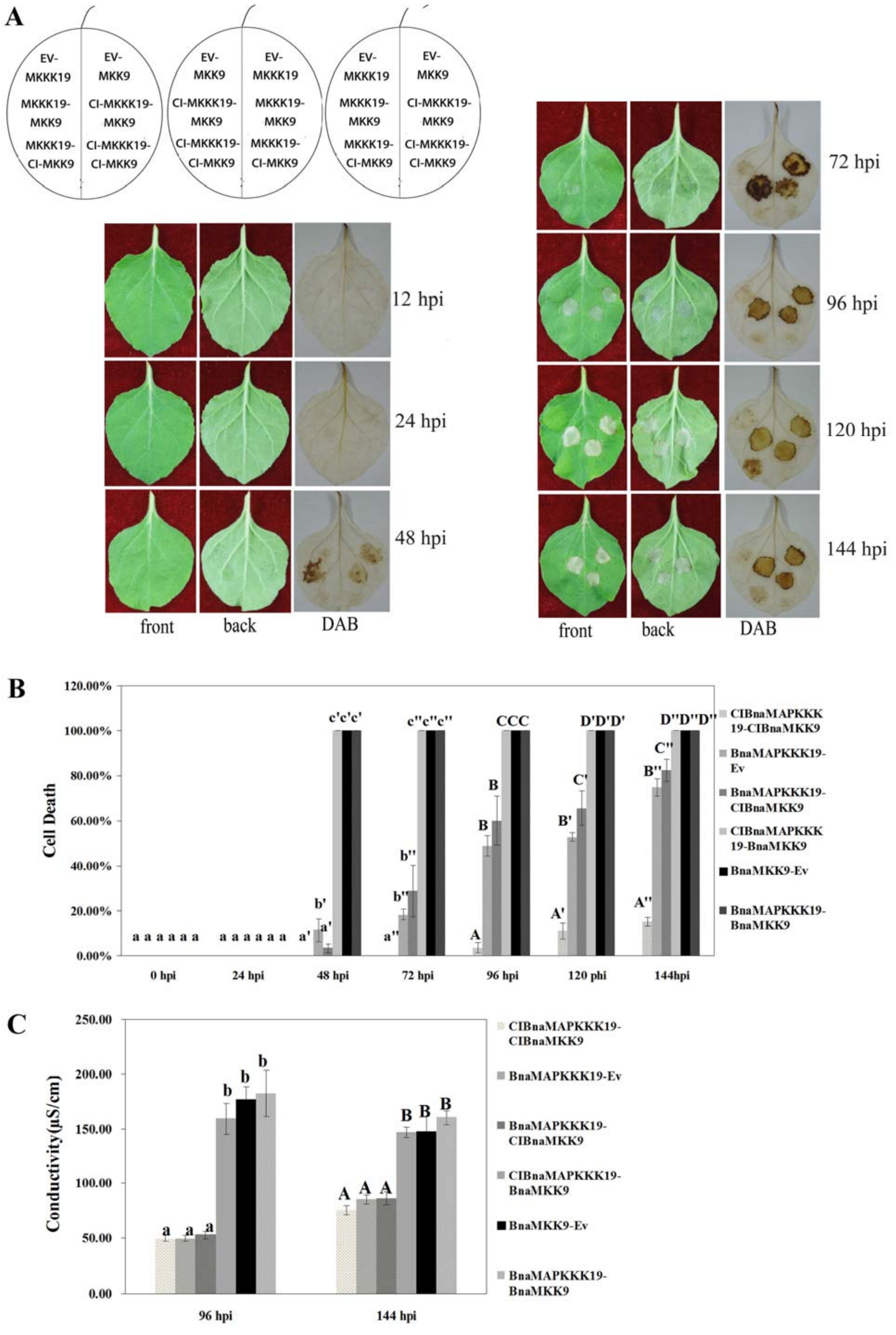

Figure S8. Co-expression analysis of *BnaMAPKKK19* and *MKK9* in eliciting pathogen-independent cell death in *N. benthamiana* leaves.

Leaves were infiltrated with agrobacteria carrying wild-type genes or mutated versions or both. All experiments were performed three times with similar results obtained. (A) Symptoms of *N. benthamiana* leaf areas expressing *BnaMAPKKK19, BnaMKK9* or their constitutively inactive (CI) mutant forms 12, 24, 48, 72, 96, 120 and 144 hours post-infiltration (hpi). EV represents the pYBHA binary vector harboring GFP gene under double CaMV35S promoter. The left, middle and right panels represent the front, back sides and DAB staining, respectively. The scheme at the top left shows the injection arrangement of leaves, in which three combinations at each half of a leaf. Equal amount of agrobacterial resuspension (1:1:1, volume ratio) in AS medium was injected into each site. (B) Quantification of cell death in *N. benthamiana* leaves expressing *BnaMAPKKK19, BnaMKK9* or their CI forms by examining the percentages of leaf sites with water-soaked symptom at various time-points. (C) Measurement of electrolyte leakage in leaf discs expressing *BnaMAPKKK19, BnaMKK9* or their CI forms at 96 and144 hpi. Values represent the means of three independent assays for each time-point ± S.E. Identical and different letters represent non- and significant difference ($p \leq 0.05$).
